# From stars to molecules: AI guided device-agnostic super-resolution imaging

**DOI:** 10.1038/s41467-026-75584-7

**Published:** 2026-07-16

**Authors:** Dominik Vašinka, Filip Juráň, Jaromír Běhal, Miroslav Ježek

**Affiliations:** Department of Optics, Faculty of Science, Palacký University, Olomouc, Czechia

**Keywords:** Super-resolution microscopy, Super-resolution microscopy, Imaging techniques

## Abstract

Super-resolution imaging has revolutionized the study of systems ranging from molecular structures to distant galaxies. However, existing super-resolution methods require extensive calibration and retraining for each imaging setup, limiting their practical deployment. We introduce a device-agnostic deep-learning framework for super-resolution imaging of point-like emitters that eliminates the need for calibration data or explicit knowledge of optical system parameters. Our device-agnostic modeling utilizes diverse, numerically simulated dataset encompassing a broad range of imaging conditions, enabling generalization across different optical setups. Once trained, the model reconstructs super-resolved images directly from a single resolution-limited camera frame with superior accuracy and computational efficiency compared to state-of-the-art methods. We experimentally validate our approach using a custom microscopy setup with controllable ground-truth emitter positions. We also demonstrate its versatility on stellar astronomy and single-molecule localization microscopy datasets of point-like sources, achieving high resolution without prior information. Our findings establish a pathway toward universal, calibration-free super-resolution imaging, expanding its applicability across scientific disciplines.

## Abstract (web summary)

From distant stars to single molecules, device-agnostic AI can reveal details blurred by ordinary optics. The approach works across imaging systems, helping make super-resolution more widely usable in science.

## Introduction

Optical resolution is fundamentally constrained by diffraction. This limits the ability to observe structures comparable to the wavelength of light divided by the numerical aperture of the imaging system, i.e., the famous Rayleigh resolution limit^1^. Super-resolution imaging has emerged as a transformative technique in disciplines ranging from biomedical^2,3^ and materials science^4^ to astronomy^5,6^, by circumventing this limit and revealing previously inaccessible structural details. Numerous applications rely specifically on imaging point-like or single-emitter sources, respective to the resolution limit. In biology, single-molecule localization microscopy enables nanoscale visualization of cellular structures^7–12^. Quantum physics leverages super-resolution for characterizing quantum dots^13^ and precisely imaging cold atoms in optical lattices^14,15^. Astronomy benefits through resolving individual stars and galaxies^16,17^.

Despite these successes, super-resolution methods require precise calibration and extensive knowledge of optical parameters. This requirement presents a substantial bottleneck, often involving laborious measurements, calibration data acquisition, and computationally expensive model retraining^10,18^. Furthermore, practical applications frequently struggle with inhomogeneities and instabilities in imaging conditions, which further limit the versatility of existing algorithms^19–21^.

To address these fundamental challenges, we propose a strategy for the data-driven deep-learning frameworks that is inherently device-agnostic, eliminating the need for calibration or prior system-specific information. Our method exploits numerically simulated data encompassing a wide range of optical conditions, enabling extreme generalization and adaptability across different imaging setups. Once trained, the device-agnostic model delivers rapid and accurate super-resolution reconstructions directly from a single, diffraction-limited image.

In this work, we demonstrate the efficacy of our approach through computational evaluations and experimental validations across multiple benchmarks, outperforming statistical Bayesian approaches and deep-learning-based state-of-the-art methods. We further confirm the practical advantages and broad applicability of our approach across diverse datasets, ranging from localization microscopy to stellar astronomical imaging of point-like sources. The developed framework paves the way towards universal, calibration-free super-resolution imaging, significantly enhancing its accessibility and impact across scientific and technological disciplines. Moreover, the device-agnostic training paradigm is not tied only to imaging and can be applied to any application where broader generalization ability is a beneficial or even crucial feature.

Super-resolution imaging can be achieved through various approaches, such as linear inverse and statistical Bayesian algorithms^22^, sparse representation^23^, tomographic image synthesis^11,24,25^, and methods based on blinking emitters^7–9^. Many deep learning super-resolution methods have been developed in the last decade^26–30^. Recently, artificial intelligence has been used to identify novel super-resolution microscopy setups^31^.

Regardless of the specific approach, super-resolution imaging can generally be classified into two categories: reconstruction and parameter estimation. Reconstruction aims to directly restore the whole super-resolved image^22,27^, while maximizing the fidelity to the underlying structure. In contrast, estimation focuses on extracting key parameters and features, such as emitter localization^29,32,33^. The localization performance, often emphasizing minimal false-positive detections at the cost of information loss, rapidly decreases for images with a higher number of overlapping emitters, which typically limits its application to stochastically blinking or photoactivated samples^34–36^. In the following discussion, we will focus exclusively on the former: image reconstruction.

Achieving accurate reconstruction of an object based on a single intensity image requires substantial knowledge about the imaging system under consideration. Central to this knowledge is an accurate estimation of the point spread function, which characterizes the response of the system to a point source of light. The reconstruction quality is inherently linked to the accuracy of the point spread function estimate; any inaccuracies will lead to poor reconstruction results^37^. However, its precise estimation entails additional calibration measurements involving an isolated emitting point source. This condition can prove demanding, especially in systems with high concentration of emitters, low signal-to-noise ratio, or temporally developing systems. While procedures for in-situ point spread function estimation for room-temperature microscopes exist^38,39^, these methods require an additional calibration step and are not feasible across domains, such as imaging of astronomy objects, cryostat-positioned semiconductor quantum dots, or electron microscopy. Additionally, imaging systems commonly exhibit variations in the shape of the point spread function across the image. Consequently, reconstructing larger areas may require separate calibration for each segment^38^.

Some approaches can be adapted to perform a blind reconstruction, operating without explicit prior knowledge of the point spread function^40^. Instead, these methods impose additional assumptions on the imaging system, typically in the form of a predefined point spread function shape. During reconstruction, parameters of the point spread function, such as its width or other defining characteristics, are iteratively estimated directly from the input image. As a result, these methods introduce imperfections by simultaneously reconstructing the observed object and the point spread function of the imaging system. Altogether, blind reconstruction remains a non-convex optimization problem, posing a challenging task in real-life scenarios and often yielding inferior results compared to non-blind algorithms.

Similar device-dependent constraints arise in data-driven deep learning reconstruction. These approaches often outperform classical algorithms in both reconstruction and parameter estimation tasks^26,27,29,30^ and highly improve the processing of large astronomical surveys, as emphasized by the fast inference speed after training^41–48^. Yet, their calibration poses an even greater challenge. Unlike traditional methods, a data-driven deep learning model infers the reconstruction mapping by extracting relevant information from observed samples. However, due to the device-dependent nature of a typical training process, the model can only be applied to a particular point spread function profile and additional conditions, such as emitter power, background noise distribution, and concentration of emitters, each requiring prior estimation from the calibration datasets^49^. Modifying these parameters requires remeasuring the calibration set and retraining the model, making it a resource-expensive procedure. Several approaches have moved beyond purely data-driven training by incorporating physical models into the deep learning framework. Examples include plug-and-play methods, which combine learned denoisers with classical iterative reconstruction schemes, and deep unrolling, which embeds the imaging algorithm into a trainable, iteration-inspired architecture^50,51^. By integrating explicit physical priors, these hybrid approaches can improve robustness and interpretability compared to fully data-driven models. At the same time, the priors make these strategies even more model-dependent and less generalizable. Finally, reconstruction in astronomical observations may benefit from using multiple input frames of the same region^52^, enabling gradual improvement in reconstruction quality with each additional frame^53^, or from capturing images at different wavelengths^54^. However, such techniques are beyond the scope of the targeted single-frame domain.

## Results

### Device-agnostic modeling

**Fig. 1:**
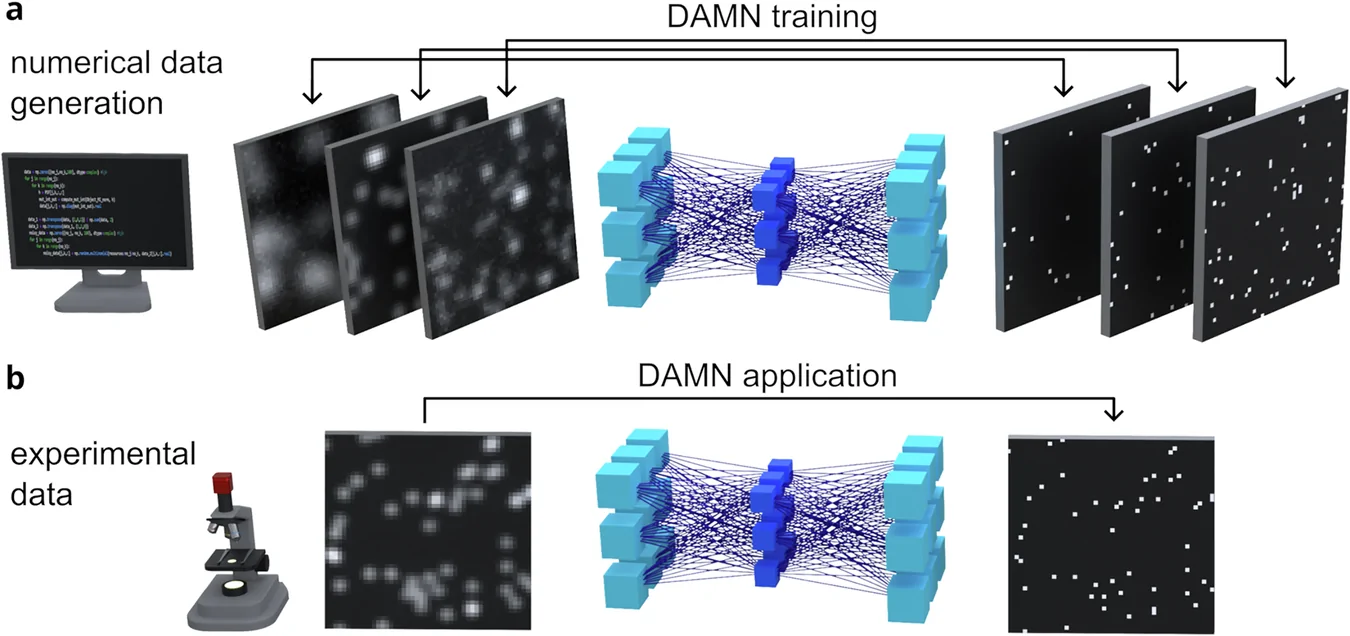
Schematic representation of the device-agnostic modeling approach and its application to intensity images of point-like emitting sources. **a** The model is trained using numerically simulated data pairs comprising resolution-limited noisy images alongside their super-resolved counterparts. Each training sample represents a unique combination of underlying optical parameters, such as the width of the point spread function and the signal-to-noise ratio. **b** Following training, this model is applied to enhance the resolution of experimental images acquired using a real-life imaging system.

We developed a strategy for training device-agnostic neural networks. The framework presented in this study is not tied to a single specific model but can be readily applied to any neural network architecture. We use it to train a device-agnostic modeling network (DAMN) designed to reconstruct intensity images of point-like emitting sources, as illustrated in Fig. 1. Unlike traditional approaches, a DAMN model operates using a single intensity frame without requiring additional information about the sample and optical parameters of the imaging system. Without the need for calibration measurements or retraining, it can directly process images from diverse applications. Our model leverages the fully convolutional architecture of a neural network (refer to the Methods section for details), enabling it to reconstruct frames of varying shapes and sizes. Moreover, the reconstruction process is performed by a single-pass propagation through the neural network, resulting in rapid reconstruction of super-resolved images.

We train the DAMN model using pairs of targeted ideal objects and their corresponding resolution-limited images generated through numerical simulation. The ensemble of data pairs covers various optical parameters, such as emitter power and concentration, background noise distribution, and even different shapes and widths of the point spread function, including various asymmetries. Their ranges were selected to cover the majority of realistic situations (additional details in the Methods section). The extent of the synthetic training data exceeds the conventional approaches in the astronomy or single-molecule microscopy domains. Subsequently, we utilized incremental learning techniques^55^ for the model to acquire knowledge about the widest possible parameter combinations. As a result of the optimization and training process, the trained model accurately reconstructs super-resolved images independently of the underlying imaging parameter values. Consequently, without the need for retraining or calibration data, the model can be applied to diverse imaging systems and is robust to their parameter changes, including inhomogeneity across a field of view and instability, such as temporal variations and fluctuations. Moreover, the DAMN model demonstrates a high robustness to optical aberrations as characterized in the Supplementary Aberration analysis section.

### Evaluation using simulated datasets

To characterize the effectiveness of device-agnostic modeling, we conduct a comparative analysis with established reconstruction algorithms. Namely, we implemented the Richardson-Lucy deconvolution based on Bayesian estimation with a uniform prior^56,57^, and its variant utilizing a total variation regularization^58^ (see Methods for details). These widely used iterative algorithms reconstruct images by repeatedly utilizing a known point spread function. We also compare our results against Deep-STORM^27^, a deep-learning method for image reconstruction in localization microscopy (see Supplementary Deep-STORM section). This device-specific convolutional network is trained on simulated data with imaging parameters that precisely match the testing regimes, including point-spread-function profile, signal-to-noise ratio, and emitter density. Therefore, all benchmarking approaches have the advantage of utilizing prior information about the imaging setup.

We evaluate the results over a wide range of optical parameters to demonstrate the device-agnostic generalization ability using separate test sets of image pairs not included in the training data. We quantify the performance of all methods using the mean absolute error between the reconstructed image and its target object, averaged across each test dataset. In contrast to both Richardson-Lucy algorithms and Deep-STORM, which require (and were supplied with) prior knowledge of the imaging system, our DAMN model operates independently of any device-specific information. Despite this distinction, the device-agnostic model (without prior information) outperforms the alternative approaches in terms of reconstruction quality, as discussed in the following text.

**Fig. 2:**
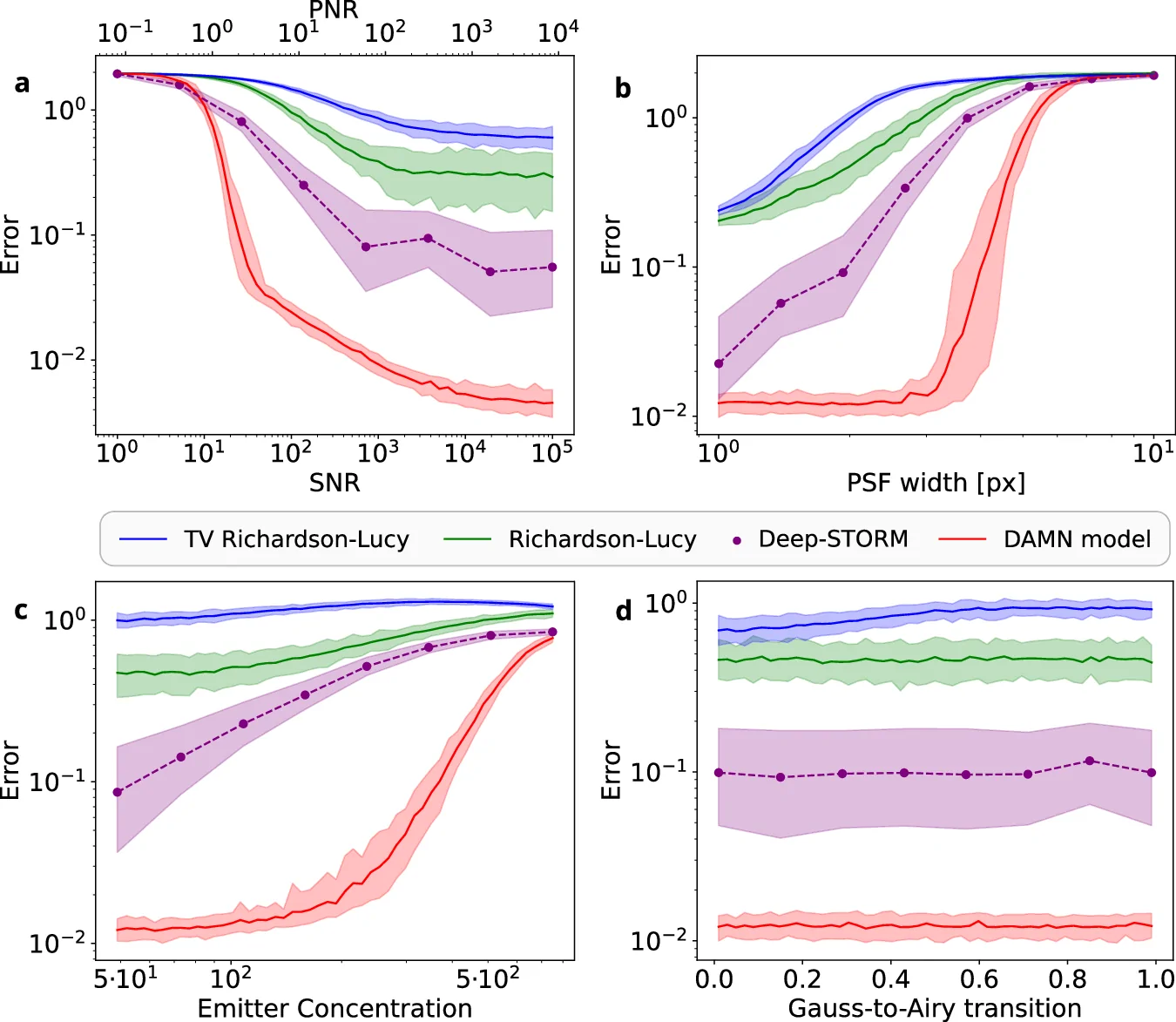
Performance of super-resolving methods on simulated data. The dependence of the mean absolute error on **a** the signal-to-noise ratio (SNR), **b** the width of a Gaussian point spread function (PSF), **c** the number of emitters in the image (concentration), and **d** the continuous transition between a Gaussian and an Airy PSF, respectively. The resulting averages of the Richardson-Lucy algorithm (green), its variant with total variation regularization (blue), individual Deep-STORM neural networks (purple), and the DAMN model (red) are accompanied by their 90% confidence intervals over the test set. Panel **a** is provided with a secondary horizontal axis recalculating the SNR values to the peak-to-noise ratio (PNR). Across all panels, the DAMN model consistently outperforms the alternative approaches by up to two orders of magnitude. The optical parameters not investigated in a given panel have the following values: SNR = 500, the average noise intensity = 10, the concentration = 50, and the Gaussian PSF with *σ* = 2 px.

Panels a–c in Fig. 2 illustrate the dependence of mean absolute error on the emitter power, the width of the point spread function, and the concentration of emitters, respectively. The results of these log-log graphs are characterized using an average value over the test dataset with a corresponding 90% confidence interval. Panel a contains dual horizontal axes representing the varying emitter power using both the signal-to-noise ratio (SNR) and the peak-to-noise ratio (PNR). For additional details regarding the explored parameters and evaluation of both approaches, see the Methods section. The DAMN model, represented by the red color, exhibits significantly better performance than the blue- and green-colored Richardson-Lucy algorithms and even surpasses the purple Deep-STORM. Similar results can also be observed in panels b and c. Panel b depicts the error dependence on the point spread function width (consult the Methods section). The remaining panel c characterizes the dependence on the emitter concentration, referring to the collective number of emitters in a single image. As seen across all three panels, our device-agnostic approach provides more accurate reconstructions than the alternatives across all testing regimes. Moreover, each Deep-STORM graph point corresponds to an individually trained neural network that utilizes precise knowledge of the test imaging parameters. The depicted errors were evaluated across the whole image and can be, alternatively, expressed as per-pixel errors by dividing the values by the number of pixels.

The panel d in Fig. 2 visualizes the error dependence on the point spread function shape, gradually transitioning from a Gaussian profile to an Airy pattern of the same width. As seen from this semi-logarithmic graph, the performance of all methods remains approximately constant during this transition. Such behavior is expected from the device-specific approaches, as we always provide them the correct point spread function profile. On the other hand, the DAMN model was trained solely on the exact Gaussian and Airy point spread functions, each constituting approximately half of the dataset. Despite this simplification in the training process, the graph unambiguously demonstrates the adaptive ability of the DAMN approach to generalize to previously unseen point spread function shapes. Moreover, the DAMN model exhibits strong robustness against optical aberrations in the image (as discussed in the Supplementary Aberration analysis section), despite never seeing them during training. This adaptability is beneficial, as it significantly reduces the data and simulation complexity required for training device-agnostic models. Altogether, the presented panels demonstrate numerous benefits of the DAMN approach, proving it superior to the established algorithms.

### Optical microscopy experimental validation with partial control over ground truth

**Fig. 3:**
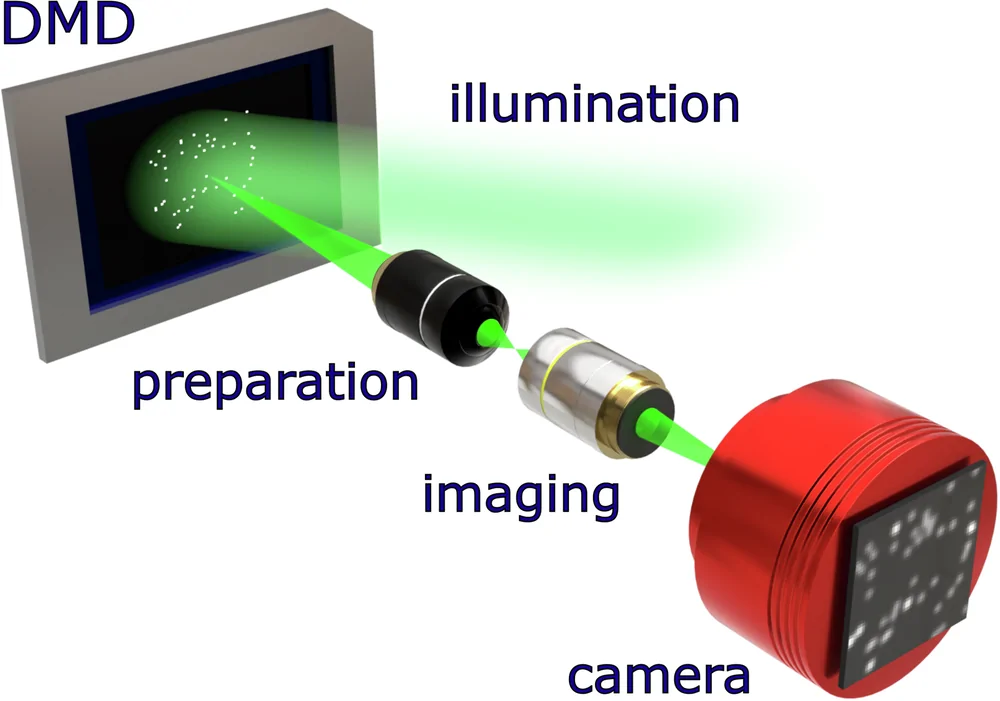
Schematic illustration of the optical setup used to collect experimental data pairs. A ground-truth mask is imposed on the digital micromirror device (DMD) by configuring its mirrors. An incoherent illumination light reflected by these mirrors impinges a high-resolution preparation system. The DMD-imposed mask is re-imaged into the front sample plane of the preparation system, creating point-like emitters with the intended spatial distribution. The imaging part of the setup, comprised of a low-resolution microscope objective, images the sample-plane emitters onto a camera. The resulting camera-captured intensity image and the DMD-imposed mask represent the experimental data pairs.

To validate our approach beyond simulated data, we applied it to reconstruct experimentally acquired images. Such data inherently lack ground truth information, which prevents the use of most quantitative evaluation metrics. To bridge the gap between purely simulated and real-world, but fully uncontrolled, experimental data, we developed a custom-built optical microscopy setup, illustrated in Fig. 3 and shown in detail in Supplementary Fig. 1. Unlike the traditional super-resolution benchmarking, this microscope provides partial control over the spatial distribution of emitting point-like sources and complete ground-truth information about their positions. An incoherent light illuminates a digital micromirror device (DMD), onto which a mask representing the targeted ground-truth is imposed. By configuring the micromirrors, we prepare a mask containing the targeted number of sources positioned at desired locations. As such, the control is limited to activating emitting sources at discrete grid positions. This mask is then re-imaged by a high-demagnification and high-resolution preparation system, creating point-like emitters and thus forming a sample object. Subsequently, a low-resolution imaging microscope projects the emitters onto a camera. The resulting intensity profile captured by the camera serves as the resolution-limited input for reconstruction methods. Together with the ground-truth mask, this setup provides experimental test data pairs, which allow for exact metric quantification of each reconstruction method performance. The collected dataset does not fully replicate or substitute real microscopy conditions due to its restriction to two-dimensional grid distributions and uniform illumination. However, it represents an essential link and intermediate middle-point between idealized simulations with complete control and knowledge over ground truth and fully experimental data lacking both ground truth knowledge and control. For further details on the optical setup, see the Supplementary Experiment section.

**Fig. 4:**
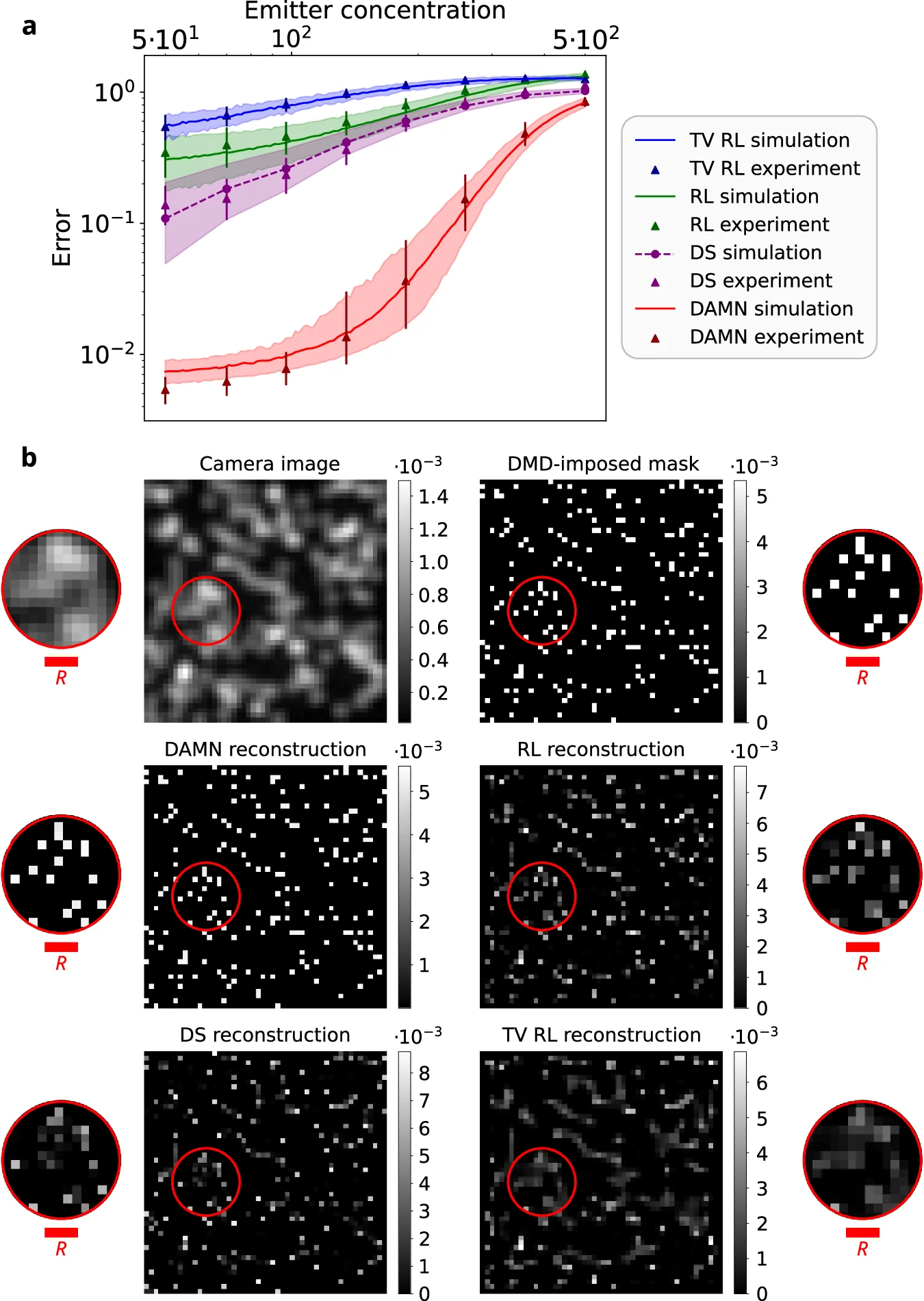
Performance of super-resolution methods on experimental data with the partial control and complete knowledge of ground truth. **a** The colored dots represent the mean absolute error between the DMD-imposed masks and the super-resolved images reconstructed by each method: Richardson-Lucy (RL, blue), Total variation Richardson-Lucy (TV RL, green), the device-agnostic model (DAMN, red), and individual Deep-STORM models (DS, purple). These errors were evaluated across various emitter concentrations with their 90% confidence intervals. The accompanying continuous lines depict error values derived from simulated data using optical parameters estimated for our imaging system. **b** A typical camera image containing nearly 200 emitters, alongside its corresponding DMD-imposed mask and reconstructions from each method. The circled areas contain a magnified region for easier visual comparison. It is evident that the DAMN model significantly outperforms the alternative approaches, even in regions where the mutual emitter distance is well below the Rayleigh resolution limit *R* = 3.9 px (inset scale bars).

Using this setup, we investigated the performance of all methods while varying the concentration of emitters. Panel a in Fig. 4 illustrates the mean absolute error between reconstructed images and their corresponding ground-truth masks using the experimental data, shown as dot markers. For comparison, the continuous lines represent the results obtained using simulated data with corresponding optical parameters. For Deep-STORM, results on simulated data are also illustrated as scatter points, since each requires a separately trained neural network. The DAMN model outperforms both Richardson-Lucy deconvolutions by orders of magnitude. Similarly, it achieves better reconstruction performance than the device-specific Deep-STORM, even when applied to images acquired from experimental measurements. This improvement is achieved despite operating significantly below the Rayleigh resolution limit of our imaging system. To further emphasize these findings, panel b in Fig. 4 shows a representative experimental image containing nearly 200 emitters and its reconstruction by each method. As observed, the DAMN model provides a near-perfect reconstruction of the original image without any calibration. The two closest resolved emitters are 1 px apart, even in dense clusters, which is a distance almost 4 times below the Rayleigh resolution limit, *R* = 3.9 px. (The resolution improvements for reconstructions with an upsampling DAMN model are evaluated in the following section.) In contrast, the other methods exhibit visible deviations and artifacts even with full access to the imaging parameters. This experimental demonstration highlights the benefits of our device-agnostic modeling approach, which significantly outperforms the established algorithms that rely on prior knowledge of imaging parameters.

### Stellar astronomy and localization microscopy demonstration

Since real applications benefit from the highest possible reconstruction precision, we also applied the device-agnostic modeling to train an additional network with upsampling layers in its architecture (see Methods). This DAMN model inherently provides an eightfold increase to reconstructed spatial dimensions, allowing reconstruction on a finer image grid. In this section, we used this upsampling model on three distinct experimentally acquired datasets of point-like sources ranging from stellar astronomy to single-molecule microscopy. All presented results were obtained without any recalibration or retraining of the DAMN model. Moreover, no pre-processing was applied to the measured images.

**Fig. 5:**
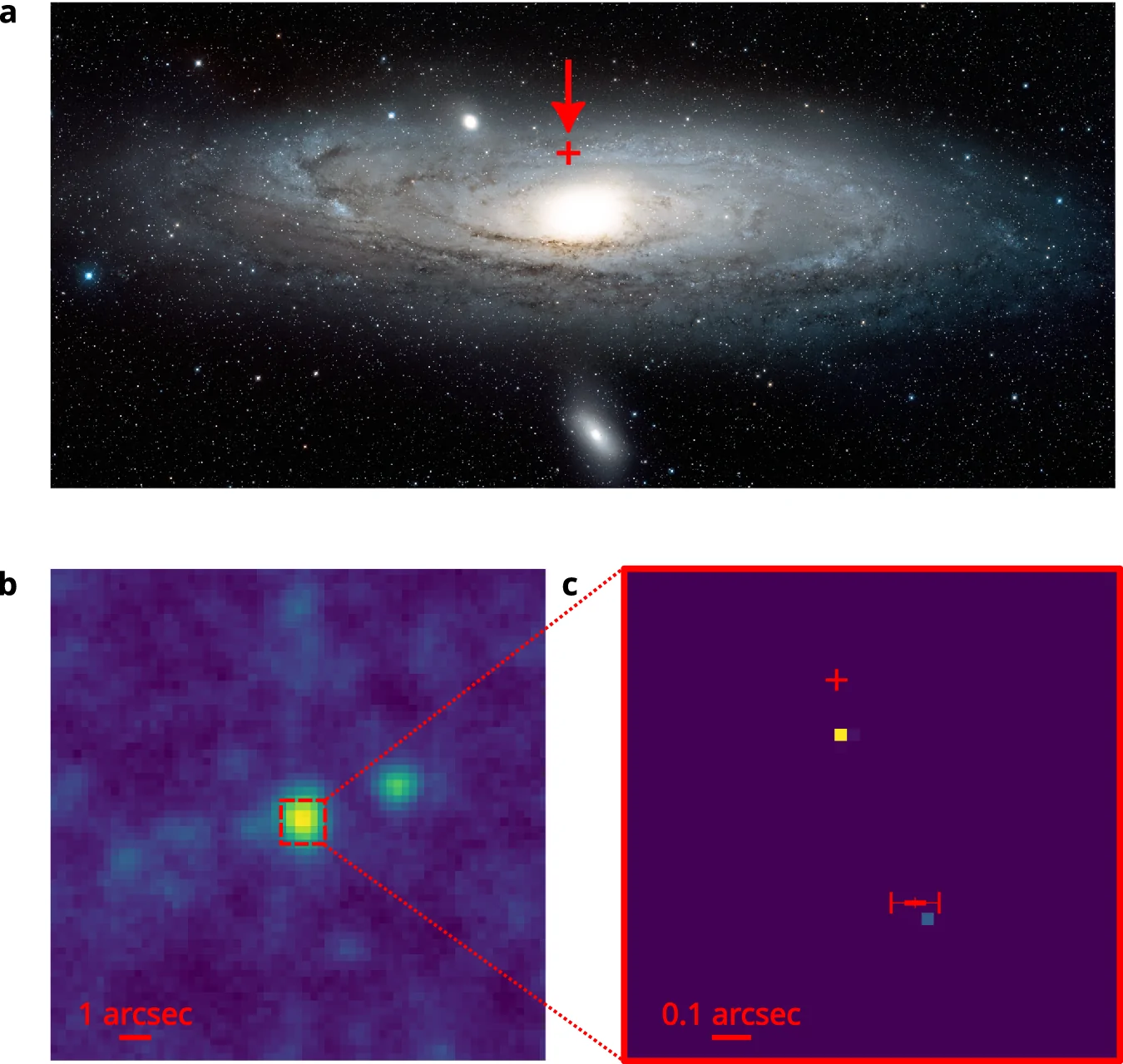
Demonstration of the super-resolving capabilities of the DAMN model on an astronomical image of a dense stellar field. **a** Contextual wide-field view of the Andromeda galaxy (M31) by ESA/Hubble^61^, rotated and cropped. The location of the analyzed region is indicated. **b** Zoomed-in outer field of M31 obtained with the ground-based Pan-STARRS1 telescope, showing an unresolved double-star system in the center. **c** High-resolution reconstruction of the same region produced by the DAMN model, further zoomed for visualization. Red markers indicate the star positions and their standard deviation uncertainties derived from Gaia DR3 astrometric data, based on the Gaia space-based observatory. Notably, one uncertainty is significantly larger as that star magnitude borderlines with Gaia sensitivity. The reconstruction does not use any calibration data or prior information on the employed imaging system.

First, we applied the DAMN model to a ground-based image of the outer field of the Andromeda galaxy (M31). The image was obtained with the Pan-STARRS1 wide-field survey telescope^59^. Within the field of view, a double-star system was identified using astrometric data from the Gaia Data Release 3^60^, based on the Gaia space observatory, which provides sub-pixel positional information for both components. The angular separation of the two stars, approximately 0.6 arcsec, lies well below the approximate 1.4 arcsec Rayleigh resolution limit of the Pan-STARRS1 imaging system. Consequently, the double-star system is fully unresolved in the original ground-based image, as shown in Fig. 5 b. Nonetheless, the DAMN model, having no explicit knowledge of the imaging parameters, still resolved the two distinct point-like sources, despite operating at nearly 2.5 times below the resolution limit. Panel c depicts the reconstructed region, zoomed to enhance visualization of the double-star system. As shown, both stars are clearly resolved, with an estimated angular separation of 0.52 arcsec. Therefore, the error of the double star distance reconstruction is approximately 0.08 arcsec, which is 17.5 times below the Rayleigh resolution limit. Moreover, the ratio of reconstructed intensities is 3.46, which is comparable to the targeted 3.65 based on Gaia. This result demonstrates the ability of the DAMN model to accurately and precisely recover sub-diffraction structures from experimentally acquired images. Fig. 5 a additionally shows a contextual wide-field image of the Andromeda galaxy by ESA/Hubble^61^ with the location of the double-star system indicated.

**Fig. 6:**
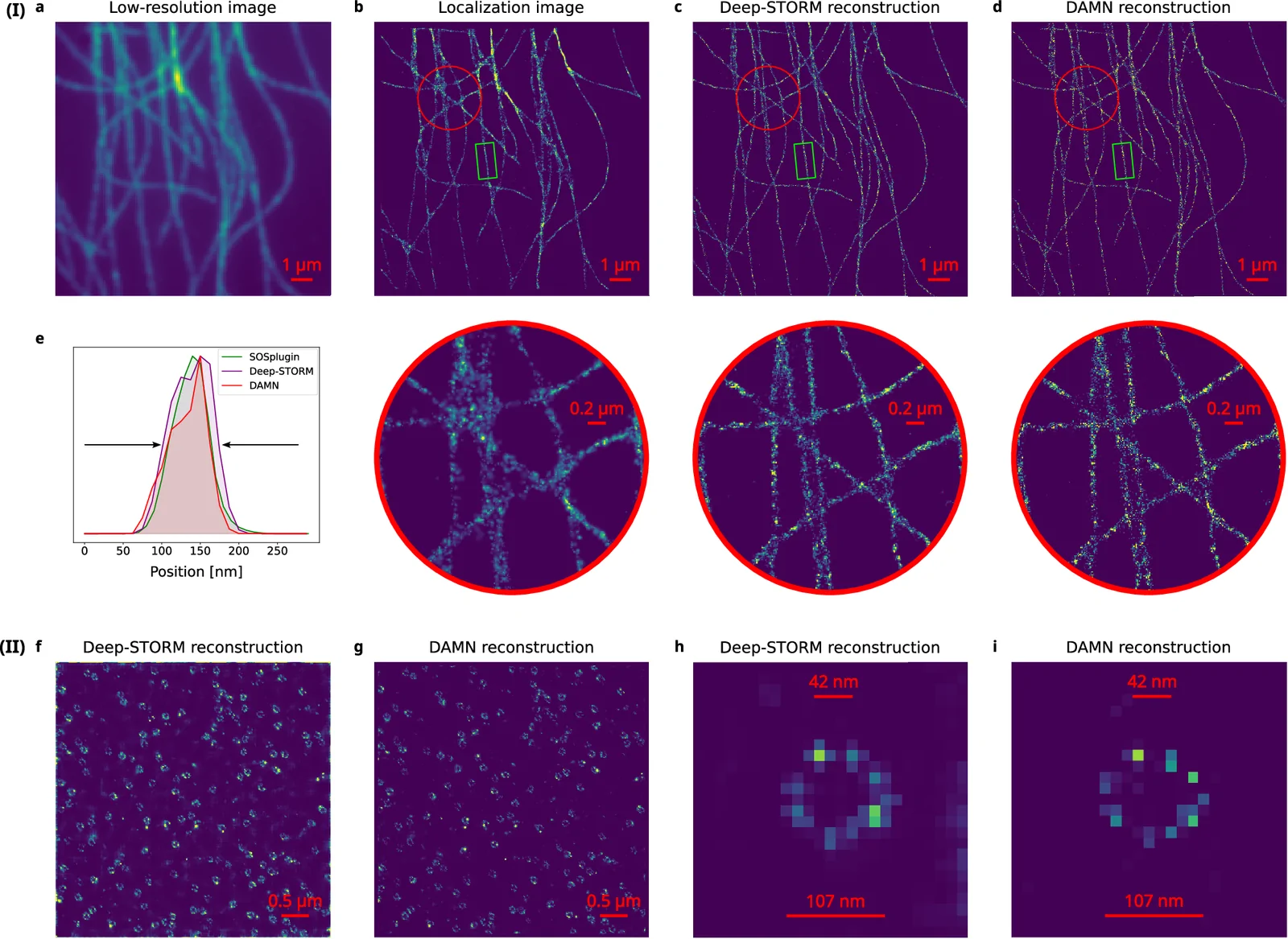
Demonstration of the DAMN model on (I) a high-concentration tubulin dataset from the single-molecule localization microscopy challenge, and (II) a nuclear pore complexes dataset. **(I) a** A low-resolution image integrated from the set of 500 measured images, containing numerous emitting molecules. **b** A reconstruction rendered from a localization table provided by the SOS Plugin. **c** A reconstruction provided by Deep-STORM, calibrated using detailed information on the imaging setup. **d** A super-resolved image produced from the calibration-free, device-agnostic model without prior information. Panels **b**–**d** include inset images with a magnified region highlighting the resolution details. **e** Projection of the microtubule profile over the green rectangle segment. The attained full width at half maximum was 56 nm for SOS Plugin, 73 nm for Deep-STORM, and 55 nm for the DAMN model. **(II)** Reconstruction of the nuclear pore complex dataset containing Nup96 labeled with BG-AlexaFluor647. **f** Large field-of-view reconstruction generated from the 50,000 frames produced by Deep-STORM, showing multiple nuclear pore complexes. **g** Corresponding large field-of-view reconstruction obtained with the DAMN model. **h** Close-up of a single nuclear pore complex reconstructed by Deep-STORM from a subset of 5000 drift-free frames. **i** Close-up of the same nuclear pore complex reconstructed by DAMN. Panels **h**, **i** also indicate the expected ring diameter and nearest-neighbor spacing of the characteristic octagonal geometry for reference.

We next applied the same DAMN model, without retraining, to a publicly available dataset of tubulin structures^62,63^, which was experimentally acquired for the single-molecule localization microscopy challenge. As such, the ground-truth emitter positions and intensities are not available for direct quantitative evaluation. Therefore, we assessed reconstruction quality by comparing it with established methods. Figure 6 (I) presents the performance evaluation using these 500 tubulin images (128 × 128 pixels) with high emitter density. Panel a shows the corresponding integrated low-resolution image. Panel b shows a reference reconstruction provided by the SOS Plugin^64^, a least-square localization method assuming a Gaussian point spread function, which participated in the challenge and is available as a plugin for ImageJ. In this reconstruction, each localized emitter is rendered as an upsampled Gaussian profile whose width corresponds to the localization uncertainty. Panel c depicts a tubulin reconstruction obtained with Deep-STORM, using a model specifically fine-tuned and trained for this dataset by the original authors^27^. Both reference approaches are device-specific and require additional prior information about the imaging parameters. In comparison, the DAMN model directly processes the dataset without calibration or prior knowledge and produces the super-resolved tubulin image, see panel d. The highlighted areas (red) show a close-up comparison of all three approaches.

Both DAMN and Deep-STORM accurately recover the underlying tubulin structure even in densely populated regions, achieving visual quality significantly surpassing that of the SOS Plugin localization method. For example, two vertically oriented, overlapping microtubules in the insets are clearly resolved by both deep-learning techniques. Moreover, a close inspection reveals that the DAMN reconstruction exhibits a sharper, more confined microtubule structure than Deep-STORM. Panel e visualizes the projection of a single microtubule profile over the green segment. For each method, we evaluated the full width at half maximum of the reconstructed microtubule profile as 56 nm for the SOS Plugin, 73 nm for Deep-STORM, and 55 nm for the DAMN model. These results indicate that the DAMN approach combines robust performance in high-density conditions with a spatial precision comparable to localization-based methods. Overall, this comparison highlights the reconstruction capability of device-agnostic models for challenging microscopy applications.

Finally, since no ground-truth information is available for the tubulin dataset, we also evaluated the methods using microscopy data with partial structural ground truth: nuclear pore complexes, whose architecture is well-characterized. Specifically, we utilized a dataset of the scaffold nucleoporin Nup96 labeled with BG-AlexaFluor647^65^. The nuclear pore complexes exhibit eightfold rotational symmetry, with Nup96 expected to occupy positions on a ring of approximately 107 nm in diameter and a nearest-neighbor spacing of 42 nm, given the labeling geometry. These distances are approximately 3.4 times and 8.6 times smaller than the Rayleigh resolution limit of 360 nm of the employed imaging setup, respectively. Therefore, the dataset represents a challenge for super-resolution reconstruction. Using 50,000 low-resolution frames, Fig. 6 (II) compares reconstructions obtained with Deep-STORM, trained using the estimated imaging parameters, and with the DAMN model. While both approaches successfully recover the octagonal arrangements, Deep-STORM exhibits stronger background noise across the field of view.

Moreover, a closer inspection of the dataset reveals a noticeable lateral drift over time, which could, in principle, be compensated for. However, since we want to compare the reconstruction performance of each method without additional post-processing, we instead selected a temporal subset of frames with negligible drift. Accordingly, we include panels showing a single nuclear pore complex reconstructed from 5000 consecutive frames. While the DAMN model provides a sharper image than the Deep-STORM, the recovered structures are consistent with the known geometry for both cases. Additionally, for the DAMN reconstruction, the estimated ring diameter is 100 nm, and the inter-emitter spacing is 38 nm, corresponding to distance errors of 7 nm and 4 nm, respectively. These results are the ultimate demonstration of the DAMN super-resolving ability. For comparison, the Deep-STORM reconstructed ring diameter is 98 nm, and the inter-emitter spacing is 38 nm, with the distance errors of 9 nm and 4 nm, respectively. We also evaluated the SOS localization plugin on this dataset. However, because it could not reliably localize a substantial fraction of emitters under these conditions, the resulting reconstruction quality was notably inferior to that of both deep-learning approaches. Therefore, it is not included in the figure.

## Discussion

We demonstrated a device-agnostic approach to super-resolution imaging of point-like emitting sources that utilizes deep learning techniques. The presented approach solves the long-standing problems of measuring calibration data and estimating parameters accompanying the training of a deep learning model. Using only numerically simulated data, we develop a model capable of reconstructing intensity images using a single frame without explicit knowledge of the imaging system. This model can be applied to images of arbitrary shape and size originating from various optical systems, and its calibration-free nature provides robustness against non-uniformity and temporal variations in the optical parameters, including optical aberrations in the image. We compared this device-agnostic model with well-established reconstruction algorithms, namely the classical and regularized Richardson-Lucy deconvolution algorithm, and the state-of-the-art deep learning Deep-STORM. The analysis results clearly demonstrate the superior performance of our approach, which outperforms benchmark methods by up to two orders of magnitude in mean absolute error. Moreover, we designed an advanced optical setup for acquiring experimental images of emitting sources together with their precise ground-truth references. Providing partial control and complete knowledge over the spatial distribution of emitters, this setup enables exact performance quantification using measured data, which, to the best of our knowledge, is unprecedented in the super-resolution imaging domain. Using these experimental data pairs, we verified the superior performance of our approach.

To further demonstrate the universality of our approach, we applied the model to astronomy and microscopy data of point-like sources, achieving significant improvements in resolution in both domains. We reconstructed high-resolution images of tubulin and nuclear pore complexes from single-molecule localization microscopy data. In astronomy, we enhanced the resolution of an image of a fully unresolved double-star system, accurately resolving both its components. Notably, all DAMN reconstructions were performed without any prior knowledge of the optical setup, data preprocessing, or parameter estimation. Despite this, the obtained results surpass those of the established classical and deep learning methods. With sufficient computation resources, even further improvements can be achieved by incorporating more complex data simulations and larger network architectures. By taking this first step, our work lays a foundation for universal image reconstruction, entirely independent of optical settings, thus contributing to the advancement of image processing in various fields.

The device-agnostic paradigm presented in this study is not tied to a single specific neural network. It can be readily applied to any model and architecture that would be beneficial for a given application. Moreover, although the presented work focuses on super-resolution image reconstruction, the same framework can be readily applied to localization tasks, i.e., directly predicting emitter positions and intensities. Such an approach would be particularly beneficial for those single-molecule localization microscopy applications that require explicit information about emitter positions. Finally, the framework is not inherently restricted to optical imaging. The same principles can be applied to virtually any application that would benefit from improved generalization ability and hardware parameter independence.

During the preparation and review of the presented manuscript, we further developed the device-agnostic modeling framework toward dedicated applications in single-molecule fluorescence microscopy^66^ and material engineering for single-frame imaging of quantum dots^67^. In the former, we extend the analysis using our own experimentally acquired molecular dataset and provide a more detailed comparison with localization-based approaches. The latter applies the device-agnostic approach to our measured data of high-density In(Ga)As quantum dots and strain-induced dots in 2D monolayer WSe_2_. Both these studies build directly upon the general framework introduced here and focus on application-specific refinements and analysis.

## Methods

### Deep learning model

The central contribution of this work is not a specific trained network or a specialized architecture, but the device-agnostic modeling paradigm itself. This framework can be applied to any network with an arbitrary architecture, provided it has sufficient representational capacity and complexity. To emphasize and demonstrate this feature, we trained two distinct architectures, commonly used in deep-learning-based image reconstruction: a fully convolutional neural network, ConvNet (applied to simulated data and optical experiment), that preserves the input spatial dimensions, and a ResNet-based architecture incorporating residual connections and an inherent eightfold upsampling (applied to astronomy, aberrations, and biological samples). Both networks can directly process images of arbitrary size and aspect ratio without retraining.

The fully convolutional model comprises 35 hidden layers with 71 channels each and a final output layer with a single channel. Information flows through each hidden layer via trainable 7 × 7 filters that apply local convolutional operations, allowing the network to detect localized patterns. These transformations are followed by a LeakyReLU activation function with a 0.05 negative slope, which introduces a computationally efficient nonlinearity without causing the dying ReLU problem^68^. Since we normalize the input samples to unit sum before processing, the output layer utilizes a softmax activation function^69^ to preserve this condition and ensure non-negative values. To prevent overfitting, each hidden layer is paired with dropout regularization at a 0.01 rate, randomly setting a fraction of input units to zero during training^70^. This regularization encourages the network to learn robust features that do not rely on any specific neurons. Altogether, this model contains over 8 million trainable parameters and, after initialization, takes approximately 40 ms and 250 ms to process a 100 × 100 px and 500 × 500 px image, respectively, using an NVIDIA RTX A5000 GPU, without the need for retraining between applications.

The architecture of the upsampling ResNet-based model differs substantially from the dimension-preserving ConvNet. Its basic building block consists of two convolutional layers with 64 channels and a LeakyReLU activation (negative slope of 0.1). Each convolution is followed by batch normalization, which stabilizes the training by normalizing the mean and variance of activations across the batch. In addition, each block incorporates a residual connection, providing an additive shortcut to assist gradient propagation. Three such residual blocks are stacked to form a processing segment operating at a fixed spatial dimension. The full network comprises four of these segments, separated by bilinear interpolation layers that each double the spatial dimensions, resulting in an overall eightfold upsampling. The convolutional filter sizes within the four segments are 5 × 5, 7 × 7, 9 × 9, and 11 × 11 pixels, respectively. Owing to the progressive upsampling, the largest 11 × 11 filters correspond to an effective receptive field of approximately 1.5 pixels in the original input domain. The output layer produces a single-channel reconstruction and uses a softmax activation to ensure normalized, positive outputs. In total, the upsampling architecture contains more than 7 million trainable parameters and, after initialization, takes approximately 1.8 s and 3.5 s to process a 100 × 100 px and 500 × 500 px image, respectively, using an NVIDIA RTX A5000 GPU, and does not need to be retrained across applications.

The presented model architecture configurations result from extensive manual optimization across the device-agnostic regime. Each model was trained incrementally on approximately 3 million simulated low-resolution images, with a 25% validation set split. Incremental learning techniques gradually present the training data to the network. These techniques involve training the model using randomly generated data, which are continuously replaced with newly generated samples throughout the training process^55^. The level of network complexity and dataset size exceeds the conventional algorithms in the field of emitter reconstruction and localization. Nevertheless, the model remains relatively modest compared to modern large language models, allowing additional potential for improvement, including larger upsampling. Unfortunately, our ability to further increase model complexity is limited by the computational resources available to us.

The training of the DAMN model follows the backpropagation algorithm, which computes the gradient of the loss function with respect to each weight in the network using the chain rule. We use a mean squared error (MSE) loss, calculated between the predicted *I*_1_ and target *I*_2_ images and averaged over a batch of data samples. Given a set of two-dimensional image pairs, we calculate the loss as

1 $$\,{\mbox{MSE}}\,=\frac{1}{J}{\sum }_{j=1}^{J}{\sum }_{k=1}^{K}{\sum }_{l=1}^{L}{\left[{I}_{1}\left(j,k,l\right)-{I}_{2}\left(j,k,l\right)\right]}^{2},$$

where *J* is the batch size, *K* and *L* are the image dimensions, and *I*(*j*, *k*, *l*) is the intensity of the *j*-th image in the batch at the pixel position (*k*, *l*). The gradients obtained from this loss are used to iteratively update the weights with each batch of 128 training samples. To improve convergence towards the minimum loss, we implement the Adam optimizer^71^, which incorporates adaptive moment estimation. Adam updates the weights using both the first and second moments of the gradient, making the descent more efficient. A portion of the training data is reserved as a validation set to actively monitor the convergence using the mean absolute error (MAE) metric

2 $$\,{\mbox{MAE}}\,=\frac{1}{J}{\sum }_{j=1}^{J}{\sum }_{k=1}^{K}{\sum }_{l=1}^{L}| {I}_{1}\left(j,k,l\right)-{I}_{2}\left(j,k,l\right)| .$$

The metric provides an additional measure of model performance that is, unlike a loss function, not directly optimized during training. Additionally, for the upsampling model operating on the finer grid, the *I*_1_ and *I*_2_ images are first convolved with a small Gaussian filter to improve convergence during training, and the loss function includes a weak entropy regularization term to promote sparsity.

Any further details about the architecture designs and their training can be found in our GitHub and Zenodo repository^72^.

### Data simulation

A resolution-limited image can be characterized by its underlying optical parameters. In our case, these parameters include the shape and width of the point spread function, the background noise intensity, the emitter power representing the number of emitted photons, and the number of emitters present within a 50 × 50 pixels field of view of an image, which we term emitter concentration. Consequently, all possible resolution-limited images form a high-dimensional space. To implement the device-agnostic framework with a data-driven deep learning algorithm, the model must observe data samples covering the space during training. Therefore, the approach highly benefits from using simulated data pairs, as collecting a sufficient amount of experimentally acquired samples would be exceptionally time-consuming. Additionally, data simulation allows using incremental learning techniques (see Deep Learning Model), which are ideal for applications with large datasets.

To train the non-upsampling model, the following ranges of optical parameters were used for the data simulation: the average emitter power $\in [1,1{0}^{5}]$, the average shot noise $\in \left[1,100\right]$, the emitter concentration $\in \left[5,500\right]$, and the point spread function width $\in \,[1{0}^{-0.25},1{0}^{1.25}]$ px $\approx \left[0.5,17.75\right]$ px. These ranges represent extreme-to-extreme boundaries to demonstrate the device-agnostic abilities on a difficult, large-scale generalization task.

Training of the upsampling model is significantly more computationally expensive. To alleviate the training process on our limited resources, we chose to slightly narrow the ranges, disregarding the pathological and near-perfect cases. As such, the following ranges were employed: the average emitter power $\in \left[1,1{0}^{4}\right]$, the average noise $\in \left[1,100\right]$, the emitter concentration $\in \left[1,100\right]$, and the point spread function width $\in \left[1{0}^{0.1},1{0}^{1}\right]$ px $\approx \left[1.25,10.0\right]$ px. Nonetheless, these ranges still cover a broader range of optical conditions than those typically encountered in practical application.

The generation process of a single simulated data sample follows these steps. First, we generate the emitter concentration, followed by assigning each emitter its power and pixel position in the image. Next, we perform a convolution with the point spread function to simulate the effects of finite resolution. For the data samples utilized by the upsampling model training, we now sum each distinct 8 × 8 pixel region. Subsequently, shot noise is added to each pixel of the image. With knowledge of the average shot noise intensity and emitter power, we can calculate the signal-to-noise ratio of the generated sample as $\,{{\mbox{SNR}}}=\frac{{{\mbox{average}}} \,{{\rm{emitter}}} \, {{\rm{power}}}}{{{\mbox{average}}} \,{{\rm{shot}}}\, {{\rm{noise}}}\, {{\rm{intensity}}}}$. Alternatively, we can calculate the peak-to-noise ratio, $\,{{\mbox{PNR}}}=\frac{{{\mbox{peak}}}\, {{\rm{emitter}}}\, {{\rm{intensity}}}}{{{\mbox{average}}} \,{{\rm{shot}}} \,{{\rm{noise}}}\, {{\rm{intensity}}}}$, using the maximum value of the convolved emitter intensity.

To perform the convolution, we generate the convolution matrix of the point spread function using two distinct parameters—shape and width. The shape parameter distinguishes between an Airy pattern *A*, simulating low numerical aperture scenarios, and a two-dimensional Gaussian pattern *G*, typically used for cases with higher numerical aperture values^73^. The inclusion of both distinct shapes is important for unlocking the high generalization ability. When we originally trained only on one of the shapes, the generalization of the resulting model was inferior compared to that of the model trained on both shapes. Additionally, half of the generated point spread function profiles account for asymmetries by applying an additional squeezing along either the horizontal, vertical, diagonal, or anti-diagonal axis. The convolution matrix is normalized to a unit sum to conserve the emitter power. The pattern functions before squeezing can be expressed as

3 $$A(r,\sigma ) \sim {\left[\frac{{{\mbox{J}}}_{1}(\frac{2r}{\sigma })}{\frac{r}{\sigma }}\right]}^{2},$$

4 $$G(r,\sigma ) \sim \,{\mbox{exp}}\,\left(-\frac{{r}^{2}}{{\sigma }^{2}}\right),$$

where *r* is the distance from the center, and *J*_1_ is the first-order Bessel function of the first kind. The variable *σ* is the width parameter, which dictates the full width at half maximum of the point spread function as $2\sigma \sqrt{\ln 2}$.

It is worth noting that the simulation process can be further enhanced by incorporating additional features and parameters, such as different noise types and optical aberrations^74,75^. For this showcase study, we opted to simplify the simulation to demonstrate the strong generalization capability stemming even from the simplified data and to reduce the computational demand during training. Despite this simplification, the DAMN model achieves excellent results across all tested use cases and demonstrates significant generalization ability beyond the expected regime, as shown in Fig. 2d, and by the already high robustness to optical aberrations, as characterized in Supplementary Fig. 2 and its dedicated section.

Any further details about the data simulation process can be found in our GitHub and Zenodo repository^72^.

### Reporting summary

Further information on research design is available in the Nature Portfolio Reporting Summary linked to this article.

## Supplementary information

Supplementary Information

Reporting Summary

Transparent Peer Review file

## Data Availability

The data used in this study are publicly available in our GitHub and Zenodo repository^72^.

## Code availability

The code supporting the results of this study are publicly available in our GitHub and Zenodo repository^72^.
