# Supplementary material for: From stars to molecules: AI guided device-agnostic super-resolution imaging
Source: Nat Commun. 2026 Jul 16;17:8705. doi: 10.1038/s41467-026-75584-7 (PMC13490602; doi:10.1038/s41467-026-75584-7)
Supplement: Supplementary file 1 [file 41467_2026_75584_MOESM1_ESM.pdf]

Supplementary Information for "From Stars to Molecules: AI Guided Device-Agnostic Super-Resolution Imaging"

Dominik Vašinka,[1,*] Filip Juráň,[1] Jaromír Běhal,[1] and Miroslav Ježek[1,*]

[1]*Department of Optics, Faculty of Science, Palacký University, 17. listopadu 12, 77900 Olomouc, Czechia*

Experiment

The optical setup, which provides resolution-limited experimental images with their corresponding ground truths, consists of three segments, see Extended Data Fig. 1. The first segment prepares the illumination of the digital micromirror device (DMD). An incoherent broadband light, produced by a laser-induced fluorescence light source (Crytur MonaLIGHT B01), is coupled to a 4 mm diameter light guide. The emerging light is collimated by a microscope objective MO_s (OLYMPUS 20x/0.4). An iris diaphragm D is positioned proximal to the objective. This diaphragm is re-imaged onto the DMD (Texas Instruments DLP LightCrafter 6500) by a telescope consisting of two achromatic doublets, L_{s1} and L_{s2}, with focal lengths of 50 mm and 150 mm, respectively. The telescope improves the illumination homogeneity, and re-imaging the diaphragm reduces reflections from the passive parts of the DMD.

In the second setup segment, point-like emitters with the targeted spatial distribution are prepared. By addressing the DMD, we impose a ground-truth mask with each pixel comprising a 5×5 micromirror grid with a 7.6 μm micromirror pitch. As we address only the central micromirror of the grid, the closest distance between two emitting sources is 38 μm in the DMD plane, see Extended Data Fig. 1. This mask is re-imaged into the front focal plane of MO_p by a preparation telescope consisting of a 200 mm focal-length achromatic doublet L_p and a high-resolution microscope objective MO_p (OLYMPUS 100x/0.9). As the point-like emitters, of the approximate 310 nm full width at half maximum and 340 nm minimal distance, are created in this plane, we term it the emitter plane.

Unlike the first two segments, which prepare the emitters and provide partial control and complete knowledge over their spatial distributions, the final segment represents an imaging setup with optical parameters unknown to the DAMN model. A low-resolution microscope objective MO_I (OLYMPUS 10x/0.25) projects the emitter plane onto a CMOS camera (ZWO-ASI 178MM, 2.4 μm square pixels) with a mounted spectral filter (central wavelength of 532 nm with 10 nm spectral width). The MO_I resolution is approximately 1.3 μm, regarding the Rayleigh limit in the emitter plane. In comparison, the 340 nm minimal emitter distance is almost 4 times below this resolution limit. The emitter plane is imaged with an approximate 230× magnification to match the major part of the camera chip. The exposure time for each measurement is set to 5.7 seconds to utilize the majority of the 14-bit dynamic range of the camera while avoiding its saturation. The captured image is downscaled by a factor of 32 to match the 50×50 ground-truth mask size to enable metric evaluations. Using this imaging setup, we collected the experimental dataset of resolution-limited camera images and their corresponding ground-truth masks for various emitter concentrations, see Fig. 4 a of the main text. Additionally, we measured a high-SNR calibration set for precise estimation of the point spread function, which we provided only to both variants of the Richardson-Lucy algorithm and the Deep-STORM models. Besides the concentration ranging from 50 to 500 emitters, the optical parameters in the collected dataset exhibit the following estimated values: SNR = 2300, average noise intensity = 10, and an Airy-shaped kernel with $\sigma = 2.05$ px, equivalent to the 3.4 px full width at half maximum. The pixel-based Rayleigh resolution limit of our imaging system, given by the radius R of the first dark ring in the Airy pattern, is approximately 3.9 px, see Fig. 4 b of the main text.

Aberration analysis

Since the DAMN model is trained on simulated data using either a Gaussian or an Airy point spread function, we evaluated its performance on images containing simulated optical aberrations. We generated vectorial point spread functions using a high-numerical-aperture Debye–Wolf diffraction model [1]. Simulations assumed a wavelength of $\lambda = 532$ nm, a numerical aperture of 1.4 (modified for the numerical-aperture dependence analysis), and a homogeneous immersion medium with a refractive index of 1.5. Circularly polarized illumination was assumed, and the vectorial pupil field included the standard $\sqrt{\cos\theta}$ apodization. Realistic aberrations of increasing strength were modeled using Zernike polynomials, specifically coma, astigmatism (both in- and out-of-focus), defocus, and spherical aberration. The aberration strength, referring to the value of the Zernike coefficient, is given in units of wavelength. For each aberration type and strength, we simulated 100 images with SNR = 10^3, average noise intensity of 10, and 20 emitters. The results, including the dependence of reconstruction performance on numerical aperture, are shown in Extended Data Fig. 2. Any further details about the aberration simulation process can be found in our GitHub repos-

* vasinka@optics.upol.cz, jezek@optics.upol.cz

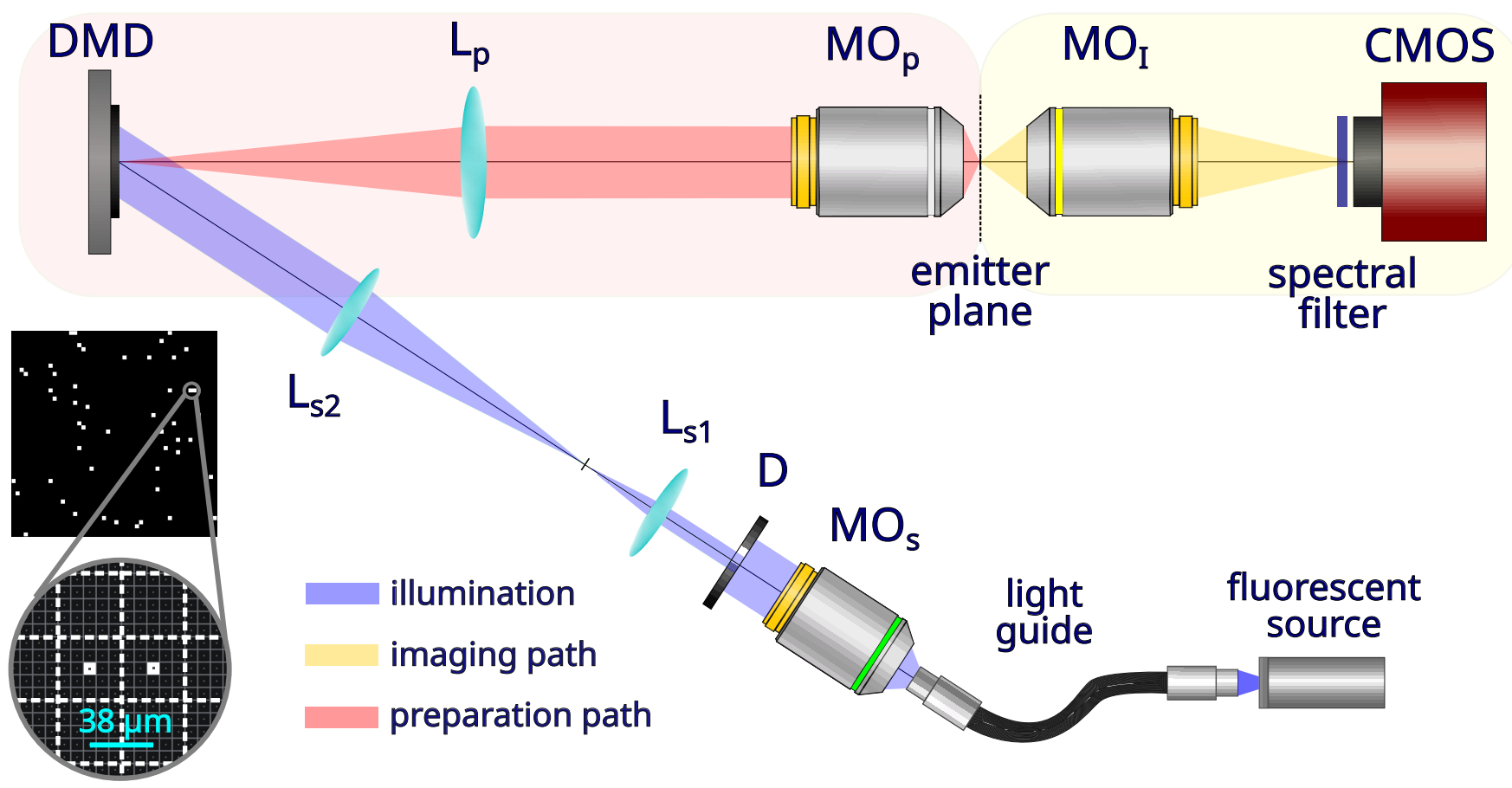

Supplementary Fig. 1. A detailed visualization of the optical setup with partial control and complete knowledge over the ground truth of emitters. In the illumination path (blue), we generate an incoherent light collimated by a microscopic objective $\mathrm{MO_s}$. The subsequent iris diaphragm D and two achromatic doublets, $\mathrm{L_{s1}}$ and $\mathrm{L_{s2}}$, improve the illumination homogeneity and reduce undesired reflections from the passive digital micromirror device DMD parts. In the preparation path (red), the DMD-induced ground-truth mask is re-imaged by an achromatic doublet $\mathrm{L_p}$ and a high-resolution microscope objective $\mathrm{MO_p}$, creating point-like emitters in the emitter plane. Lastly, this plane is projected by a low-resolution microscope objective $\mathrm{MO_I}$ onto a CMOS camera with a mounted spectral filter in the imaging path (yellow).

itory [2]. The reported metric represents the relative change in mean absolute error compared to the baseline for non-aberrated point spread functions.

As illustrated in Extended Data Fig. 2 (a), the DAMN model demonstrates strong robustness against all aberration types. The relative error increase remains below 2% across most realistic values of aberration strength and numerical aperture. Notably, gradually decreasing the numerical aperture shifts the intensity profile from a Gaussian to an Airy pattern while simultaneously broadening the effective width of the point spread function. Based on the results presented in Fig. 2 of the main text, the slight increase in relative error is primarily due to the increased width, which is approaching the field-of-view limits. Overall, these results indicate that the DAMN model reliably reconstructs even strongly aberrated images, with only a few percent increase in the mean absolute error metric.

Moreover, Extended Data Fig. 2 (b) shows the point spread functions for each aberration type at a strength of 0.2λ, alongside the non-aberrated baseline and a point spread function with a numerical aperture of 0.2. These cases represent severely distorted point spread function profiles, which are rarely encountered in standard imaging scenarios. Nonetheless, the reconstruction errors are only marginally affected, and the trained model remains highly effective even for images with aberrations.

Finally, the reconstruction quality achieved in the biological (tubulin and nuclear pore complexes) and astronomical (double stars) applications further supports these findings. These experimentally acquired images inherently suffer from non-ideal imaging conditions and unknown aberrations. Nevertheless, the DAMN model reconstructs them with high fidelity, as discussed in the respective sections. Furthermore, the training data simulation could be extended to incorporate realistic aberrations, which would further enhance robustness to such imperfections at the cost of increased training process complexity. We deliberately refrained from doing so to demonstrate the strong generalization capability of the device-agnostic modeling, even when training on simplified simulation data.

Richardson-Lucy Algorithms

The Richardson-Lucy algorithm is an iterative method used to restore an image blurred by a known point spread function [3, 4]. This sophisticated and highly flexible algorithm is derived from the Bayesian probability theory with a flat prior. It maximizes the posterior probability that the observed image results from the estimated image convolved with the given point spread function. Assuming a multinomial distribution of detection events, the Richardson-Lucy is equivalent to the expectation-maximization algorithm for likelihood maximization in positive linear inverse problems [5, 6]. The quality of the reconstructed image relies heavily on accurate knowledge of the point spread function; discrepancies can lead to artifacts and inaccurate reconstructions. Since we simulate the testing data, the Richardson-Lucy algorithm can use the precise point spread function, making it an excellent method for providing a classical baseline for evaluating the DAMN model.

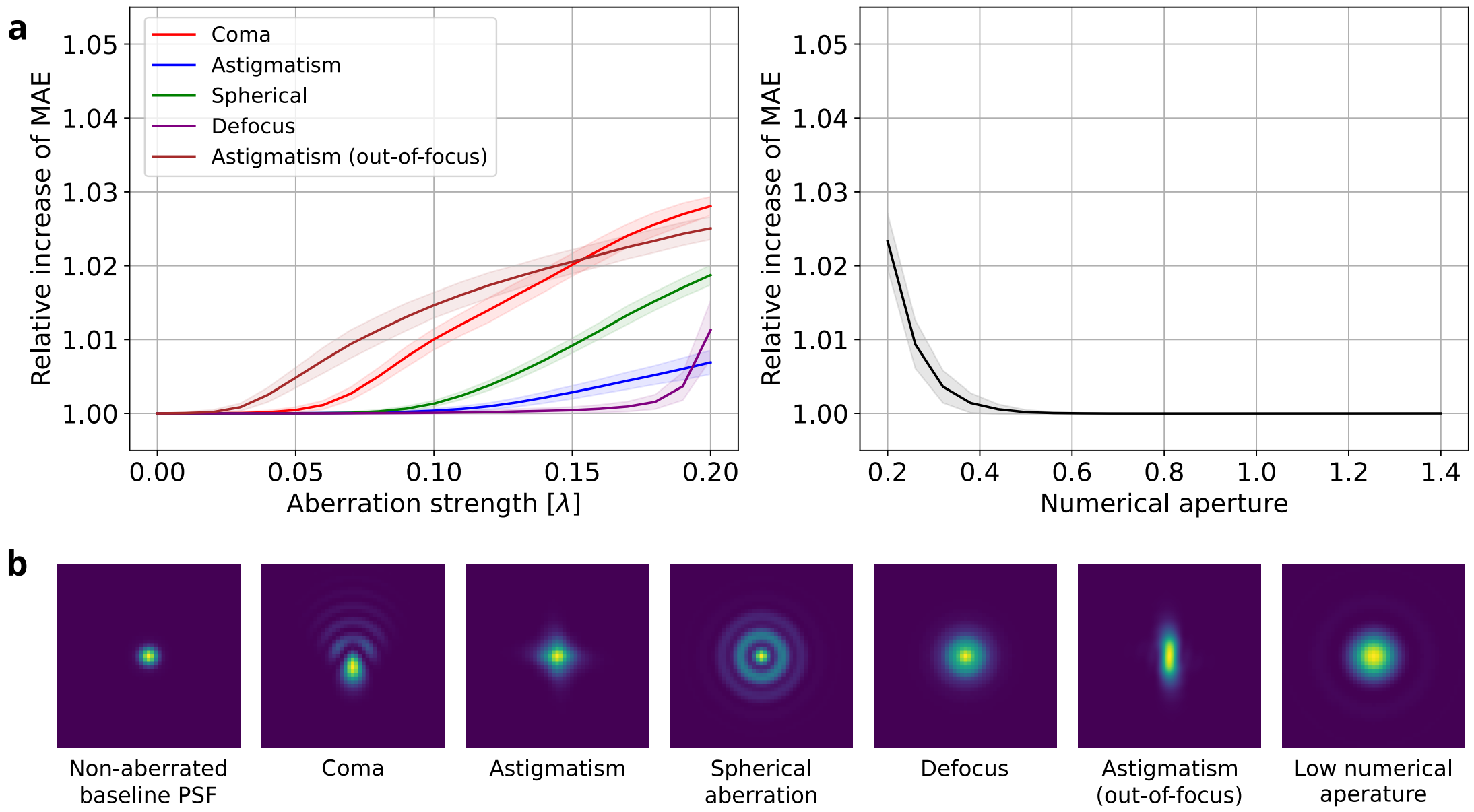

Supplementary Fig. 2. The aberration effects on the DAMN reconstruction performance. **(a)** The relative increase of mean absolute error (MAE) due to the numerical aperture (black) and aberration strength values for coma (red), astigmatism (blue), spherical aberration (green), defocus (purple), and out-of-focus astigmatism (brown). The presented results were evaluated relative to the baseline errors obtained for the non-aberrated point spread function (PSF). **(b)** Visualization of the distorted point spread functions for each aberration type with 0.2λ strength, alongside the non-aberrated baseline and 0.2 numerical aperture.

Moreover, the Richardson–Lucy algorithm can be viewed as an iterative maximum-likelihood method for Poisson observations. Under ideal model assumptions, if iterated to the maximum-likelihood solution and if the usual regularity conditions hold, its estimator may be asymptotically efficient, i.e., approach the classical Cramér–Rao bound in a large-data limit. This is the main reason we chose Richardson-Lucy as one of the benchmarking methods in our work.

In addition to the point spread function P, the iterative algorithm requires an initial guess for the reconstruction, $I^{(0)}$. A common practice is to set this guess as the observed resolution-limited image I_{blurred} or to start with a uniform image. We explored both approaches and chose to set $I^{(0)} = I_{\text{blurred}}$, as there was a negligible difference in accuracy and computational speed. Then, an iterative procedure updates the estimation at the $k+1$ step

$$I^{(k+1)} = I^{(k)} \cdot \left(\frac{I_{\text{blurred}}}{I^{(k)} \circledast P} \circledast P^* \right), \tag{1}$$

where $\circledast$ is convolution, and P^* denotes the flipped point spread function. Both the initial guess $I^{(0)}$ and the point spread function P need to be normalized to a unit sum. We implement two stopping criteria for the algorithm: either the mean absolute error between successive iterations becomes smaller than 10^{-10} per pixel, or the number of iterations exceeds 10^6. The first criterion is typically met within 10^4-10^5 iterations in the majority of cases.

Moreover, we evaluated a Richardson-Lucy algorithm with total variation regularization [7]. This variant is designed to suppress unstable oscillations and mitigate noise-amplification artifacts inherent to the classical formulation. In each iteration, the update step includes an additional division by the regularization term

$$1 - \lambda_{TV} \nabla \cdot \left(\frac{\nabla I^{(k)}}{|\nabla I^{(k)}|} \right), \tag{2}$$

where $\lambda_{TV} = 2 \times 10^{-3}$ denotes the regularization strength, ∇ is the gradient operator, and $\nabla\cdot$ is the divergence operator. We tested several values of the regularization strength and chose to proceed with the original default value. The stopping criteria were kept identical to those of the non-regularized algorithm.

Deep-STORM

To benchmark our results also against a deep learning method, we implemented Deep-STORM [8], which produces super-resolution images from a dataset of stochastically blinking emitters. Deep-STORM is a convolutional neural network that reconstructs a high-resolution version of individual low-resolution frames. Therefore, it is directly comparable to our DAMN model. However, Deep-STORM is a device-specific method trained on simulated or experimentally acquired images with fixed imaging parameters, such as camera specification,

point-spread-function profile, signal-to-noise ratio, and emitter density.

To compare its reconstructions in Fig. 2 and Fig. 4 of the main text, we trained numerous device-specific Deep-STORM models, one for each point in the graphs. These models were trained using simulated data with precise signal-to-noise ratios, concentrations, noise distributions, and point spread functions (widths and shapes), matching the targeted testing conditions. The model used to reconstruct the tubulin structures in Fig. 6 of the main text was trained by the authors and is available in their GitHub repository. This repository also includes the necessary code that we used to train all the remaining models. All training parameters, such as the loss function and the number of epochs, were kept intact as recommended by the authors.

Additionally, the Deep-STORM architecture inherently provides an eightfold increase in the reconstructed spatial dimensions. While we could deactivate this upsampling, it would significantly bottleneck the information flow throughout the architecture. Therefore, we chose to retain it to avoid disrupting the model capacity and complexity. Instead, we applied 8×8 downscaling to the reconstructed images in places where the metric evaluation required non-upsampled image sizes (Fig. 2 and 4 of the main text).

[1] Wolf, E. Electromagnetic diffraction in optical systems - I. An integral representation of the image field. *Proceedings of the Royal Society of London. Series A. Mathematical and Physical Sciences* **253**, 349–357 (1959).

[2] Vašinka, D. From Stars to Molecules: AI Guided Device-Agnostic Super-Resolution Imaging. *Github repository.* 10.5281/zenodo.20584806 (2026).

[3] Richardson, W. H. Bayesian-based iterative method of image restoration. *Journal of the Optical Society of America* **62**, 55 (1972).

[4] Lucy, L. B. An iterative technique for the rectification of observed distributions. *The Astronomical Journal* **79**, 745 (1974).

[5] Dempster, A. P., Laird, N. M. & Rubin, D. B. Maximum likelihood from incomplete data via the em algorithm. *Journal of the Royal Statistical Society: Series B (Methodological)* **39**, 1–22 (1977).

[6] Vardi, Y. & Lee, D. From image deblurring to optimal investments: Maximum likelihood solutions for positive linear inverse problems. *Journal of the Royal Statistical Society: Series B (Methodological)* **55**, 569–612 (1993).

[7] Dey, N. *et al.* Richardson–Lucy algorithm with total variation regularization for 3D confocal microscope deconvolution. *Microscopy Research and Technique* **69**, 260–266 (2006).

[8] Nehme, E., Weiss, L. E., Michaeli, T. & Shechtman, Y. Deep-storm: super-resolution single-molecule microscopy by deep learning. *Optica* **5**, 458 (2018).
