# Supplementary material for: From stars to molecules: AI guided device-agnostic super-resolution imaging
Source: Nat Commun. 2026 Jul 16;17:8705. doi: 10.1038/s41467-026-75584-7 (PMC13490602; doi:10.1038/s41467-026-75584-7)
Supplement: Supplementary file 2 [file 41467_2026_75584_MOESM2_ESM.pdf]

nature portfolio

Corresponding author(s): Dominik Vašinka, Miroslav Ježek

Last updated by author(s): Mar 2, 2026

Reporting Summary

Nature Portfolio wishes to improve the reproducibility of the work that we publish. This form provides structure for consistency and transparency in reporting. For further information on Nature Portfolio policies, see our Editorial Policies and the Editorial Policy Checklist.

Statistics

For all statistical analyses, confirm that the following items are present in the figure legend, table legend, main text, or Methods section.

n/a	Confirmed	
☒	☐	The exact sample size (n) for each experimental group/condition, given as a discrete number and unit of measurement
☐	☒	A statement on whether measurements were taken from distinct samples or whether the same sample was measured repeatedly
☒	☐	The statistical test(s) used AND whether they are one- or two-sided<br>*Only common tests should be described solely by name; describe more complex techniques in the Methods section.*
☒	☐	A description of all covariates tested
☒	☐	A description of any assumptions or corrections, such as tests of normality and adjustment for multiple comparisons
☐	☒	A full description of the statistical parameters including central tendency (e.g. means) or other basic estimates (e.g. regression coefficient) AND variation (e.g. standard deviation) or associated estimates of uncertainty (e.g. confidence intervals)
☒	☐	For null hypothesis testing, the test statistic (e.g. F, t, r) with confidence intervals, effect sizes, degrees of freedom and P value noted<br>*Give P values as exact values whenever suitable.*
☐	☒	For Bayesian analysis, information on the choice of priors and Markov chain Monte Carlo settings
☒	☐	For hierarchical and complex designs, identification of the appropriate level for tests and full reporting of outcomes
☒	☐	Estimates of effect sizes (e.g. Cohen's d, Pearson's r), indicating how they were calculated

Our web collection on statistics for biologists contains articles on many of the points above.

Software and code

Policy information about availability of computer code

Data collection	All simulated data were generated using our custom code, which is available at our GitHub repository: https://github.com/VasinkaD/DAMN.
Data analysis	All data were evaluated using our custom code, which is available at our GitHub repository: https://github.com/VasinkaD/DAMN.

For manuscripts utilizing custom algorithms or software that are central to the research but not yet described in published literature, software must be made available to editors and reviewers. We strongly encourage code deposition in a community repository (e.g. GitHub). See the Nature Portfolio guidelines for submitting code & software for further information.

Data

Policy information about availability of data

All manuscripts must include a data availability statement. This statement should provide the following information, where applicable:

- Accession codes, unique identifiers, or web links for publicly available datasets
- A description of any restrictions on data availability
- For clinical datasets or third party data, please ensure that the statement adheres to our policy

The measured data and codes for generating the simulated data are available at our GitHub repository: https://github.com/VasinkaD/DAMN.
The binary star, tubulin dataset, and nuclear pore complexes data relate to the following studies, respectively:
Chambers, K. C., Magnier, E. A., Metcalfe, N. et al. The Pan-STARRS1 Surveys (2016), arXiv:1612.05560;

Sage, D. et al. Quantitative evaluation of software packages for single-molecule localization microscopy, Nature Methods 12, 717–724 (2015);
Thevathasan, J. V. et al. Nuclear pores as versatile reference standards for quantitative superresolution microscopy. Nature Methods 16, 1045–1053 (2019).

Research involving human participants, their data, or biological material

Policy information about studies with human participants or human data. See also policy information about sex, gender (identity/presentation), and sexual orientation and race, ethnicity and racism.

Reporting on sex and gender	Research does not involve human participants.
Reporting on race, ethnicity, or other socially relevant groupings	Research does not involve human participants.
Population characteristics	Research does not involve human participants.
Recruitment	Research does not involve human participants.
Ethics oversight	Research does not involve human participants.

Note that full information on the approval of the study protocol must also be provided in the manuscript.

Field-specific reporting

Please select the one below that is the best fit for your research. If you are not sure, read the appropriate sections before making your selection.

☒ Life sciences ☐ Behavioural & social sciences ☐ Ecological, evolutionary & environmental sciences

For a reference copy of the document with all sections, see nature.com/documents/nr-reporting-summary-flat.pdf

Life sciences study design

All studies must disclose on these points even when the disclosure is negative.

Sample size	No predetermination of sample size was performed. The incremental learning technique gradually generated new training samples until the convergence of the model´s metric. The measured data from our optical setup were collected in amount sufficient for characterizing the statistical behaviour under each experimental condition.
Data exclusions	No data were excluded.
Replication	The evaluation on simulated data produce results virtually independent of a random seed choice. Measured dataset from our experiment contains a thousand samples per each graph point for extensive statistical evaluation. All attempts at replicating the results of the study were successful.
Randomization	No randomization was performed. Randomization is not relevant nor applicable to the study.
Blinding	No blinding was performed. Blinding is not relevant nor applicable to the study.

Reporting for specific materials, systems and methods

We require information from authors about some types of materials, experimental systems and methods used in many studies. Here, indicate whether each material, system or method listed is relevant to your study. If you are not sure if a list item applies to your research, read the appropriate section before selecting a response.

Materials & experimental systems

n/a	Involved in the study	
☒	☐	Antibodies
☒	☐	Eukaryotic cell lines
☒	☐	Palaeontology and archaeology
☒	☐	Animals and other organisms
☒	☐	Clinical data
☒	☐	Dual use research of concern
☒	☐	Plants

Methods

n/a	Involved in the study	
☒	☐	ChIP-seq
☒	☐	Flow cytometry
☒	☐	MRI-based neuroimaging

Plants

Seed stocks	Research does not involve the study of plants.
Novel plant genotypes	Research does not involve the study of plants.
Authentication	Research does not involve the study of plants.
