# Supplementary material for: From stars to molecules: AI guided device-agnostic super-resolution imaging
Source: Nat Commun. 2026 Jul 16;17:8705. doi: 10.1038/s41467-026-75584-7 (PMC13490602; doi:10.1038/s41467-026-75584-7)
Supplement: Supplementary file 3 [file 41467_2026_75584_MOESM3_ESM.pdf]

nature portfolio

Peer Review File

From Stars to Molecules: AI Guided Device-Agnostic Super-Resolution Imaging

Corresponding Author: Mr Dominik Vasinka

Parts of this Peer Review File have been redacted as indicated to remove third-party material.

This file contains all reviewer reports in order by version, followed by all author rebuttals in order by version.

Attachments originally included by the reviewers as part of their assessment can be found at the end of this file.

Version 0:

Reviewer comments:

Reviewer #1

(Remarks to the Author)
In the manuscript, the authors introduce a deep-learning calibration-free method for super-resolution imaging of point-like emitters from single diffraction-limited frames called Device-Agnostic Modeling Network (DAMN). The model is trained on synthetic data covering a range of Gaussian PSFs, noise levels, and emitter densities, and is validated on both microscopy and astronomy images.

Although the study is well conducted and the results are promising, several important points require clarification before I can recommend the manuscript for publication.

Major Concerns

1. Extensive overlap with a concurrent preprint. There are serious concerns regarding the substantial overlap between the DAMN manuscript (From Stars to Molecules: AI Guided Device-Agnostic Super-Resolution Imaging) and the recent preprint Calibration-free single-frame super-resolution fluorescence microscopy (CFCNN) by Dostálová et al., which shares two authors and much of the core content.
Both works propose a calibration-free, deep-learning approach trained on synthetic data to reconstruct super-resolved images from single diffraction-limited frames. Key elements are nearly identical:
- Synthetic data generation with Gaussian PSF shapes, emitter densities, and noise levels.
- Convolutional network architectures with upsampling, LeakyReLU, and dropout.
- Benchmarking against Richardson–Lucy deconvolution.
Aside from minor architectural changes and the addition of astronomy data, there appear to be no significant technical distinctions. The DAMN manuscript must therefore: cite and discuss the concurrent preprint explicitly. State precisely what new capability, performance gain, or application scope distinguishes DAMN from the CFCNN approach reported in the preprint.

2. Incomplete benchmarking against deep learning-based deconvolution algorithms. In figure 2, the manuscript only compares DAMN to Richardson–Lucy deconvolution, which is a classical method. It would be valuable to include comparisons with other deep-learning-based deconvolution algorithms (e.g., ESRGAN, CARE, RCAN, or SRResNet), or with more recent approaches based on Richardson-Lucy (RLN) (Li, Y., et al. Nat Methods 19, 1427–1437 (2022)) or Wiener deconvolution, to better position the method relative to the current state of the art.

3. Missing quantitative metrics vs Deep-STORM. Only qualitative side-by-side images are shown. It would strengthen the analysis to include quantitative resolution metrics, such as the Fourier Ring Correlation (FRC), or similar measures, preferably mapped across the field of view (Culley, S. et al., Nat Methods 15, 263–266 (2018)), to validate the claimed performance advantage.

4. Reproducibility: training code and data. The public GitHub repository currently contains only figure-generation scripts. For

reproducibility, the community needs access to the training code (including network definition, loss functions, and hyperparameters); either the full or a representative portion of the synthetic training and validation sets, or the scripts and parameters required to regenerate them.

Minor Concerns

1. Clarify whether the astronomical demonstration uses the same trained network as in the microscopy experiments, or whether a fine-tuned variant was applied.

2. Considering that the training data were generated using Gaussian-modeled PSFs, it would be helpful to provide examples of typical microscopy conditions, such as recommended minimum pixel size (to satisfy the Nyquist criterion) and/or objective magnifications that correspond to the synthetic training data. This would help users avoid oversampling when applying the model.

3. The authors mention that the simulated data represent emitted photons as "emitter power." It would be useful to show, perhaps in the supplementary materials, how this value and the average noise level were selected. Currently, the criteria for choosing the range of optical parameters used in the data simulation are unclear. The authors could support their choices by citing relevant references or showing examples of real data that match those conditions.

4. It would strengthen the manuscript to include results on biological samples beyond microtubules, such as nuclear pore complexes, whose size and spacing are well characterized. This would better demonstrate the performance of DAMN in practical scenarios and enable comparison with other super-resolution methods.

5. It would also be helpful to test the model using a mixture of fluorescent polystyrene beads of different known sizes. This could help assess the accuracy of the model's estimations and its ability to resolve heterogeneous structures.

6. The manuscript contains several general claims about resolution improvement, such as in line 303: "leading to an even more prominent improvement in the resolution," and in line 385: "The analysis results undeniably show the superior performance of our approach, outperforming the Richardson-Lucy algorithm by orders of magnitude in terms of both reconstruction accuracy and speed." It is important to provide quantitative values to support these claims, along with the parameters used to define or compute resolution.

7. The limitations of the model should be discussed, for instance, in cases where emitters are closely spaced or overlapping. This is an important consideration in super-resolution imaging of dense samples.

8. In line 584, the authors compare the computational performance of Richardson-Lucy with DAMN, but it is not clear whether the Richardson-Lucy implementation used a GPU. Including results from a GPU-based Richardson-Lucy implementation would offer a fairer comparison with the DAMN model running on a GPU.

Reviewer #2

(Remarks to the Author)
The manuscript bij Vašinka et al describes a new deep-learning methods for generalized single-emitter imaging. The method is described to be capable of resolving a wide variety of conditions (e.g. scales from microscope to astronomy) with better-than-state-of-the-art accuracy. The method is well introduced, researched, described, and training data and computational methods are well described and probably reproducible. I'd like to note that I have a background in single-molecule microscopy and thus focused my review on this field.

However, I unfortunately do not, as of yet, agree with the researchers claims they made – i.e. they are either overpromising or under-delivering. These are my key notes:
- The authors claim to have a method that works 'without the need for calibration measurements' (e.g. lines 144-146). However, the only training data they provide is in the form of PSFs that are shaped like 2d, symmetrical Gaussians, or 2d, symmetrical Airy discs, with varying widths and intensities. This leaves out the majority of the work performed in single-molecule microscopy reconstruction over the past decade, which has been on non-ideal PSFs. There is no consideration of non-ideal PSFs, i.e. optical aberrations (Zernike coefficients), defocus, blurred emitters, refractive index mismatches, etc etc. Without these considerations, I do not believe that this method can be stated to be 'universally applicable' – it can only be applied in very specific, (near-)ideal conditions that are almost never realized in single-molecule microscopy.
- The focus on difference between Gaussian and Airy PSFs is redundant in my opinion, since they are nearly identical, especially given that the outer rings are lost in noise.
- I strongly disagree with their statement that image reconstruction is 'the more universal approach' (line 86). In SMLM, mostly images are indeed reconstructed, but if one obtains a list of underlying emitter positions, creating an image is trivial. I.e. there is no information lost by obtaining emitter positions from a model, but there is information lost by only looking at image reconstruction. Emitter position (authors call this parameter estimation) is also the harder problem to solve.
- Along these same lines, the focus on image reconstruction limits their method to a certain pixel size – interpreting figure b, it's around 60-70 nm accuracy in SMLM. This is an order of magnitude worse than state-of-the-art localization methods.
- Figure 4 shows the key SMLM showcase of accuracy, but this is a very simplified method, with uniformily identical PSFs, uniformily identical intensities, and (in my opinion most importantly), structured information about the emitter position – since the 'emitter position' is determined by the DMD pixel, there can never be 2 emitters closer spaced than ~65 nm, which strongly hinders all interpretation of the method.

- In the methods somewhere (I believe), the authors discuss upsampling to resolve this issues to a certain extent, but do not show this at all.
- The SOSplugin reconstruction of the figure 6 dataset (b) is flawed in multiple ways. First of all, the SMLM challenge provides GT localizations, which can be used as reference; secondly, visualization of single molecules as scatter plots is a very flawed method – should be considering the loc. Uncertainty and visualization via upsampled gaussians, etc.
- Finally, combining methods from astronomy and SMLM is not novel – in fact, one of the first SMLM reconstruction methods has made this connection ~15 years ago – and this paper is not mentioned here (Holden, Uphoff, Kapanidis, Nat Meth, 2011).
Other, more minor points:
- I feel the authors overstate the problems which have already been addressed in earlier work, especially given the issue of obtaining in situ PSFs from your set-up. Many methods exist to directly extract this from a single in situ dataset.
- Figure 2b and 2c, I believe, look at the same core problem. With increased PSF width, there is a problem of overlapping emitters if the emitter density is kept stable, which results in the sharp increase in error of the DAMN model.
- I'm unsure why the Richardson-Lucy deconvolution isn't applied on the fig6 data as well, seeing as it's the author's go-to comparison method up to that point.

Reviewer #3

(Remarks to the Author)
The authors introduce a deep learning (DL) model for super-resolution imaging and demonstrate its applicability on both microscopy and astronomical data. They also describe an optical setup designed to acquire pairs of low and high resolution images for validation. The authors claim a device-agnostic DL model. However, there are serious concerns regarding methodological novelty, benchmarking and validation, and the choice of the application domain.

Firstly, the proposed model is essentially a straightforward, fully data-driven architecture with incremental learning, presenting limited methodological novelty for computational imaging. The literature review omits state-of-the-art DL approaches, including plug-and-play, deep unrolling (see Kamilov et al., 2022, arXiv:2203.17061), and diffusion-based reconstruction approaches. These approaches typically generalise more effectively than purely data-driven DL models and should be discussed.

Secondly, benchmarking solely against the classical Richardson–Lucy (RL) algorithm (Richardson 1972; Lucy 1973) is not sufficiently motivated. The authors should clarify why RL is considered "state-of-the-art" in this context rather than comparing against established and recent approaches used in the relevant imaging domains. Moreover, in the comparison with RL, strict stopping criteria were adopted, including extremely small tolerance (10^-10) and a very large iteration number (10^6), which raises concerns about the fairness of the comparison and undermines claims of computational efficiency. On a further note, given the simple nature of RL iteration structure, it could have been easily deployed on GPU similarly to the proposed DL model. For the real single-molecule microscopy data, although Deep-STORM is considered in the comparison, performance gains are marginal and computational cost is not discussed. As for the real astronomical illustration, the analysis of the results is not supported with a rigorous validation (including the accuracy of the position and flux of the resolved point sources), thus limiting the interpretability of the results.

Finally, the choice of astronomy as a validation domain is not adequate. Given the current landscape of modern telescopes, astronomical imaging often involves extended sources, and compact sources with complex morphology. It also requires algorithms that scale efficiently to large surveys, making the current approach difficult to generalise, thus severally limiting its impact in this domain.

Version 1:

Reviewer comments:

Reviewer #1

(Remarks to the Author)
In their revised version of the manuscript, the authors have carefully addressed my questions and concerns (both major and minor). Not only my comments, but also those of the other reviewers, have been addressed thoroughly.

The paper has improved quite a bit, and all issues have been resolved to my full satisfaction. Additionally, the GitHub repository has improved significantly, including the Zenodo additions.

I therefore recommend acceptance of this manuscript for publication.

(Remarks on code availability)
Unfortunately, I could not install and run the code myself due to time constraints. However, I reviewed the available demos, README files, Jupyter notebooks, and Python code, and they appear to be well-documented and sufficiently detailed to enable reproduction.

The models and experimental datasets are included in the Zenodo repositories, which ensures long-term accessibility and

reproducibility. Overall, the authors have made a solid effort to ensure reproducibility, and based on this assessment, it is reasonable to conclude that the code provides a usable resource for the community.

Reviewer #2

(Remarks to the Author)
*see attached file

(Remarks on code availability)

Reviewer #3

(Remarks to the Author)
The authors have clarified their main contribution consisting in a device-agnostic training methodology (training dataset & incremental learning) implemented using different network architectures (ConvNet & ResNet), and not the DNN model itself. The authors have also extended benchmarking to both RL (and advanced variants) and Deep-STORM for the ground-truth controlled experiments, and provided a dedicated psf aberration analysis.
These modifications have improved the manuscript. The following points are not critical, but I believe the authors should address them.

In the reported results, the authors do not seem to specify which architecture was used for the DAMN model explicitly for the different experiments. The manuscript would benefit from an analysis of the model's performance with the different architectures, including computational cost. Also, can the author comment on whether combining their training methodology with more advanced architectures such as transformers would further boost its generalisability.

In the astronomical validation, the authors showcase DAMN on an image of a binary star system with Pan-STARRS1, well-supported with the location of reconstructed point sources using Gaia data. It would be interesting to benchmark their results against other approaches for blind super-resolution for astronomy (e.g. Koopmans et al 2025 https://arxiv.org/abs/2512.13353). On a further note, can the authors clarify whether they performed the pairing of Pan-STARRS1 and Gaia data themselves, or were these data obtained from existing repository?

Finally, on the choice of astronomy as a validation domain, the authors have not addressed my concern convincingly. Modern astronomy has moved far beyond point-like objects, exhibiting more and more extended, complex, and intricate structures, as well as more and more crowded fields. The authors should identity more clearly applicable cases in astronomy in the manuscript.

(Remarks on code availability)
The code provides readme files for the different directories of the repository. I have experienced some minor issues when installing the code with the python version (specifically issues with python version 3.14). It would helpful if the authors check the dependencies for more recent python version and/or comment on non-supported versions.

I have only installed and tested the astronomical validation experiment (binary star system). A minor mismatch between the relative position of the red markers with respect to the point sources (see attached file). Can the authors clarify this?

Version 2:

Reviewer comments:

Reviewer #2

(Remarks to the Author)
I am very content with the responses and changes by the authors, and thank them for the manuscript improvements. I recommend publication of the manuscript in the current state.

(Remarks on code availability)
-

Reviewer #3

(Remarks to the Author)
The authors addressed most of my comments convincingly, in particular by further clarifying specific astronomical applications relevant to their approach.

In response to the authors' comments regarding DL for large astronomical surveys, we agree that this is an important emerging direction. However, it is important to note that so far many promising DL approaches are formulated as inverse-problems, and are often physics-informed rather than purely data-driven feed-forward DNNs such as the approach proposed in this work. In particular, several such methods explicitly incorporate some knowledge of the measurement process through iterative reconstruction approaches, including plug-and-play algorithms (e.g. Sureau et al. 2020; Dabbech et al. 2022;

Akhaury et al. 2024, 2025, cited by the authors). In this context, training over simulated PSF and noise variations does not guarantee robustness to the huge diversity of the applications in the sought signal and measurement process.

Regarding benchmarking against other blind deconvolution approaches, I appreciate the authors' response and suggest that a brief summary of this discussion be incorporated into the manuscript, specifically, the approaches considered and the fundamental differences in assumptions and problem formulation that make direct comparisons difficult.

(Remarks on code availability)

Open Access This Peer Review File is licensed under a Creative Commons Attribution 4.0 International License, which permits use, sharing, adaptation, distribution and reproduction in any medium or format, as long as you give appropriate credit to the original author(s) and the source, provide a link to the Creative Commons license, and indicate if changes were made.
In cases where reviewers are anonymous, credit should be given to 'Anonymous Referee' and the source.
The images or other third party material in this Peer Review File are included in the article's Creative Commons license, unless indicated otherwise in a credit line to the material. If material is not included in the article's Creative Commons license and your intended use is not permitted by statutory regulation or exceeds the permitted use, you will need to obtain permission directly from the copyright holder.
To view a copy of this license, visit https://creativecommons.org/licenses/by/4.0/

Reply to the Reviewers' comments on manuscript titled "From Stars to Molecules: AI Guided Device-Agnostic Super-Resolution Imaging"
(manuscript no. NCOMMS-25-29809-T)

Reply to Reviewer #1

In the manuscript, the authors introduce a deep-learning calibration-free method for super-resolution imaging of point-like emitters from single diffraction-limited frames called Device-Agnostic Modeling Network (DAMN). The model is trained on synthetic data covering a range of Gaussian PSFs, noise levels, and emitter densities, and is validated on both microscopy and astronomy images. Although the study is well conducted and the results are promising, several important points require clarification before I can recommend the manuscript for publication.

We thank Reviewer #1 for the positive evaluation and for recognizing the potential of our device-agnostic approach for the community. With this approach, practitioners are not required to perform demanding calibration procedures or train specific networks. Instead, they are provided with a single trained network that works out of the box for a wide variety of samples and microscope systems. We also thank the Reviewer for many helpful comments, which are addressed in detail below and have helped improve our work.

Major Concerns
1. Extensive overlap with a concurrent preprint. There are serious concerns regarding the substantial overlap between the DAMN manuscript (From Stars to Molecules: AI Guided Device-Agnostic Super-Resolution Imaging) and the recent preprint Calibration-free single-frame super-resolution fluorescence microscopy (CFCNN) by Dostálová et al., which shares two authors and much of the core content. Both works propose a calibration-free, deep-learning approach trained on synthetic data to reconstruct super-resolved images from single diffraction-limited frames. Key elements are nearly identical:
- Synthetic data generation with Gaussian PSF shapes, emitter densities, and noise levels.
- Convolutional network architectures with upsampling, LeakyReLU, and dropout.
- Benchmarking against Richardson–Lucy deconvolution.
Aside from minor architectural changes and the addition of astronomy data, there appear to be no significant technical distinctions. The DAMN manuscript must therefore: cite and discuss the concurrent preprint explicitly. State precisely what new capability, performance gain, or application scope distinguishes DAMN from the CFCNN approach reported in the preprint.

First, let us comment on this concern at a general level. While it is true that a preprint of our follow-up paper is now available on arXiv (Dostálová et al., 19 May 2025), it did not exist at the time of our initial submission (transferred to Nat. Commun. on 19 April 2025, originally submitted to Nat. Photonics on 30 March 2026). Our manuscript under consideration at Nature Communications is a unique piece of work. Specifically, it presents (i) a first device-agnostic deep-learning concept applicable to an arbitrary optical device and sample, (ii) the first optical test of super-resolution imaging with image-to-image full control of the ground truth, (iii) a comparison with classical and state-of-the-art methods (Richardson-Lucy statistical deconvolution and Deep-STORM deep-learning reconstruction), and (iv) applications of a single model to astronomy and biomedical imaging (using well-known and well-documented experimental public datasets).

After our submission, we posted the Dostálová et al. preprint, which applied the developed approach to fluorescence-molecule microscopy. There, we extended our model and elaborated the analyses for the specific case of single fluorescent molecules, using our own molecular data. We also mentioned Richardson-Lucy, but in that work, we compared primarily with localization frameworks, such as state-of-the-art multi-emitter fitting (ThunderSTORM) and a deep-learning molecule localization method (DECODE). We further thoroughly analyzed the resolution of the method (and compared it with localization limits based on the Cramér-Rao lower bound in the new version of the

work). All these technical extensions of the original, generic method are specific to fluorescence microscopy and single-molecule localization microscopy.

We must stress that this follow-up work does not contain any data from our Nature Communications submission. In other words, the Dostálová et al. preprint is a continuation of our submitted work without any overlap in data or analyses, and it will refer to and cite our manuscript once it is published. This is also supported by the fact that we submitted our first work to a general (multidisciplinary) journal, while the follow-up will be submitted to a specialized optics venue. We have also placed both works on preprint servers (arXiv and bioRxiv) because we have no need to hide our work; we are confident that future, specific applications of our device-agnostic deep-learning super-resolution can only strengthen the original work (and the impact of the potential publication in Nature Communications), rather than detract from it. Finally, we trust that the Reviewer (and the Editor) will view these two works as a sequence: a high-impact general result followed by a study targeting a specific application domain, which is common practice in science.

Having stated the previous, let us now focus on our changes to the reviewed work.

(i) We have cited and briefly discussed the follow-up work (Dostálová et al.) in the revised manuscript (note the last paragraph in the Discussion section).

(ii) We have improved both the deep-learning model and the data domain significantly. Specifically, we replaced the fully convolutional upsampling network with a ResNet architecture, which speeds up training and further slightly improves the performance. Also, we improved the training pipeline to better use a limited memory of consumer-grade GPUs, and, on a technical note, we used Nvidia RTX A5500 GPUs to train the new model. As a result, we were able to incorporate 8x upsampling and significantly enlarge the scale of the synthetic data. Namely, we expand the range of parameters of simulated imaging devices and samples, with notable inclusion of PSF asymmetries. Consequently, the deep-learning model presented in the new version of our manuscript is quite different from that in the follow-up work (Dostálová et al.).

We would also like to stress that the main novelty of our work is the new concept of device-agnostic super-resolution, not a particular deep-learning model. The fact that two different network architectures (ConvNet and ResNet) both provide unprecedented results across various data, as a result of device-agnostic training, can be seen as a clear demonstration of the power of device-agnostic training in single-emitter imaging.

(iii) “Aside… the addition of astronomy data” difference, as mentioned by the Reviewer, is actually a crucial part of the demonstration. First, the identical model (without retraining) is successfully applied to experimental data from different domains, biology and astronomy. Second, in contrast to multi-frame stacks used by SMLM methods, the astronomical data comes from a single-shot acquisition (single frame) of a dense emitter structure, where localization methods fail. In the new version of the manuscript, we made further steps in this direction to show unprecedented results in both fields. First, we have improved quantitative analysis of the attained resolution. Second, we replaced the original astronomy dataset (without ground truth) with new data, pairing a low-resolution ground-based telescope image of unresolved binary stars with the corresponding ground-truth localizations obtained from a space telescope. Third, we added a new biology dataset of nuclear pore complexes with partial ground-truth information. In all these applications, the single model without retraining and without any calibration or adjustment for a particular dataset was used. Altogether, these changes now strongly support our central claim on device-agnostic super-resolution.

2. Incomplete benchmarking against deep learning-based deconvolution algorithms. In figure 2, the manuscript only compares DAMN to Richardson–Lucy deconvolution, which is a classical method. It would be valuable to include comparisons with other deep-learning-based deconvolution algorithms (e.g., ESRGAN, CARE, RCAN, or SRResNet), or with more recent approaches based on

Richardson-Lucy (RLN) (Li, Y., et al. Nat Methods 19, 1427–1437 (2022)) or Wiener deconvolution, to better position the method relative to the current state of the art.

As suggested by Reviewer #1, we added the explicit comparison to more recent reconstruction methods in the revised manuscript. Specifically, we added comparisons with the total-variation Richardson-Lucy [Dey, et al. Microsc. Res. Tech. 69, 260 (2006)] and the *deep-learning method* Deep-STORM [Nehme, et al. Optica 5, 458 (2018)], e.g., in Figure 2 and Figure 4. Together with "classical" yet well-performing and widely used Richardson-Lucy (iterative deconvolution) and SOS Plugin (localization multi-emitter fitting), we cover various well-established methods. We also tested Wiener deconvolution, but it provides way too inferior results compared to Richardson-Lucy. On that note, the Richardson-Lucy algorithm can be viewed as an iterative maximum-likelihood method that, in the large-data limit, saturates the ultimate Cramér-Rao lower bound. So, at least under ideal conditions, the Richardson-Lucy method should be close to the optimum Poisson maximum-likelihood estimator, from a statistical point of view. This is the main reason we prefer to keep Richardson-Lucy as a reference method in our work. We added this information to the Richardson-Lucy Algorithms section in Methods.

Other methods mentioned by the Reviewer (ESRGAN, CARE, RCAN, SRResNet) are not developed and tested for single-emitter samples and, consequently, do not provide any reference for the scope of our work and datasets presented there.

In Richardson-Lucy Network (RLN) work [Li, et al. Nat Methods 19, 1427 (2022)], where RL iterations are represented as layers of a feedforward convolutional network, the authors report PSNR and SSIM metrics for a variety of samples. PSNR values can be directly converted to mean squared error (MSE), but not to mean absolute error (MAE), which we use in our work. However, for MSE, the following conclusion can be drawn: RLN achieves approximately 1.15× to 4.90× lower MSE than standard Richardson-Lucy, while in many of the main biological examples, the improvement is typically in the range of about 1.2× to 2.4× lower MSE. The expected MAE improvement is even lower, depending on the statistical distribution of errors. In contrast, DAMN outperforms Richardson-Lucy (in MAE metric) by two orders of magnitude. As a result, RLN cannot match the performance of DAMN. The reference to Li, et al. is added to the revised manuscript.

Most importantly, even if some method is on par with DAMN for a particular sample, it does not change anything on the main message of our work, i.e., all methods out there require prior knowledge on the imaging system and sample parameters (precise calibration) and, in the case of deep-learning methods, retraining for a specific setup/data. In contrast, our approach is the first truly device-independent super-resolution.

It might seem, at first glance, that we chose to sacrifice the accuracy of system-specific methods in order to achieve a device-agnostic methodology. One can expect that a super-resolution approach calibrated and trained for a specific sample and optical device would easily outperform our calibration-free approach. Surprisingly, this is not the case. The main reasons DAMN performs so well are the strong generalization ability of the model (a direct result of device-agnostic training) and the fact that the sample and imaging parameters vary across the field of view (FOV) and may also change over the course of a data acquisition. Specifically: (1) the emitter concentration is not constant across the FOV; (2) the point spread function varies due to inherent optical-system properties, typically resulting in more aberrations and broader point spread functions in the outer regions of the FOV; (3) the background is not homogeneous due to setup performance or sample properties (e.g., nonuniform background fluorescence); and (4) the signal-to-noise ratio varies for similar reasons. State-of-the-art methods, particularly deep-learning approaches trained on specific parameter values (or calibration data), cannot generalize sufficiently to this variability. In contrast, our device-agnostic model is trained across an extremely large parameter range and can generalize even beyond the training data. Consequently, our method handles these inhomogeneities effectively and produces superior results.

3. Missing quantitative metrics vs Deep-STORM. Only qualitative side-by-side images are shown. It would strengthen the analysis to include quantitative resolution metrics, such as the Fourier Ring Correlation (FRC), or similar measures, preferably mapped across the field of view (Culley, S. et al., Nat Methods 15, 263–266 (2018)), to validate the claimed performance advantage.

We agree with the Reviewer that the comparison of DAMN and Deep-STORM (Figure 6) lacks a quantitative assessment. However, the meaningful evaluation of the image resolution is extremely tricky. Two basic strategies exist: either the spatial frequency content is evaluated to assess how "sharp" features are present (without discussing if these frequencies are also present in the ground truth image), or the direct comparison with the ground truth is made (when ground truth is available).

The Fourier Ring Correlation (FRC) suggested by the Reviewer belongs to the resolution metrics computed using the spectrum of spatial frequencies. We have calculated FRC based on the two articles [Nieuwenhuizen, et al. Nature methods 10, 557 (2013); Banterle, et al. Journal of Structural Biology 183, 363 (2013)] that have independently established FRC as resolution criterion in the SMLM. However, they disagreed on which threshold for the FRC curve should be used. Upon further inspection into the choice of thresholding [van Heel & Schatz, Journal of Structural Biology 151, 250 (2005)], we have sided with [Banterle, et al.] and used the 2σ threshold. The resulting FRC curve of the Deep-STORM reconstruction does not cross this threshold (Fig. R1), rendering the resolution inconclusive even for the well-established method.

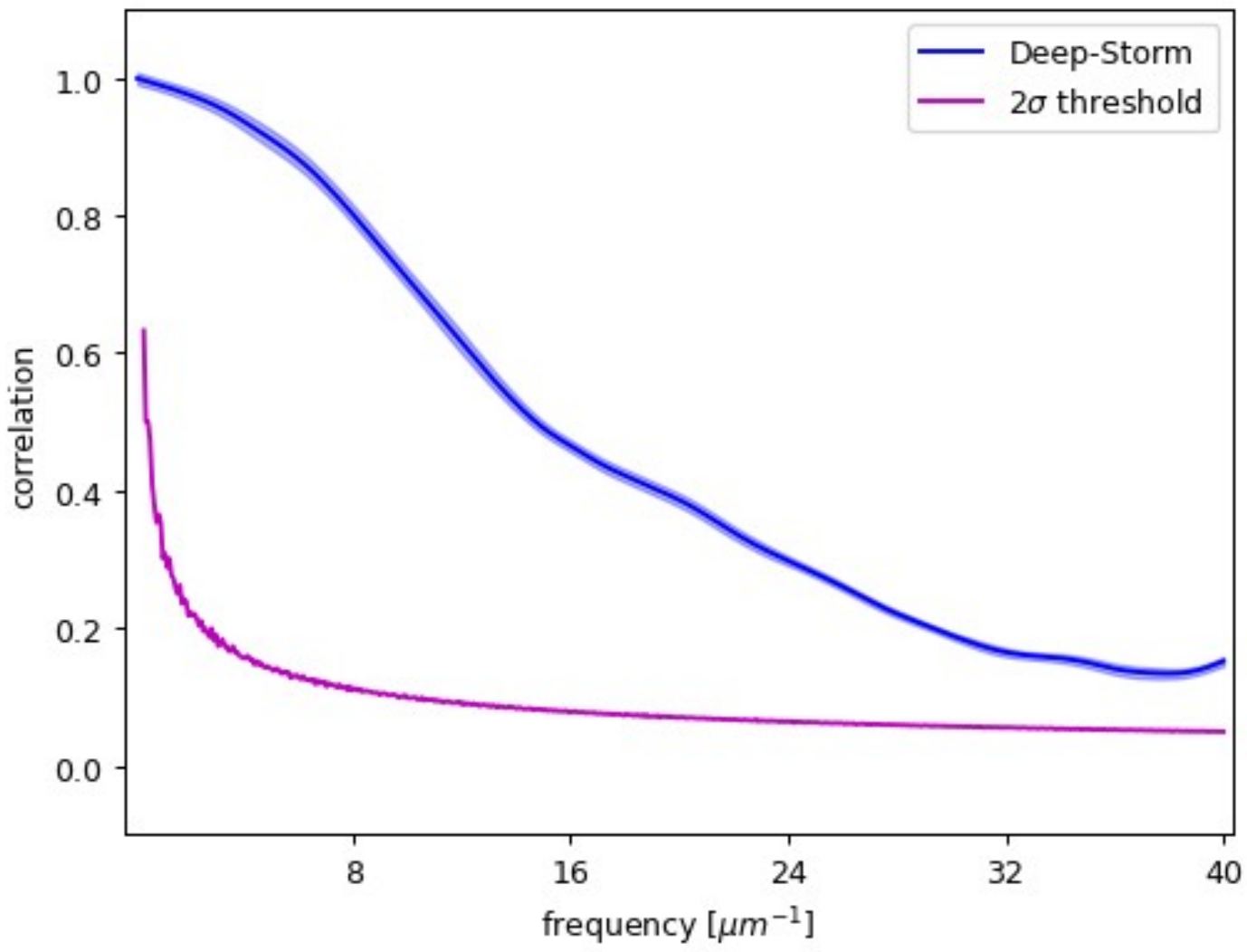

Figure R1: FRC curve of Deep-STORM and the 2σ threshold. The curves do not cross and the analysis is inconclusive, i.e., the resolution cannot be determined.

The reference suggested by the Reviewer [Li et al, Nature Methods 19, 1427–1437 (2022)] is another resolution metric combining FRC mapped across the field of view and error metric that compares super-resolved images with a widefield microscope image of the same sample. The latter has proved to be the problem as the SMLM challenge tubulin dataset was acquired only in the SMLM regime lacking the widefield image. Finally, we tried the parameter-free decorrelation analysis [Descloux, et al. Nature Methods 16, 918 (2019)], which circumvents the ambiguity of choosing a threshold in FRC, but it has inconclusive results as well. The main problem is that the first unfiltered curve should have a local maximum as in Fig. R2 **a** (adapted from the original paper), which is not the case for the Deep-STORM reconstruction, see Fig. R2 **b**.

[editorial note: third party material]

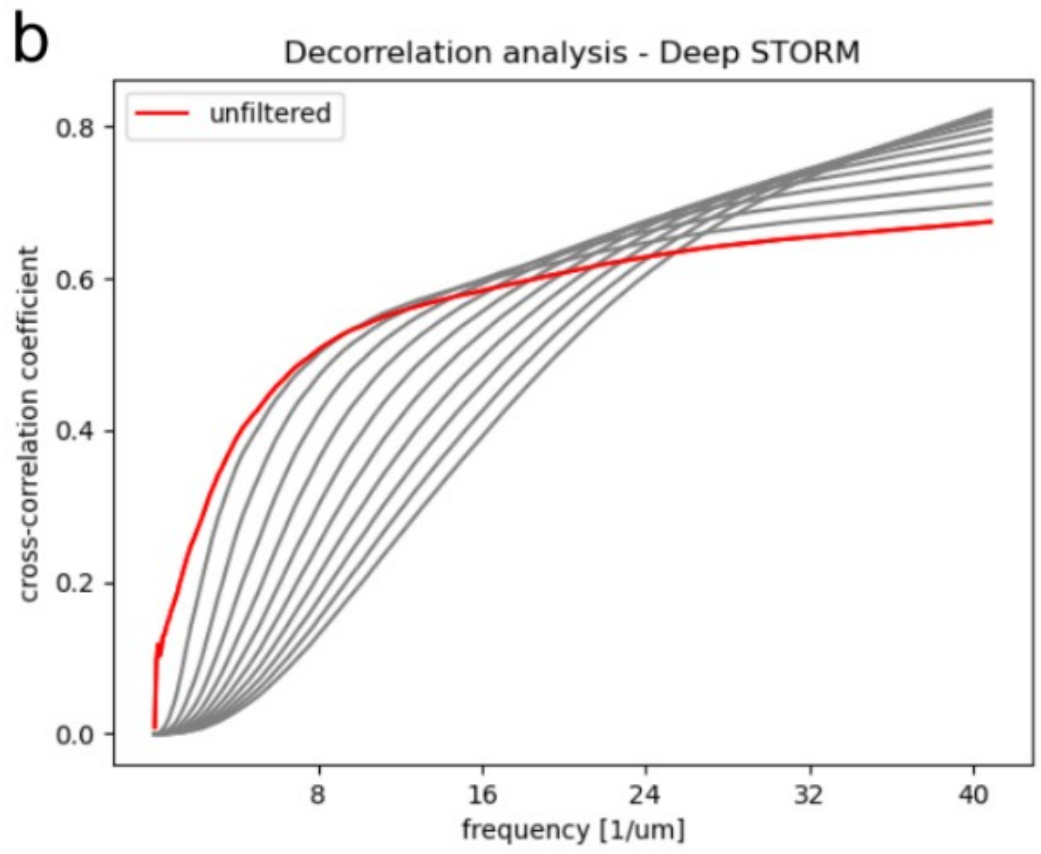

Figure R2: Parameter-free decorrelation analysis. **a**, The unfiltered curve (green) that has a local maximum. **b**, The unfiltered curve of Deep-STORM reconstruction (red) which does not have a local maximum, i.e., the analysis is inconclusive and cannot provide the resolution.

We also want to raise a concern about FRC as a resolution metric in general. The FRC is calculated from two independent image (in SMLM, obtained by integrating half of the frame-stack each). If a positive rotationally symmetric filter is applied in the Fourier domain, to either or both images, the FRC does not change, as can be readily checked and was pointed out by [van Heel, Ultramicroscopy 21, 95 (1987)]. We have applied such filter, specifically a Gaussian filter, in the Fourier domain to both image (frame-stack halves) of super-resolved reconstruction of tubulin obtained by Deep-STORM and gradually decreased the filter width (increasing the image blur). The visual representation of the filtering is presented in Fig. R3 after summing both filtered images. All these representations have in theory the same FRC, yet the actual perceived resolution is vastly different. When the FRC is calculated numerically, certain artifacts arise (presented in Fig. R4), specifically, the FRC curve suddenly rises and plateaus near unity, which is a well-documented phenomenon discussed in [van Heel & Schatz, Journal of Structural Biology 151, 250 (2005)]. To conclude, based on all the presented analyses, we do not think that FRC is a reliable measure of resolution.

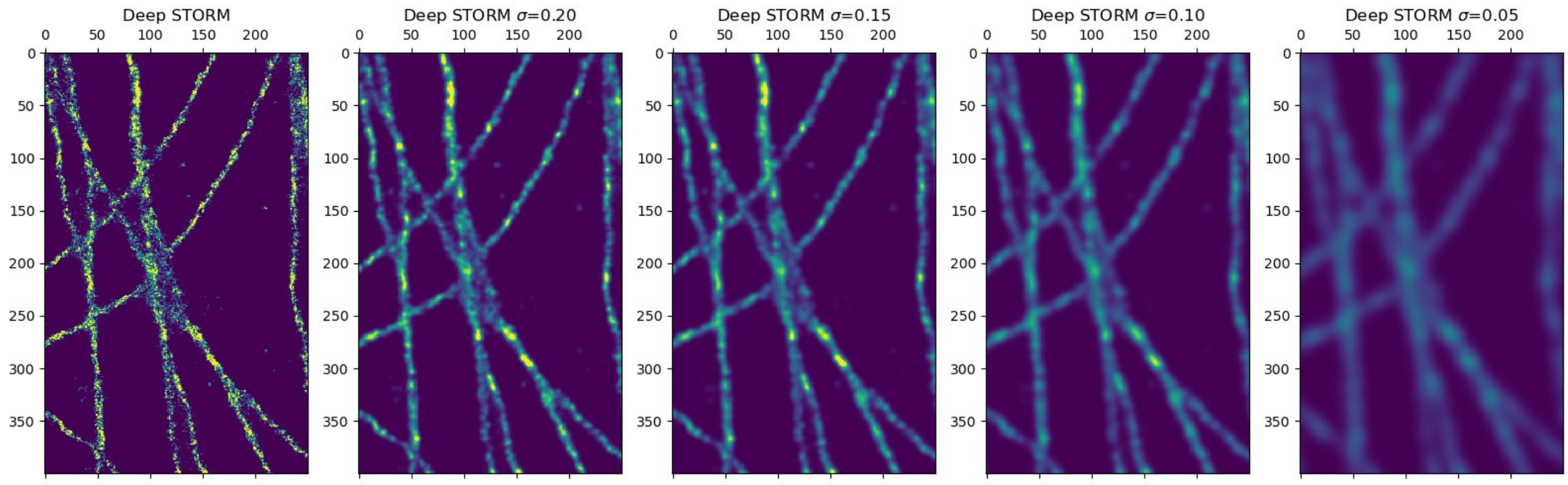

Figure R3: Gradually blurred Deep-STORM reconstruction of tubulin sample.

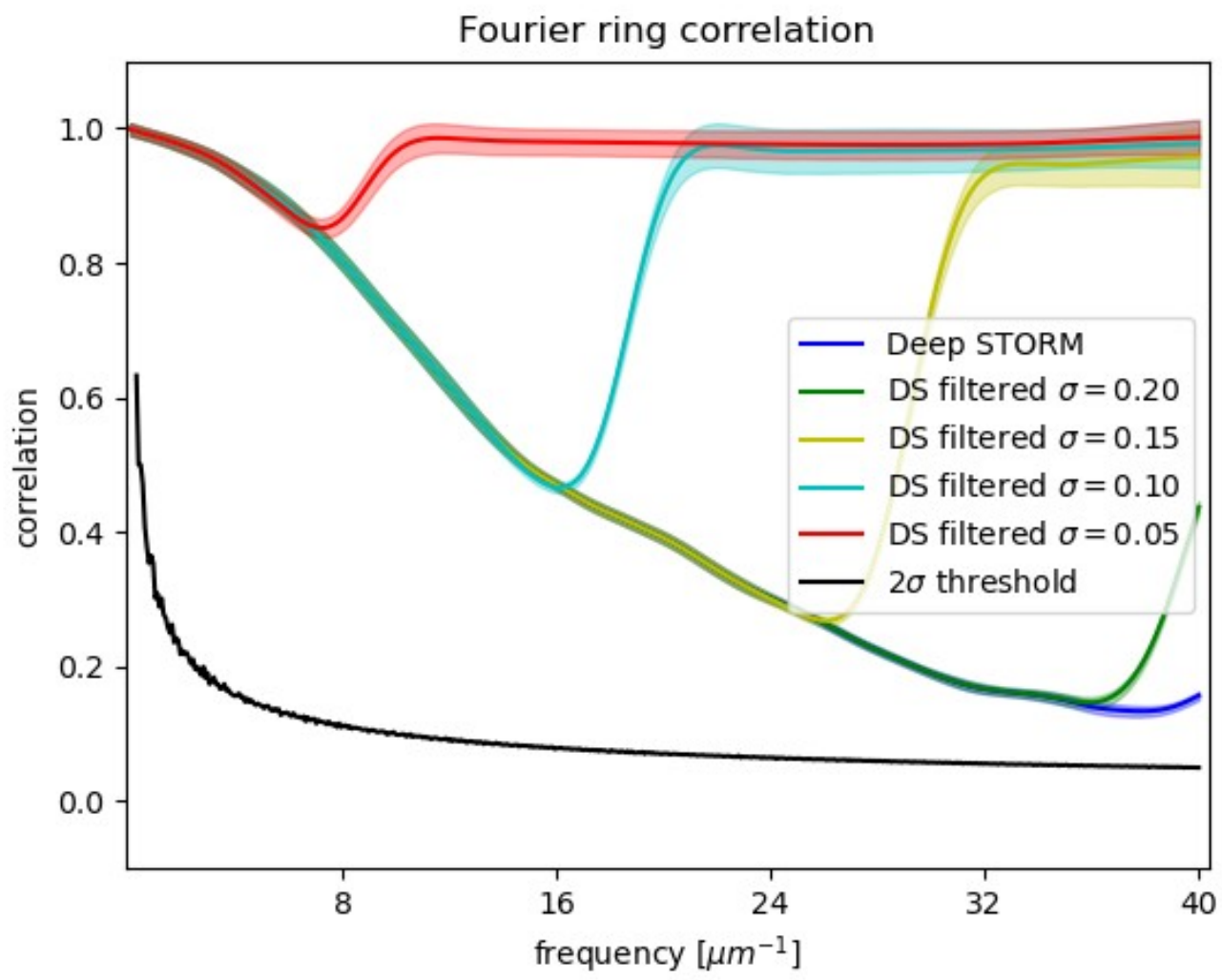

Figure R4: FRC of the original Deep-STORM tubulin reconstruction (blue) and of the same image blurred by Gaussian filters (green, yellow, cyan, red) compared to the standard 2σ resolution threshold (black).

We address the issue of quantitative resolution metrics in the revised manuscript as following.

For the optical experiment (with full ground-truth control) and the astronomy data (partial ground truth for binary stars), the super-resolution results are analyzed with respect to the Rayleigh resolution limit of the corresponding optical device used. In both cases, the reconstructed super-resolved images show the resolution well beyond the Rayleigh limit. Specifically, as explicitly stated in the revised manuscript, the Rayleigh limit is surpassed by at least a factor of 3.9 and 2.5 (17.5 for the reconstruction error) for the optical experiment and astronomy dataset, respectively.

For the tubulin dataset, we resort to comparison of the width of a microtubule in the reconstructed image. Namely, the DAMN model reaches the reconstructed width of 55 nm, as explicitly discussed in the revised manuscript, see Figure 6 (I) and the corresponding text. For comparison, the reconstruction-based Deep-STORM provides a width of 73 nm, and the localization-based SOS Plugin provides a width of 56 nm. Considering that the fluorescently stained microtubule is at least 50 nm thick [Chowdhury et al. Communications Biology 8, 499 (2025)], the actual resolution reached is probably a few nanometers (otherwise the reconstructed width would be larger). But again, no ground truth is available here, so we can hardly claim anything quantitatively. For this reason, we added another sample in the new version of the manuscript, specifically nuclear pore complexes (NPC), where the partial ground truth is available in the form of relative structural distances. Here again, DAMN performed very well, super-resolving the pores with excellent structural similarity. We evaluated reconstruction errors of pore structures to be 7 nm and 4 nm for the NPC ring diameter and the inter-emitter spacing, respectively. We hope that these results sufficiently demonstrate the excellent resolution and, mainly, high fidelity of the DAMN reconstruction.

4. Reproducibility: training code and data. The public GitHub repository currently contains only figure-generation scripts. For reproducibility, the community needs access to the training code (including network definition, loss functions, and hyperparameters); either the full or a representative portion of the synthetic training and validation sets, or the scripts and parameters required to regenerate them.

As we have already stated above, the main novelty lies in device-agnostic training. To this end, the GitHub repository already contains scripts for such training data generation. The data itself are not shared as they are excessively large for a public repository. Also, incremental training is adopted and the data are not saved during the training.

Within the current revisions, we added the full training code and other materials on the GitHub repository.

Minor Concerns

1. Clarify whether the astronomical demonstration uses the same trained network as in the microscopy experiments, or whether a fine-tuned variant was applied.

The information that the same network is used (without changes, without retraining) for both microscopy and astronomy applications was already provided in the original version of the manuscript (lines 328-329). Specifically, we wrote: “Additionally, we applied the same DAMN model, without retraining, to a publicly available dataset [37, 38] acquired for the single-molecule localization microscopy challenge.”

In the revised manuscript, we expanded and stressed this key information. For all real-world applications of DAMN demonstrated in our work, i.e., binary stars, tubulin, and nuclear pore complexes, the same (identical) model is used without any fine-tuning or retraining.

2. Considering that the training data were generated using Gaussian-modeled PSFs, it would be helpful to provide examples of typical microscopy conditions, such as recommended minimum pixel size (to satisfy the Nyquist criterion) and/or objective magnifications that correspond to the synthetic training data. This would help users avoid oversampling when applying the model.

The model delivers excellent performance when applied to images within or not too far beyond the synthetic training data domain, which is carefully specified in the Method section. Specifically, the model (upsampling) was trained on the PSF width within the range of app. 1 – 10 pixels (in combination with other parameters varied). The hardware settings of the imaging device (objective NA, magnification, camera/pixel size) are not directly relevant, as the model works in pixel space. So, any combination of objective magnification, numerical aperture, and raw camera pixel size that produces the input image with the PSF width within or close to the specified pixel range will work. Providing a particular example of typical microscopy conditions might be misleading, as DAMN can work with images acquired with a vast range of optical instruments of different designs, from microscopes to telescopes.

However, to address the Reviewer’s comment, we included an analysis of the model performance as a function of numerical aperture (for particular parameters: 532 nm, SNR = 10^3), see section Aberration analysis in Methods of the revised manuscript. It shows two important things. First, the model performance slightly decreases (a few percent error increase) for NA ≤ 0.3 simply because the PSF width exceeds the training range. Second, the analysis shows excellent generalization ability, as the NA analysis is based on full vectorial PSF calculations while the model is trained only on Gaussian and Airy-shaped PSFs of various widths and asymmetries. The real PSFs (from the full vectorial calculation) were not used in the training.

3. The authors mention that the simulated data represent emitted photons as "emitter power." It would be useful to show, perhaps in the supplementary materials, how this value and the average noise level were selected. Currently, the criteria for choosing the range of optical parameters used in the data simulation are unclear. The authors could support their choices by citing relevant references or showing examples of real data that match those conditions.

To comply with the proposed idea of device-agnostic super-resolution, the model should be trained on simulated data with the widest possible range of parameters. Simply put, the wider the range, the better the generalization ability. Obviously, the selection of parameters and their ranges is limited by the available computational resources. In our case, this corresponds to the following parameters for the non-upsampling model:

emitter power $\in [1, 10^5]$ detections per emitter,

average noise $\in$ [1, 100] detections per pixel,
emitter concentration $\in$ [5, 500],
point spread function width $\in [10^{-0.25}, 10^{1.25}]$ px ≈ [0.5, 17.75] px
point spread shape: Gaussian or Airy shaped.

Training of the upsampling model is significantly more computationally expensive. To alleviate the training process on our limited resources, we chose to slightly narrow the ranges:

emitter power $\in [1, 10^4]$ detections per emitter,
average noise $\in$ [1, 100] detections per pixel,
emitter concentration $\in$ [1, 100],
point spread function width $\in [10^{0.1}, 10^1]$ px ≈ [1.25, 10.0] px,
point spread shape: Gaussian or Airy shaped, random asymmetries.

The data simulation is described in detail in the Method section.

These ranges definitely cover and exceed values in the majority of experiments in fluorescence microscopy, astronomy, materials science, etc. For example, the typical SNR values for both fluorescence microscopy and astronomy range from 10 to 100, which lies well within our training range of $[10^{-2}, 10^4]$. Similarly, emitter power (detection counts per acquisition time) typically ranges from a few hundred to several thousand [Lelek et al., Nat. Rev. Methods Primers 1, 39 (2021), cited as Ref. 9], which lies within our training range of $[1, 10^4]$. The same holds for astronomy applications.

We demonstrate the performance of the resulting model for an astronomy dataset with binary stars and two biology datasets (tubulin and nuclear pore complexes); these three *real-world* datasets are acquired with different imaging devices and under different experimental parameters, such as emitter, intensity, SNR, emitter density, PSF width and shape, etc. Despite this, the DAMN model works nicely for all of them without any recalibration or retraining.

4. It would strengthen the manuscript to include results on biological samples beyond microtubules, such as nuclear pore complexes, whose size and spacing are well characterized. This would better demonstrate the performance of DAMN in practical scenarios and enable comparison with other super-resolution methods.

We thank the Reviewer for this comment and suggestion. In the revised manuscript, we added the application of DAMN to nuclear pore complexes and recovered the excellent structural similarity, including quantitative values, with the sample properties. We also compare the DAMN performance with Deep-STORM reconstruction.

5. It would also be helpful to test the model using a mixture of fluorescent polystyrene beads of different known sizes. This could help assess the accuracy of the model's estimations and its ability to resolve heterogeneous structures.

The whole scope of our work is within samples containing single emitters, such as stars or molecules, which appear as point-like sources, i.e., sources with angular/spatial sizes *significantly* below the resolution of an imaging device. Polystyrene beads, however, do not fall into this category.

The accuracy of the model's estimations is evaluated in the revised manuscript for an astronomy dataset of a binary star and two biology datasets (tubulin and nuclear pore complexes). Specifically, for the astronomy dataset and the nuclear nuclear pore complexes, we provided a quantitative assessment of the model's predictions with the (partial) ground truth available for these datasets. Furthermore, all three datasets are quite heterogeneous in density of emitters, their brightness, and the noise background over the field of view.

6. The manuscript contains several general claims about resolution improvement, such as in line 303: "leading to an even more prominent improvement in the resolution," and in line 385: "The analysis results undeniably show the superior performance of our approach, outperforming the Richardson-Lucy algorithm by orders of magnitude in terms of both reconstruction accuracy and

speed.” It is important to provide quantitative values to support these claims, along with the parameters used to define or compute resolution.

In the new version of the manuscript, we added the information (quantitatively) on how much we surpassed the Rayleigh resolution limit in the numerical simulations, optical experiment (with full ground truth control), astronomy data (with partial ground truth of binary star positions), and nuclear pore complexes (with partial ground truth information). For the tubulin sample (where the ground truth is not available) we added the width of the super-resolved microtubule fiber. Namely, DAMN reached the reconstructed width of 55 nm. For comparison, the reconstruction-based Deep-STORM provides a width of 73 nm, and the localization-based SOS Plugin provides a width of 56 nm. For denser parts of the field-of-view, DAMN and Deep-STORM outperform the SOS Plugin. DAMN does not require any calibration, retraining, or fine-tuning for this task, while the other two methods do.

7. The limitations of the model should be discussed, for instance, in cases where emitters are closely spaced or overlapping. This is an important consideration in super-resolution imaging of dense samples.

The limitations of the model are, in our opinion, already thoroughly analyzed and discussed in the manuscript. Figure 2 and the corresponding text show the performance of DAMN under variations in the experimental parameters and the sample. Specifically, panel **c** characterizes the dependence on the emitter concentration, referring to the collective number of emitters in a single image, ranging from 50 to approximately 700 emitters in the 50x50 pixel image. As the emitters are placed randomly, these numbers directly give an idea of their density. One can see that for more than 700 emitters randomly distributed in a 50x50 image patch, the mean absolute error of all the tested methods starts to converge to ≈ 1 (DAMN consistently providing the lowest error); this represents the limit of DAMN applicability with the current extent of training.

Furthermore, Figure 4 shows the experimental results (with full control over the ground truth) for a sample with 200 emitters in a 50x50 pixel image. Insets focus on a dense area with emitters lying in the nearby pixels (distance 1 px), well beyond the Rayleigh resolution limit of approximately 3.9 px. Experimentally, the emitters lie in the centers of the pixels with a distance of 340 nm and are imaged by a low-resolution microscope (NA = 0.25, lambda = 532 nm). The 340 nm emitter distance is 3.82× below the resolution of the microscope. All these details are included in the manuscript (see the Experiment section of Methods).

Here again, we would like to stress that the main novelty of our work is the device agnosticity and not a particular model, whose performance, already very good, is given and limited by the extent of the training. With more computational resources and, consequently, a wider range of training data variability, we expect even better performance of device-agnostic models.

8. In line 584, the authors compare the computational performance of Richardson-Lucy with DAMN, but it is not clear whether the Richardson-Lucy implementation used a GPU. Including results from a GPU-based Richardson-Lucy implementation would offer a fairer comparison with the DAMN model running on a GPU.

We do not agree with the Reviewer that we omitted the Richardson-Lucy implementation in this comparison. In the original version of the manuscript, we stated: “Using an AMD EPYC 7443P 24-core processor, the Richardson-Lucy needs almost five days to reconstruct this dataset. In comparison, the DAMN model, *utilizing the same CPU resources,* reconstructs the data in a little over two hours.”

However, we agree that the speed comparison might be tricky due to different operational principles and implementations of the compared algorithms. DAMN is deep-learning-based while the Richardson-Lucy algorithm is statistical iteration-based. Moreover, in the revised manuscript, we added the comparison also to the more recent total-variation regularized Richardson-Lucy algorithm

and Deep-STORM, which is deep-learning-based but with a different implementation. Consequently, we decided to omit speed comparison in the revised manuscript to be completely fair. Instead, we would like to primarily focus on quality performance and comparison between the methods.

Reply to Reviewer #2

The manuscript bij Vašinka et al describes a new deep-learning methods for generalized single-emitter imaging. The method is described to be capable of resolving a wide variety of conditions (e.g. scales from microscope to astronomy) with better-than-state-of-the-art accuracy. The method is well introduced, researched, described, and training data and computational methods are well described and probably reproducible. I'd like to note that I have a background in single-molecule microscopy and thus focused my review on this field.

However, I unfortunately do not, as of yet, agree with the researchers claims they made – i.e. they are either overpromising or under-delivering.

We thank Reviewer #2 for their evaluation and for many helpful comments, which are addressed in detail below and have helped improve our work.

These are my key notes:
- The authors claim to have a method that works 'without the need for calibration measurements' (e.g. lines 144-146). However, the only training data they provide is in the form of PSFs that are shaped like 2d, symmetrical Gaussians, or 2d, symmetrical Airy discs, with varying widths and intensities. This leaves out the majority of the work performed in single-molecule microscopy reconstruction over the past decade, which has been on non-ideal PSFs. There is no consideration of non-ideal PSFs, i.e. optical aberrations (Zernike coefficients), defocus, blurred emitters, refractive index mismatches, etc etc. Without these considerations, I do not believe that this method can be stated to be 'universally applicable' – it can only be applied in very specific, (near-)ideal conditions that are almost never realized in single-molecule microscopy.

Indeed, the "calibration-free" property of our method is fundamentally linked to the device-agnostic approach to the model training, and we emphasize that this is the key novelty of our methodology. The DAMN model is trained on vast synthetic data covering a wide range of sample parameters (emitter concentration, brightness, etc.) and imaging parameters (point-spread-function shapes, widths, and also asymmetries in the revised manuscript, signal-to-noise ratios, etc.). This new, unique strategy yields unprecedented generalization ability and direct applicability to diverse samples and imaging devices. In contrast, the existing methods, both classical and deep-learning based, require recalibration or retraining not only when switching between experimental conditions, but often even for different samples within the same imaging session. Moreover, existing methods generally cannot sufficiently account for parameter variations across the field of view, which are common in real systems, and their accuracy is therefore limited even though they were carefully fine-tuned for individual situations.

Our results, shown in the original submission, demonstrate that explicitly including parametrization by Zernike polynomials in the training data is not necessary for the model to perform robustly on experimental datasets. As shown in the original manuscript, our model performs well on real-world astronomy and microscopy data, where common optical aberrations are inevitably present. This provides direct evidence that the model, in its current form, can handle aberrations that were not explicitly used in the training.

However, we agree that explicit demonstration of the robustness of our model to optical aberrations is missing. So, in the revised version of the manuscript, we further substantiate the generalization ability of the model by tests of its performance for various optical aberrations (Subsection Aberration analysis and Extended Data Fig. 2 in the new version) such as astigmatism, spherical aberration,

defocus, or coma, described by the Zernike polynomials, demonstrating that the model maintains its high reconstruction quality even under conditions it was never explicitly trained for. Furthermore, we included the analysis of the model performance as a function of numerical aperture. It shows an excellent generalization ability, as the NA analysis is based on full vectorial PSF calculation, while the model is trained only on Gaussian and Airy-shaped PSFs of various widths and asymmetries. The real PSFs (from the full vectorial calculation) were not used in the training.

Moreover, in the revised manuscript, we tested our method on another biological dataset, specifically nuclear pore complexes. These results are presented in Figure 6-(II) in the revised version of the manuscript. Together with the tubulin sample (Figure 6-(I)), we validated that DAMN exhibits high reconstruction quality on real complex biological samples with a high density of emitters and a challenging background, even though it did not have access to any kind of information about the data origin. The same model was used for both the biological samples and for the astronomy dataset (binary stars) without any alterations or retraining, despite the fact that all three datasets were acquired by different imaging devices with vastly different aberrations and other parameters. This is a clear proof of our method being device-agnostic and widely applicable as a ready-to-use tool, providing rapid, high-quality, and faithful reconstructions of challenging samples.

Finally, we would like to stress that the main novelty of our work is the new concept of device-agnostic super-resolution, not a particular deep-learning model whose performance, already very good, is given and limited by the extent of the training. With more computational resources and, consequently, a wider range of training data variability (possibly including aberrated PSFs), we expect even better performance of device-agnostic models. Interestingly enough, the current model (not trained on aberrated PSFs) can cope extremely well with aberrations and real-world data, as explicitly demonstrated in the revised manuscript.

- The focus on difference between Gaussian and Airy PSFs is redundant in my opinion, since they are nearly identical, especially given that the outer rings are lost in noise.

We understand the Reviewer's point. Nevertheless based on our experience we must kindly disagree with the Reviewer's opinion on the importance of including two extreme cases of PSF shape (low-NA Airy and high-NA Gauss). When we originally trained only with one of the shapes, the generalization of the resulting model was inferior compared to the model trained on both shapes. The latter shows a strong generalization ability to various PSF shapes "in-between" for various NA values. Furthermore, the "outer rings" are not "lost in noise". Depending on the noise level, they may be masked by the noise during visual inspection, but still play a role in the performance of super-resolution methods.

In the revised manuscript, we further improved our model and its training pipeline (ResNet instead of ConvNet, training on minibatches, better parallelization, and more powerful hardware), which allowed us to expand the range of parameters of training data. Relevant to the Reviewer's comment, we included PSF asymmetries and further slightly improved the model performance.

- I strongly disagree with their statement that image reconstruction is 'the more universal approach' (line 86). In SMLM, mostly images are indeed reconstructed, but if one obtains a list of underlying emitter positions, creating an image is trivial. I.e. there is no information lost by obtaining emitter positions from a model, but there is information lost by only looking at image reconstruction. Emitter position (authors call this parameter estimation) is also the harder problem to solve.

We fully acknowledge the power of localization methods for achieving super-resolution, specifically in SMLM microscopy. However, the localization methods require the active emitters to be sparsely placed. Sparsity is achieved through temporal separation of emitter activity (blinking or photo-activated emitters). Furthermore, improving localization precision is typically done at the expense of increasing the probability of missing many emitters in a frame, which is not an issue for SMLM working with thousands of frames in a single stack. The sparsity requirement might be difficult to meet in dense samples, and missing localizations represent a significant obstacle for observing non-

blinking emitters in single-frame scenarios (i.e., basically in any other application, such as standard fluorescence microscopy, semiconductor quantum dot characterization, astronomy, etc).

By opting for image reconstruction, we aim to relax these restrictions and allow for denser labeling and even work with photostable non-blinking labels (not mentioning stars and other "stable" point-like sources) and fast single-shot acquisitions, expanding the application to a significantly wider range of imaging techniques – this is the meaning of the universality statement. Moreover, the localization workflow very often ends with a visualization step, which ultimately turns localization into reconstruction as well.

Although a localization image can, in principle, be rendered at an arbitrary resolution, the achievable accuracy of emitter localization, and consequently the true optical resolution, is fundamentally limited by the signal-to-noise ratio of the acquired signal by the Cramér-Rao lower bound (CRLB) [Thompson et al, Biophysical Journal 82(5), 2775 (2002), Mortensen et al., Nature Methods 7, 377 (2010)]. For emitters that emit simultaneously, the CRLB derived for the localization of temporally separated emitters cannot be directly applied. It is known that the Fisher information for emitter separation vanishes as the emitters approach each other, further limiting the achievable resolution [Tsang et al., Phys. Rev. X 6, 031033 (2016)]. From the point of view of these limitations, it is quite tricky to claim that "there is no information lost by obtaining emitter positions from a model."

Moreover, to reach the demonstrated resolution (see the reply to the next comment), the DAMN model contains upsampling layers (overall 8x upsampling), which makes it powerful enough to compete and potentially outperform localization techniques, as we demonstrated in our work.

Finally, we should stress again that the main novelty of our work is the new concept of device-agnostic super-resolution, not a particular deep-learning model. The same agnosticity concept (and corresponding way to training a deep learning model) can be applied to localization models similarly as to reconstruction models. But this is completely out of the scope of our current work.

- Along these same lines, the focus on image reconstruction limits their method to a certain pixel size – interpreting figure b, it's around 60-70 nm accuracy in SMLM. This is an order of magnitude worse than state-of-the-art localization methods.

This must be a misunderstanding of some sort; we have not specified 60-70 nm resolution anywhere in our manuscript. In the original manuscript, we show two deep-learning models: one without upsampling, used for thorough numerical analysis and for optical experiment with full control of ground truth, and the second, with 8x upsampling for all real-world applications. Specifically, the upsampled model is applied to an astronomy dataset and to tubulin data, where it is compared to the reference SMLM method Deep-STORM.

For the optical experiment (with ground truth) and the astronomy dataset (binary stars with ground truth from a space telescope in the revised manuscript), the super-resolution results are analyzed with respect to the Rayleigh resolution limit of the corresponding optical device used. In both cases, the reconstructed super-resolved images show the resolution well beyond the Rayleigh limit. As explicitly stated in the revised manuscript, the Rayleigh limit is surpassed *at least* by a factor of 3.9 for the optical experiment (with full control over ground truth) and 2.5 for the astronomy dataset of binary stars (with the ground truth from a space telescope), respectively. Chiefly, the error of the binary star distance reconstruction is approximately 0.08 arcsec, *which is 17.5 times below the Rayleigh resolution limit.* In both cases, there is no point in discussing nanometers as a relevant metric.

It is also important to stress that both the optical experiment and astronomy dataset represent single-shot acquisition data of stable emitters, i.e., the reconstruction is made from a single camera frame. This is in stark contrast with SMLM techniques, where thousands or tens of thousands of camera frames of blinking or photo-activated molecules are used. The achievable resolution cannot be compared between such different hardware platforms.

For the tubulin SMLM dataset, the corresponding ground truth is not available. Here, we resort to a comparison of the width of a microtubule in the reconstructed image. Namely, the DAMN upsampling model reaches the reconstructed width of 55 nm, as explicitly discussed in the revised manuscript. For comparison, the reconstruction-based Deep-STORM provides a width of 73 nm, and the localization-based SOS Plugin provides a width of 56 nm. Considering that the fluorescently stained microtubule is at least 50 nm thick [Chowdhury et al. Communications Biology 8, 499 (2025)], the actual resolution reached is probably a few nanometers. But again, no ground truth is available here, so we can hardly claim anything quantitatively. For this reason, we added another sample in the new version of the manuscript, specifically nuclear pore complexes (NPC), where the partial ground truth is available in the form of relative structural distances. Here again, DAMN performed very well, super-resolving the pores with excellent structural similarity. We evaluated a reconstruction error of pore structures to be 7 nm and 4 nm for the NPC ring diameter and the inter-emitter spacing, respectively, which is similar or smaller than what is achieved using Deep-STORM trained specifically to this dataset. We hope that these results sufficiently demonstrate the applicability of DAMN to SMLM datasets and its excellent performance.

Finally, let us emphasize that *the same model* (with upsampling) was used for *all* real-world applications presented in our work (binary stars, tubulin, nuclear pore complexes) without any alterations, fine-tuning, or retraining. This is a clear proof of our method being device-agnostic and widely applicable as a ready-to-use tool, providing high-quality and faithful reconstructions of challenging samples.

- Figure 4 shows the key SMLM showcase of accuracy, but this is a very simplified method, with uniformily identical PSFs, uniformily identical intensities, and (in my opinion most importantly), structured information about the emitter position – since the ‘emitter position’ is determined by the DMD pixel, there can never be 2 emitters closer spaced than ~65 nm, which strongly hinders all interpretation of the method.

We strongly disagree with the Reviewer’s claim that Figure 4 shows an SMLM showcase. The data depicted in Figure 4, as it was thoroughly discussed already in the original manuscript, comes from a single-frame (single-shot) optical experiment with the full control of ground truth using DMD.
Figure 4b shows a sample with 200 emitters randomly placed in a 50x50 pixel image. Insets focus on a dense area with emitters lying in the nearby pixels (distance 1 px) well beyond the Rayleigh resolution limit of approximately 3.9 px. Experimentally, the emitters lie in the centers of the pixels with a distance of 340 nm and are imaged by a low-resolution microscope (NA = 0.25, lambda = 532 nm). The 340 nm emitter distance is 3.82× below the resolution of the used microscope. The key message here is that DAMN can reconstruct a super-resolved image for a single camera frame with an unprecedented structural fidelity, and beat the Rayleigh resolution limit at least by a factor of 3.9.

Furthermore, in this and previous comments, Reviewer #2 completely ignores the second half of our manuscript and the fact that we presented two models in our original work. The non-upsampling model is used for numerical analyses and experimental data from the optical experiment with full control of ground truth (which is quite unique in the field of super-resolution methods). Then the second, more computationally demanding model with upsampling is introduced (using the same concept of device-agnostic training) and tested on real-world applications without ground truth or with partial ground truth available.

In the revised manuscript, we further expanded the scope of real-world applications and explicitly showed the excellent structural reconstruction fidelity and resolution of the upsampling model. Specifically, we reached the structure reconstruction error (compared to the available ground truth) by more than 17 times below the Rayleigh resolution limit for binary star dataset (see Lines 391 - 396). We also improved the upsampling model with the ResNet architecture.

Finally, demonstrating two device-agnostic models (ConvNet and ResNet) in our revised manuscript, we further strengthened the applicability and power of device-agnostic training. It can be applied not only to different application domains but also to different deep learning architectures and different upsampling factors.

- In the methods somewhere (I believe), the authors discuss upsampling to resolve this issues to a certain extent, but do not show this at all.

Already in the original submission, we stated explicitly that "We have also explored the benefits of incorporating upsampling layers into the DAMN convolutional architecture, further extending its capabilities....", lines 209 – 301, in the main text of the original manuscript. The whole section "Astronomical and localization microscopy" then shows and discusses in detail the application of this upsampling model to astronomy and biology datasets. So, we have to disagree with the Reviewer's comment.

In the revised manuscript, we improved the DAMN (with upsampling) demonstration on the astronomy dataset and included a new biology dataset of nuclear pore complexes. We have also improved the quantitative comparison of the DAMN results with respect to (a partial) ground truth available in these datasets and with respect to the other well-established methods.

- The SOSplugin reconstruction of the figure 6 dataset (b) is flawed in multiple ways. First of all, the SMLM challenge provides GT localizations, which can be used as reference; secondly, visualization of single molecules as scatter plots is a very flawed method – should be considering the loc. Uncertainty and visualization via upsampled gaussians, etc.

We do not mean to be blunt, but the SMLM challenge certainly does not (and cannot) provide ground truth localization for *experimental* data. It might provide ground truths for simulated data; however, the dataset used in our work is experimentally acquired, i.e., without ground truths.

Furthermore, although the visualization of the SOS Plugin result presented in our original submission might be flawed, it was presented in the exact same way in the SMLM challenge repository. However, to accommodate Reviewer #2, we redone the visualization of the SOS Plugin results based on their recommendation in the revised manuscript. Nevertheless, this changes virtually nothing in comparison of the methods.

- Finally, combining methods from astronomy and SMLM is not novel – in fact, one of the first SMLM reconstruction methods has made this connection ~15 years ago – and this paper is not mentioned here (Holden, Uphoff, Kapanidis, Nat Meth, 2011).

We agree with the Reviewer that our work is not the first super-resolution analysis using datasets from both astronomy and biology (and SMLM particularly). However, we do not claim this in our manuscript. The novel core idea of our work is that we can approach super-resolution in these (and even more) fields with a single model without (re)calibration, fine-tuning, or retraining.

Specifically, the work of Holden et al. Nature Methods 8, 279 (2011) presents the observation that an astronomy software package (DAOPHOT II) for model-based PSF fitting and star localization can be used for STORM data, i.e., sparse blinking molecule datasets, after specific PSF fitting and careful parameter tuning. However, they do not show any application of the software to both astronomy and biology data with the same setting (same calibration, same set of tuning parameters). Also, they do not demonstrate and compare the performance of the software for single-frame imaging methods. Because of these reasons, the work by Holden et al. does not differ at all from other multi-emitter fitting approaches, such as ThunderSTORM [Ovesny et al. Bioinformatics, 30, 2389 (2014)] or WTM [Takeshima, et al. Journal of Microscopy 271, 266 (2018)]. The latter actually shows that the performance of DAOSTORM (based on DAOPHOT II) is not optimum for higher density samples. Altogether, multi-emitter fitting approaches are not device-agnostic in any way, regardless of whether

they are based on astronomy or biology software packages. We included the relevant references in the revised manuscript.

Other, more minor points:
- I feel the authors overstate the problems which have already been addressed in earlier work, especially given the issue of obtaining in situ PSFs from your set-up. Many methods exist to directly extract this from a single in situ dataset.

We must kindly disagree with the Reviewer. The vast literature on PSF characterization and simulation in super-resolution imaging states the opposite. It is true that for sufficiently sparse samples, the PSF can be obtained directly from a single in situ dataset. However, it does not mean that this approach provides accurate results and is applicable in general. The main problems are:
- dense samples where emitters are overlapping (basically anything other than SMLM),
- typical low SNR impacting the PSF reconstruction accuracy (everywhere),
- aberrations making the PSF shape complex and beyond exact modeling/fitting (everywhere),
- PSF variations over the field-of-view (everywhere) and in time, i.e., within a temporal stack (in SMLM).

There are hundreds of high-impact and highly-cited works on these issues, such as [Deschout, et al. Nature Methods 11, 253 (2014), Booth, et al. Microscopy 64, 251 (2015), von Diezmannet al. Chemical Reviews 117, 7244 (2017), Xu, et al. Nature Methods 17, 531 (2020), Fu, et al., Nature Methods 20, 459-568 (2023), Liu, et al. Nature Methods 21, 1082-1093 (2024), etc.].

Besides the mentioned fundamental issues, the PSF calibration of super-resolution methods means that the reconstruction or localization procedure has to be fine-tuned or, in the case of prevailing deep learning methods, retrained for each sample or part of the sample or stack. Also, any change or (mis)alignment of an imaging setup calls for a new PSF calibration and training to keep the optimum performance.

And this is exactly the benefit of our novel device-agnostic approach. With this approach, practitioners in many fields of optical imaging (astronomy, microscopy, etc.) are not required to perform calibration procedures and train specific models. Instead, they are provided with a single trained model that works out of the box for a wide variety of samples and imaging systems.

One can naively expect that a super-resolution approach calibrated and trained for a specific sample and optical device would easily outperform our device-agnostic approach. Surprisingly, this is not the case. The main reasons DAMN performs so well are the strong generalization ability of the model (a direct result of device-agnostic training) and the fact that the sample and imaging parameters vary across the field of view (FOV) and may also change over the course of data acquisition. Specifically: (1) the emitter concentration is not constant across the FOV; (2) the point spread function varies due to inherent optical-system properties, typically resulting in more aberrations and broader point spread functions in the outer regions of the FOV; (3) the background is not homogeneous due to setup performance or sample properties (e.g., nonuniform background fluorescence); and (4) the signal-to-noise ratio varies for similar reasons. State-of-the-art methods, particularly deep-learning approaches trained on specific parameter values (or calibration data), cannot generalize sufficiently to this variability. In contrast, our device-agnostic model is trained across an extremely large parameter range and can generalize even beyond the training data. Consequently, our method handles these inhomogeneities effectively and produces superior results.

- Figure 2b and 2c, I believe, look at the same core problem. With increased PSF width, there is a problem of overlapping emitters if the emitter density is kept stable, which results in the sharp increase in error of the DAMN model.

Yes, indeed, Figures 2b and 2c show a similar scaling in the performance of super-resolution reconstruction methods with increasing overlap of emitters. In Figure 2b, the number of emitters is constant in the image (N = 50). and the PSF width is increasing. In Figure 2c, the PSF width is fixed

(sigma = 2 px) while the number of emitters in the image is increasing. These two scalings are complementary but not identical. Some methods can cope better with a high number of “sharp” emitters than a few very “blurry” emitters, and vice versa. This is the reason why we keep both panels in Figure 2.

In the new version of the manuscript, we added two new reference methods in Figure 2, namely TV Richardson-Lucy and Deep-STORM. Together with the notorious Richardson-Lucy, these three methods demonstrate various sensitivity to the number of emitters, PSF width and shape, and SNR. For all combinations of parameters, DAMN outperforms these three methods. For extreme cases, such as excessively large PSF or number of emitters larger than 700 in the 50x50 image, all methods saturate at mean absolute error ≈ 1. We deliberately chose to plot these graphs up to those extreme cases to show the ultimate limits of our particular model. To get some perspective on what the case with N = 200 emitters looks like, see Figure 4b.

- I’m unsure why the Richardson-Lucy deconvolution isn’t applied on the fig6 data as well, seeing as it’s the author’s go-to comparison method up to that point.

First, let us note that in the revised manuscript, the iterative total-variation regularized Richardson-Lucy and the deep learning Deep-STORM are also used as reference methods in Figure 2 and Figure 4. It means that Deep-STORM is now also a go-to comparison method.

The Richardson-Lucy does not inherently incorporate upsampling, so the output reconstructed image would be of the same size as the input image. In contrast, Deep-STORM as well as DAMN both contain upsampling, so their comparison is fair.

Reply to Reviewer #3

The authors introduce a deep learning (DL) model for super-resolution imaging and demonstrate its applicability on both microscopy and astronomical data. They also describe an optical setup designed to acquire pairs of low and high resolution images for validation. The authors claim a device-agnostic DL model. However, there are serious concerns regarding methodological novelty, benchmarking and validation, and the choice of the application domain.

We thank Reviewer #3 for their evaluation and for many helpful comments, which are addressed in detail below and have helped to improve our work.

Firstly, the proposed model is essentially a straightforward, fully data-driven architecture with incremental learning, presenting limited methodological novelty for computational imaging. The literature review omits state-of-the-art DL approaches, including plug-and-play, deep unrolling (see Kamilov et al., 2022, arXiv:2203.17061), and diffusion-based reconstruction approaches. These approaches typically generalise more effectively than purely data-driven DL models and should be discussed.

First, we would like to stress that the main novelty of our work is the new concept of device-agnostic training, not a particular deep-learning model. In fact, in our original submission, two models were trained using the device-agnostic approach, both being fully convolutional, one without upsampling and the other with 8x upsampling. In the revised manuscript, we changed the architecture of the upsampling model from ConvNet to ResNet to speed up the training and slightly improve the performance. The fact that two different network architectures (ConvNet and ResNet) both provide unprecedented results across various data, as a result of device-agnostic training, can be seen as a clear demonstration of the power of device-agnostic training in single-emitter imaging. Most importantly, if “simple” network architecture outperforms all the tested, well-established methods (classical and deep learning) over various real-world datasets, what is the point of using more cumbersome or computationally demanding architectures? The “simplicity” of the used architecture

should not be a reason to discard unprecedented results caused by the proposed, completely novel device-agnostic methodology.

The scare quotes in the previous paragraph are used intentionally. The apparent simplicity of our models might be misleading. Both DAMN models contain more than 7 million trainable parameters and were trained on approximately 3 million simulated image data pairs generated numerically for a vast range of sample and imaging device parameters (emitter concentration, emitter brightness, noise level, and PSF width, shape, and asymmetry). To our best knowledge, DAMN is the largest model trained on the most extensive dataset in the field of single-emitter super-resolution imaging.

It is true that several approaches have moved beyond purely data-driven training by incorporating physical models into the deep learning framework. Examples include plug-and-play methods, which combine learned denoisers with classical iterative reconstruction schemes, and deep unrolling, which embeds the imaging algorithm into a trainable, iteration-inspired architecture [Li, et al. Nature Methods 19, 1427 (2022); Kamilov, et al. IEEE Signal Processing Magazine 40, 85 (2023)]; these references were added in the revised manuscript (see lines 146-157 and references 37 and 38). However, none of these methods possesses device-independence or calibration-free property as our DAMN. Incorporating physical priors might even worsen the specificity of such methods. To conclude, all methods out there require prior knowledge on imaging systems and sample parameters (in form of precise calibration or priors) and, in the case of deep-learning methods, retraining for specific setup/data. In contrast, our approach is the first truly device-independent super-resolution.

Secondly, benchmarking solely against the classical Richardson–Lucy (RL) algorithm (Richardson 1972; Lucy 1973) is not sufficiently motivated. The authors should clarify why RL is considered "state-of-the-art" in this context rather than comparing against established and recent approaches used in the relevant imaging domains.

Richardson-Lucy algorithm can be viewed as an iterative maximum-likelihood method that, in an ideal large-data limit, saturates the ultimate Cramér-Rao lower bound. So, at least under ideal conditions, the Richardson-Lucy method should be close to the optimum Poisson maximum-likelihood estimator, from a statistical point of view. This is the main reason we chose Richardson-Lucy as a reference method in our work. We have added this explanation in the revised manuscript (see the Richardson-Lucy Algorithms section of Methods).

To address the Reviewer's suggestion, we toned down the "state-of-the-art" to "well-established". Furthermore, we added two other reference methods, namely, more recent iterative total-variation (TV) Richardson-Lucy [Dey, et al. Microsc. Res. Tech. 69, 260 (2006)] and deep-learning method Deep-STORM [Nehme, et al. Optica 5, 458 (2018)], throughout the manuscript. TV RL is designed to suppress unstable oscillations and mitigate noise-amplification artifacts inherent to the classical RL formulation; it represents a modern, improved variant of RL applicable as a universal deconvolution algorithm in many fields, for example, astronomy. Deep-STORM, on the other hand, is a well-established, deep-learning method for image reconstruction in the SMLM field.

Moreover, in the comparison with RL, strict stopping criteria were adopted, including extremely small tolerance (10^-10) and a very large iteration number (10^6), which raises concerns about the fairness of the comparison and undermines claims of computational efficiency. On a further note, given the simple nature of RL iteration structure, it could have been easily deployed on GPU similarly to the proposed DL model.

To attain the maximum-likelihood estimator and, potentially, saturate the Cramér-Rao lower bound (as discussed in the reply to the previous comment), Richardson-Lucy has not been early stopped. This is the reason we adopted the main stopping criterion to be a small difference between two successive iterations (10^{-10}, close to the machine precision). The upper limit to the number of iterations (10^{6}) is rarely reached. Typical number of iterations is 10^{4} – 10^{5}.

Furthermore, in the original version of the manuscript, we stated: “Using an AMD EPYC 7443P 24-core processor, the Richardson-Lucy needs almost five days to reconstruct this dataset.
In comparison, the DAMN model, utilizing *the same CPU resources,* reconstructs the data in a little over two hours.” So we compared Richardson-Lucy and DAMN using the same hardware (CPU).

However, we agree that the speed comparison might be tricky due to different operational principles and implementations of the compared algorithms. DAMN is deep-learning-based, while the Richardson-Lucy algorithm is statistical iteration-based. Moreover, in the revised manuscript, we added the comparison also to the more recent total-variation Richardson-Lucy algorithm and Deep-STORM, which is deep-learning-based but with a different implementation. Consequently, we decided to omit speed comparison in the revised manuscript to be completely fair. Instead, we would like to primarily focus on the quality performance of the methods.

For the real single-molecule microscopy data, although Deep-STORM is considered in the comparison, performance gains are marginal and computational cost is not discussed.

In the revised manuscript, we added the explicit quantitative comparison of DAMN, Deep-STORM, and the SOS Plugin on tubulin data. Specifically, for a single super-resolved microtubule fiber (Figure 6-(l)), DAMN reached the reconstructed width of 55 nm. For comparison, the reconstruction-based Deep-STORM provides a width of 73 nm, and the localization-based SOS Plugin provides a width of 56 nm, so DAMN wins here. For denser parts of the field-of-view, DAMN and Deep-STORM significantly outperform the SOS Plugin, while DAMN is slightly better than Deep-STORM. Again, it is important to stress that DAMN does not require any calibration, retraining, or fine-tuning for this task, while the other two methods do.

The computational cost is similar for DAMN and Deep-STORM in inference tasks. However, Deep-STORM has to be trained based on actual calibration, which takes up to a few hours based on the complexity of the problem and the hardware available. In contrast, DAMN is provided as a to-go trained model, which is applied without retraining, saving a great deal of time to practitioners in various fields.

As for the real astronomical illustration, the analysis of the results is not supported with a rigorous validation (including the accuracy of the position and flux of the resolved point sources), thus limiting the interpretability of the results.

We thank the Reviewer for this comment. We agree that the original manuscript lacks quantitative validation for the astronomy dataset due to the missing ground truth. For this reason, we replaced the astronomy dataset with a new one. In the revised manuscript, we used a segment of the Andromeda galaxy (M31) survey containing a completely unresolved (2.5 times below the Rayleigh limit) binary star imaged by a ground-based telescope (Pan-STARRS1). As a ground truth, we utilize known catalogized coordinates, based on the Gaia space-based observatory. The DAMN model reconstructs two stars nicely with almost the exact distance between them. Specifically, the error of the binary star distance reconstruction is approximately 0.08 arcsec, which is 17.5 times below the Rayleigh resolution limit of the ground-based telescope. See lines 391-396 and Figure 5.

Finally, the choice of astronomy as a validation domain is not adequate. Given the current landscape of modern telescopes, astronomical imaging often involves extended sources, and compact sources with complex morphology. It also requires algorithms that scale efficiently to large surveys, making the current approach difficult to generalise, thus severally limiting its impact in this domain.

Generally speaking, deep-learning methods are more frequently used also because of their nice scaling, at least in the inference tasks, where the trained model is applied on data in a feed-forward way. Extensive surveys can be processed per segment or preprocessed as a whole by underperforming, but fast methods, and then the selected parts can be refined by highly-performing but computationally more demanding methods. The true bottleneck of deep-learning methods is their

training stage, where the model is (re)trained based on specific calibration data (PSF, SNR, density, etc.). And this is exactly the point, where our DAMN excels – it is trained once on a vast but synthetic (numerically generated) data. Once trained, the model is ready to be applied to a wide range of single-emitter datasets without any alterations, fine-tuning, or retraining. This is why the proposed device-agnostic approach is so unique and potentially useful.

Reply to the Reviewers' comments on manuscript titled "From Stars to Molecules: AI Guided Device-Agnostic Super-Resolution Imaging"

Reply to Reviewer #1

In their revised version of the manuscript, the authors have carefully addressed my questions and concerns (both major and minor). Not only my comments, but also those of the other reviewers, have been addressed thoroughly.

The paper has improved quite a bit, and all issues have been resolved to my full satisfaction. Additionally, the GitHub repository has improved significantly, including the Zenodo additions.

I therefore recommend acceptance of this manuscript for publication.

Remarks on code availability:

Unfortunately, I could not install and run the code myself due to time constraints. However, I reviewed the available demos, README files, Jupyter notebooks, and Python code, and they appear to be well-documented and sufficiently detailed to enable reproduction.

The models and experimental datasets are included in the Zenodo repositories, which ensures long-term accessibility and reproducibility. Overall, the authors have made a solid effort to ensure reproducibility, and based on this assessment, it is reasonable to conclude that the code provides a usable resource for the community.

Since no additional concerns were raised, we want to thank the Reviewer for their time and effort in evaluating our work and for many helpful suggestions,which improved our work. We are pleased to receive the positive feedback and their recommendation to accept our manuscript for publication.

Reply to Reviewer #2

We thank the Reviewer for their evaluation and the additional comments, which helped further improve our manuscript. To improve clarity and limit the length of our response, we include only the Reviewer's new comments and our corresponding responses.

[Ad. Validation of the reconstruction quality using complex and aberrated PSFs.]
I appreciate that the authors validated that their NN works well on aberrated PSFs. Ext. data fig 2 remedies most of my issues. The only point I'd like to make is that it is important to (also) test astigmatism in out-of-focus plane rather than just in-focus. This is a common artefact in SMLM (and cannot be easily corrected for from a sample point of view), and breaks the radial symmetry assumption. As such, it's interesting to see the behavior there. I also appreciate fig 6-II.

We are glad to hear the positive feedback. As requested, we have added out-of-focus astigmatism to the analysis. All aberration types, including the currently added out-of-focus astigmatism, increased the mean absolute error of the reconstructed super-resolved images only by up to 3% for aberration strength up to 0.2 lambda, compared to the case without any aberrations (see Extended Data Fig. 2).

I am not sure about the authors claim about "main novelty of our work is the new concept of device-agnostic super-resolution". Device-agnostic SMLM has been bedrock until very recently (i.e. simple Gaussian fits are device-agnostic). The main novelty is, in my opinion, a *deep-learning

model* which is device-agnostic. As such, the particular model and performance cannot simply be excluded from consideration. Nonetheless, I don't believe the authors have phrased it as such in the manuscript itself, so I don't see a real issue.

We may have formulated the intended claim inaccurately in our previous response. However, since the manuscript phrases our intentions properly, we consider this issue resolved.

[Ad. Including Gaussian and Airy profiles in the training results in improved generalization ability.]
I'm interested to hear that the authors have tested this and indeed saw a difference. I would love to see a sentence about this in the discussion.

We have added the corresponding comment on this topic in the revised version of our manuscript, see lines 776-781: "The inclusion of both distinct shapes is important for unlocking the high generalization ability. When we originally trained only on one of the shapes, the generalization of the resulting model was inferior compared to that of the model trained on both shapes."

[Ad. Localization vs. reconstruction, which is the more universal or generalizable.]
I fully follow the author's reasoning in this reply, but I feel like they didn't answer my concern: Image reconstruction is not the more universal (nor the more generalizable, as mentioned in the new manuscript) approach for SMLM. The fact that the DAMN network only results in images, not in localization data, is irrevant to that statement. I fully agree that, when just trying to do image reconstruction, it is easier to create an algorithm/network that is capable of doing this, but this only underpins my comment that image generation for SMLM is not the more universal or generalizable approach. It is simply the approach that is easier to computationally implement. This should in my opinion still be changed in the manuscript.

We have removed the claim comparing the universality and/or generalizability of localization and reconstruction methods from the manuscript. While we might still have a different opinion on the topic, given that this opinion could raise concerns in the community, we decided to avoid such comparisons in the manuscript.

[Ad. Limitations to certain pixel sizes.]
This indeed is my bad, coming from my confusion. I was assuming that the non-upsampled model was used throughout. I apologize for the confusion, and the ~8x upsampled model indeed pretty much reaches CRLB using normal pixel sizes and normal emitter intensities. I thank you for the clarification. I also feel that the NPC pores help in this discussion.

We are glad that the misunderstanding has been resolved and thank the Reviewer for the positive feedback on NPC pores.

[Ad. The optical experiment.]
OK, sure, I guess figure 4 shows a showcase of a single SMLM frame. My point remains. This is an accuracy showcase of a set of (GT-known) emitters in a SMLM-capable microscope, where the GT can be compared to the inferred position. This is important for this manuscript, and I appreciate that the authors have included this. However, my point remains that (to add values here), the 200 emitters placed in a 50x50 px image are spaced at least 1 pixel apart on the DMD mask.

Looking at figure 4b, top-right image (i.e. the DMD groundtruth reconstructed to the camera image), assuming a Rayleigh limit of ~200 nm (visible light, high NA), which is, according to the author's scalebar of R, set to to ~4px, means that every pixel is ~50nm in size. This still means that in the DMD-imposed GT, 2 emitters are always spaced at least 50 nm apart, which is not a proper GT dataset. (in real emitters, the random position of emitters is uniform from 0 to infinity, not from 50 to infinity). In the lowNA case that the authors describe in the rebuttal, this is then making the case worse, as no 2 emitters are ever placed closer than 340 nm, which is, again, not a proper realistic dataset.

Thus, the fact that the authors claim “The non-upsampling model is used for numerical analyses and experimental data from the optical experiment with full control of ground truth” is not true. There is not full control of ground truth, there is control of the ground truth up to a difference of 340 nm (or whatever) between emitters. If (hypothetically) 2 emitters placed apart ~100 nm cannot be discriminated from 1 emitter, the network doesn’t work as it should work in theory. Figure 4, as it stands, does not show this metric. Thus, it is in my opinion not a good validation for optical microscopy. As such, this performance metric cannot be interpreted.

First, let us note that the information on the minimum distance of the emitters (as well as their FWHM width) was already explicitly stated in *”Methods: Experiment,”* see lines 996-999 in the revised version. These “point-like” emitters are formed in the emitter plane as shown in Extended Data Fig. 1. They are imaged by the low-resolution imaging microscope. The resolution limit of this microscope is 1.3 um, which is 4 times larger than the minimum emitter distance of 340 nm, see lines 1009-1012 in the revised version.

We understand the objection of Reviewer #2 to our claim in the main text, specifically, *“...with full control of ground truth.”* We agree that this claim might be perceived as incorrect. Due to the discrete nature of all spatial light modulators (including the DMD device used in our experiment), we work with emitters in a discrete rectangular grid with the mentioned minimum distance. However, we do have full control over which emitters are switched on or off, which is what we meant by *“full control.”* In the revised manuscript, we clarified and toned down the objected statement.

We would like to stress that this clarification does not change the results and impact of our work. The minimal distance between the emitters in the optical experiment is already 4 times below the resolution limit of the imaging microscope, and, most importantly, all tested methods (non-upsampling DAMN, Richardson-Lucy, TV Richardson-Lucy, and Deep-STORM) use the same low-resolution images for inputs and are compared against the same ground truth masks. I.e., all the methods are analyzed under the same conditions.

Finally, we appreciate that the Reviewer considers our optical experiment “important for the manuscript.” It is indeed important, as an experiment with full knowledge and partial control over ground truth bridges the gap between numerical simulations (with full control and full ground truth knowledge) and real-world data (with partial or no ground truth). We further emphasize this importance in the revised manuscript.

[Ad. Upsampling being already included in the manuscript.]
I thank the authors for the clarification here.

We are glad to have resolved this.

[Ad. Data from the SMLM challenge.]
Right. The authors are correct that they used an experimental dataset of the SMLM challenge, and I apologize that I missed this point.

I change my question to: why did the authors use an experimental dataset of the SMLM challenge (now properly mentioned in the text, not before), while the SMLM challenge was conceived to create “synthetic data that represent three-dimensional structures modeled after biological components, taking excitation parameters, noise sources, point-spread functions and pixelation into account” (taken literally from the cited paper). The SMLM challenge was specifically created to have realistic simulated data with GT available to test SMLM algorithms on. It is specifically created so that authors (like these) that have new algorithms (like this manuscript) have realistic data to test their algorithm on.

So, let me rephrase my point:
First of all, the SMLM challenge provides GT localizations, which can be used as reference. The authors should use GT-available data from this challenge.

While we acknowledge the advantages of the SMLM synthetic datasets, we have already conducted a comprehensive evaluation on simulated data (see sections *Evaluation using simulated datasets* and *Methods: Aberration analysis*) with known ground truth, covering the full device-agnostic regime:

- 1 – 10px widths of PSF,
- full Gauss-to-Airy PSF shape transition,
- 0.2 – 1.4 numerical apertures,
- 10^0 – 10^5 values of SNR,
- 50 – 750 emitter concentrations,
- 5 distinct types of optical aberrations with 0 – 0.2λ aberration strengths,
- etc.

Including additional synthetic data (for specific imaging conditions) in the manuscript would be redundant. Therefore, we instead included experimental SMLM data to demonstrate performance under real conditions.

Furthermore, in the previous revision, we added astronomy data and nuclear pore complexes data, with partial structural ground truth available. The results show that our DAMN accurately reconstructs structures well beyond the Rayleigh resolution limit of imaging devices. Together with the tubulin data and broad simulated datasets, the manuscript provides extensive, detailed evaluations across both simulated and experimental imaging conditions.

I appreciate that the authors have refrained from using scatter plots from the SOS plugin. I disagree that it changes nothing, as the data is much more interpretable from a human visual perspective.

We are glad to have resolved this.

[Ad. Additional reference.]
I thank that the authors included this reference.

We thank the Reviewer for suggesting it.

[Ad. In-situ PSF estimation.]
I feel the authors misinterpret me. I don’t want to state that in my opinion, by definition, specifically-trained models should outperform general ones. I stated that obtaining in situ PSFs is not specifically challenging anymore.

The problems that the authors claim here:
- dense samples where emitters are overlapping (basically anything other than SMLM),
- typical low SNR impacting the PSF reconstruction accuracy (everywhere),
- aberrations making the PSF shape complex and beyond exact modeling/fitting (everywhere),
- PSF variations over the field-of-view (everywhere) and in time, i.e., within a temporal stack (in SMLM).

Are not problems if you place fluorescent beads on your microscope (fixes point 1, 2), and fit with some proper algorithm (for instance the cited Liu, et al. Nature Methods 21, 1082-1093 (2024) , fixes point 3 and 4). PSFs do not vary over time unless optical components are moving over time.

The rest of the rebuttal text on this point is moot. I stated “I feel the authors overstate the problems which have already been addressed in earlier work, especially given the issue of obtaining in situ PSFs from your set-up.”. The response starting at “Besides the mentioned fundamental issues” is

only about why a general model is good (which I agree with, and never stated to have a problem with in my point.

We thank the Reviewer for clarifying this comment. The issue is clearly whether “obtaining in situ PSFs is specifically challenging or not.” First, this argument clearly supports the motivation for device-agnostic super-resolution. Without device agnosticity, the models must be calibrated and retrained for each specific imaging device. In contrast, our DAMN is trained once and can be readily used across a wide range of imaging devices without calibration or retraining. Even if in situ PSF calibration is possible, this does not change the fact that it represents an extra step, which has to be performed for specific super-resolution approaches and consumes personnel and computational time. We already discussed this in the previous version of the manuscript (lines 102–114):
“The reconstruction quality is inherently linked to the accuracy of the point spread function estimate; any inaccuracies will lead to poor reconstruction results [33]. However, its precise estimation entails additional calibration measurements involving an isolated emitting point source. This condition can prove demanding, especially in systems with a high concentration of emitters, low signal-to-noise ratio, or temporally developing systems. Additionally, imaging systems commonly exhibit variations in the shape of the point spread function across the image. Consequently, reconstructing larger areas may require separate calibration for each segment [34].”
Based on the Reviewer’s comment, we removed *“very”* from *“very demanding”* and added the reference to Liu et al., Nature Methods 21, 1082 (2024).

Second, we agree that in situ PSF estimation is feasible as an extra step in standard room-temperature fluorescence microscopes, using methods such as Xu et al., Nature Methods 17, 531 (2020) and Liu et al., Nature Methods 21, 1082–1093 (2024), which we have not objected to in our manuscript. However, the proposed device-agnostic super-resolution targets a much wider range of applications than fluorescence microscopy or SMLM microscopy. What is feasible in fluorescence microscopy, such as placing fluorescent beads or some kind of calibration sample on a microscope, might be quite difficult in microscopes that include a cryostat or vacuum, as in semiconductor quantum dot imaging [Vašinka et al., preprint arXiv:2510.06076 (2025)] or electron microscopy, respectively, not to mention the much more complex sample preparation. In astronomy, such calibration is obviously not feasible at all. We added the corresponding discussion to the revised manuscript, see lines 110-116.

Finally, the temporal variation of the PSF and other parameters (SNR, background, etc.) is also briefly mentioned in our manuscript. For example, maintaining the exact position between a sample and a microscope objective within a few nm over long acquisitions in SMLM is challenging, and because of this, the PSF changes slightly over the time stack. Another example is atmospheric turbulence in ground-based telescopes in astronomy, where the PSF changes on much faster timescales. We are not claiming that these issues cannot be mitigated to some extent with passive/active stabilization or adaptive optics, but again, this represents extra steps that are costly in hardware or computational time. Instead, we propose a device-agnostic super-resolution approach that can, to some extent, handle these PSF fluctuations inherently. Our arguments here are supported, for example, by the following works:
Aristov et al., ZOLA-3D allows flexible 3D localization microscopy over an adjustable axial range. Nat. Commun. 9, 2409 (2018);
Lelek et al., Single-molecule localization microscopy, Nat. Rev. Methods Primers 1, 39 (2021);
Qiu et al., Reinforced optical cage systems enable drift-free single-molecule localization microscopy, Commun. Eng. 5, 13 (2026);
Beckers, Adaptive Optics for Astronomy: Principles, Performance, and Applications, Annu. Rev. Astron. Astrophys. 31, 13–62 (1993);
Guo et al., Direct observation of atmospheric turbulence with a video-rate wide-field wavefront sensor, Nat. Photonics 18, 935 (2024).

[Ad. Inclusion of regularized Richardson-Lucy and Deep-STORM.]
I appreciate the authors effort on this, and it improves the manuscript.

We agree with the Reviewer and thank them for the positive feedback.

[Ad. Richardson-Lucy not used for comparison with the upsampling model.]
Perfect, this satisfies my comment.

We are glad to have resolved this.

Reply to Reviewer #3

The authors have clarified their main contribution consisting in a device-agnostic training methodology (training dataset & incremental learning) implemented using different network architectures (ConvNet & ResNet), and not the DNN model itself. The authors have also extended benchmarking to both RL (and advanced variants) and Deep-STORM for the ground-truth controlled experiments, and provided a dedicated psf aberration analysis.
These modifications have improved the manuscript. The following points are not critical, but I believe the authors should address them.

We thank the Reviewer for their time in evaluating the paper, and we address the individual points below.

In the reported results, the authors do not seem to specify which architecture was used for the DAMN model explicitly for the different experiments. The manuscript would benefit from an analysis of the model's performance with the different architectures, including computational cost. Also, can the author comment on whether combining their training methodology with more advanced architectures such as transformers would further boost its generalisability.

We have added explicit clarification in the revised manuscript regarding which architecture was used in each experiment. In particular, *"ConvNet (applied to simulated data and optical experiment)"* and *"ResNet-based architecture incorporating residual connections and an inherent eightfold upsampling (applied to astronomy, aberrations, and biological samples)."* This information is also documented in the GitHub repository.

Regarding the performance, we believe that both architectures are evaluated in great detail throughout the manuscript, across numerous demonstrations. Directly comparing the trained architectures is an ill-posed problem, since each provides reconstructions at different upsampling/sizes, which skews any immediate quantitative comparison. This is analogous to the reason given to Reviewer #2 for omitting Richardson-Lucy for comparison with the upsampling model on the tubulin dataset.

In the revised manuscript, we have included the computational cost of both architectures. It is important to note that, unlike most deep learning approaches, our model is trained only once and works across imaging platforms. Therefore, for its application, the relevant computational cost is fully determined by the (very fast) inference regime.

Finally, using transformers or other advanced architectures could potentially further improve the generalisability. However, since we have never used or tested transformers for any application, we cannot accurately comment on their performance in general, beyond mere assumptions.

In the astronomical validation, the authors showcase DAMN on an image of a binary star system with Pan-STARRS1, well-supported with the location of reconstructed point sources using Gaia data. It would be interesting to benchmark their results against other approaches for blind super-

resolution for astronomy (e.g. Koopmans et al 2025 https://arxiv.org/abs/2512.13353). On a further note, can the authors clarify whether they performed the pairing of Pan-STARRS1 and Gaia data themselves, or were these data obtained from existing repository?

We thank the Reviewer for acknowledging the improvements in the astronomy validation. We indeed performed the pairing between Pan-STARRS1 and Gaia, so no repository is cited in this context.

Regarding the suggested comparison with [Koopmans et al. 2025], we note that their approach is not directly comparable to ours. Specifically, their method requires multiple input images of the same region acquired at different wavelengths: *"The input consists of 24, 250, 350, and 500 μm images of the same area,"* whereas our framework operates on a single intensity input image. Moreover, the 24 μm input image already represents a high-resolution input channel, see the following Figure R1. While we understand the benefits of four images (with high-resolution information) as inputs for the intended application in the [Koopmans et al 2025] paper, their method is not comparable to our low-to-high-resolution framework using a single intensity frame in a device-agnostic regime.

[editorial note: third party material]

Figure R1. The deep-learning model of [Koopmans et al. 2025] inputs a high-resolution image (24 um) and low-resolution images (250 um, 350 um, and 500 um) and outputs a high-resolution image targeting the 500 um channel.

We also explored the possibility of benchmarking our double-star results with "other approaches for blind super-resolution for astronomy." However, existing methods do not seem directly comparable due to fundamental differences in underlying assumptions and problem formulation. In particular, we considered:

- [Hirsch et al., Astronomy & Astrophysics 531, A9 (2011)], which addresses multiple-frame inputs where each additional frame gradually improves the reconstruction quality, and only a MATLAB demo example seems to be publicly available;
- [Sukurdeep et al., Astronomy and Computing 53, 100999 (2025)] that employs multiple exposures of the same sky region, relies on calibration procedures, and is tied to a specific point spread function (although blind extension is possible). Additionally, the software is not publicly available;
- [Campagne, arXiv:2601.08666 (2026)], which focuses on enhancing the resolution of galaxy images, operating on continuous object structures rather than point-like emitters;

None of which would provide for a feasible and fair benchmark method for "blind astronomy super-resolution" on the presented single-frame data of a double-star.

It is important to note that our device-agnostic approach can also be, in principle, adapted to use multiple input astronomy frames, directly reconstruct images of objects (instead of point-like sources), or perform localization rather than image reconstruction. The device-agnostic framework would provide the same benefits independently of the underlying applications. However, such alterations are beyond the scope of our work, which focuses on single-frame image reconstruction.

Based on the situation, and to at least partially accommodate the Reviewer's request, we evaluated the blind version of the Richardson-Lucy (RL) algorithm. The results are shown in the

following Figure R2. Since the RL reconstruction does not provide upsampling, the comparison is not strictly fair, and we do not include it in the revised manuscript. However, given the low quality of the blind-RL reconstructed image, it is immediately apparent that upsampling cannot save it. Not only are the stars not resolved, but the image is also almost completely lost to amplified noise artifacts. To be thorough, we also evaluated classical and regularized RL provided with a point-spread-function estimate, yielding similar results. Given that we have already benchmarked our method against several super-resolution approaches throughout the manuscript, and the Gaia reference information fully supports the results of the astronomy application, we would like to consider this issue closed.

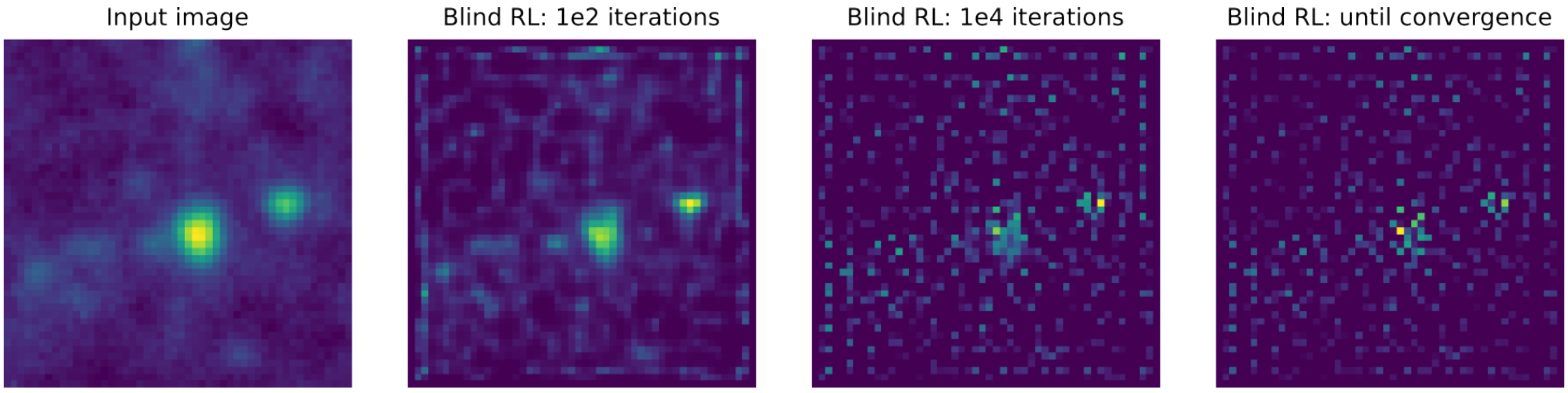

Figure R2. The gradual progression of the blind Richardson-Lucy algorithm using an initial (Gaussian) kernel estimate that strongly resembles the underlying point spread function.

Finally, on the choice of astronomy as a validation domain, the authors have not addressed my concern convincingly. Modern astronomy has moved far beyond point-like objects, exhibiting more and more extended, complex, and intricate structures, as well as more and more crowded fields. The authors should identity more clearly applicable cases in astronomy in the manuscript.

We thank for the clarification of the comment. We agree with Reviewer #3 that our method is not limitless and is not applicable to arbitrary areas of astronomy research. This is the reason we stress its applicability to images of single emitters or point-like sources, even dense ones. For example, we claim:
"Numerous applications rely specifically on imaging point-like or single-emitter sources. In biology, single-molecule localization microscopy enables nanoscale visualization of cellular structures [7–12]. Quantum physics leverages super-resolution for characterizing quantum dots [13] and precisely imaging cold atoms in optical lattices [14, 15]. Astronomy benefits through resolving individual stars and galaxies [16, 17]." at lines 27-35,
"We use it to train a device-agnostic modeling network (DAMN) designed to reconstruct intensity images of point-like emitting sources," at lines 164-166,
and in many other places in the previous version of our manuscript. In the revised manuscript, we further clarify that in astronomy, our method may be particularly beneficial for stellar astronomy and other sub-domains that deal with point-like sources. We hope this clarifies the issue of more complex and intricate structures.

Furthermore, we would like to further elaborate on the issue of extended large surveys and crowded fields of point-like sources, even though it does not appear to be the main concern in the updated comment of the Reviewer.
Recent literature does not support the claim that deep-learning-based super-resolution is inherently unsuitable for large astronomical surveys. On the contrary, several studies explicitly motivate DL methods by survey-scale demands, including millions of images or petabyte-scale data, and emphasize fast inference after training. This is shown for galaxy surveys and upcoming Rubin/Euclid data, wide-field radio super-resolution and radio-interferometric imaging designed for large next-generation surveys, and sub-mm super-resolution methods developed for wide-field Herschel data:
Schawinski et al., Mon. Not. R. Astron. Soc. Lett. 467, L110 (2017);
Sureau et al., Astron. Astrophys. 641, A67 (2020);

Lauritsen et al., Mon. Not. R. Astron. Soc. 507, 1546 (2021);
Dabbech et al., Astrophys. J. Lett. 939, L4 (2022);
Connor et al., Mon. Not. R. Astron. Soc. 514, 2614 (2022);
Akhaury et al., Astron. Astrophys. 688, A6 (2024);
Akhaury et al., Astron. Astrophys. 697, A136 (2025);
Huertas-Company and Lanusse, Publ. Astron. Soc. Aust. 40, e001 (2023).
The Reviewer's concern may apply to conventional device-specific deep-learning methods that require retraining for each instrument/configuration. However, our method is designed precisely to remove this limitation. The model is trained once on a broad synthetic ensemble covering wide ranges of PSF width and shape, signal and background levels, emitter density, and related imaging conditions. It then performs a very fast single-pass feed-forward inference without recalibration, retraining, or explicit knowledge of instrument parameters (within some limits, such as the point-like sources assumption). The manuscript demonstrates this transfer directly on Pan-STARRS1 survey data. Even if generalization is ultimately limited, such a model remains highly valuable for rapid first-stage screening and identification of further regions of interest. The selected regions can then be further examined, if needed, by a more refined device-agnostic model, by a specifically trained instrument-dependent model, or by a computationally more demanding iterative statistical deconvolution.

The code provides readme files for the different directories of the repository. I have experienced some minor issues when installing the code with the python version (specifically issues with python version 3.14). It would helpful if the authors check the dependencies for more recent python version and/or comment on non-supported versions.

We are sorry to hear that there were problems with setting up the Python environments. We have tested them across two independent Linux machines, and both the environment and the code worked as intended. Perhaps the problem lies in the Reviewer using Python 3.14 instead of the required 3.11.3, as specified in the GitHub repository? For this reason, the repository contains the requirements files in each relevant directory so users can install the tested environment (Python version, library versions, and CUDA version).

We agree that adding support for newer Python and TensorFlow versions would be beneficial. We intend to develop the repository further and add this support. Unfortunately, our main author, who developed the relevant codebase and maintains the GitHub repository, is currently fully occupied finishing their PhD and will include the support across versions in a few months. However, this does not detract from our study in any way, as in the specified and tested environment, the repository is fully functional.

I have only installed and tested the astronomical validation experiment (binary star system). A minor mismatch between the relative position of the red markers with respect to the point sources (see attached file). Can the authors clarify this?

We would like to thank the Reviewer for this great observation. Unfortunately, we made a mistake when correcting the subpixel mismatch between the extracted low-resolution Pan-STARRS1 image and the reference Gaia positional information, which are based on different catalogs. Due to the presence of such a mismatch, we did not evaluate any metric relative to the star positions in the first place and focused only on their relative distance, which is independent of an arbitrary origin offset. Therefore, no evaluated results were affected by this mistake beyond the visualization.

In the revised manuscript, we have fixed the presented image and updated the GitHub repository. We have also added localization uncertainties to the reference positions and analyzed the reconstructed star magnitudes to further strengthen the demonstration.

Reply to the Reviewers' comments on manuscript titled "From Stars to Molecules: AI Guided Device-Agnostic Super-Resolution Imaging"

Reply to Reviewer #2

I am very content with the responses and changes by the authors, and thank them for the manuscript improvements. I recommend publication of the manuscript in the current state.

We thank the Reviewer for their evaluations, which helped to improve our manuscript, and their recommendation to publish.

Reply to Reviewer #3

The authors addressed most of my comments convincingly, in particular by further clarifying specific astronomical applications relevant to their approach.

We thank the Reviewer for their time in evaluating the paper.

In response to the authors' comments regarding DL for large astronomical surveys, we agree that this is an important emerging direction. However, it is important to note that so far many promising DL approaches are formulated as inverse-problems, and are often physics-informed rather than purely data-driven feed-forward DNNs such as the approach proposed in this work. In particular, several such methods explicitly incorporate some knowledge of the measurement process through iterative reconstruction approaches, including plug-and-play algorithms (e.g. Sureau et al. 2020; Dabbech et al. 2022; Akhaury et al. 2024, 2025, cited by the authors). In this context, training over simulated PSF and noise variations does not guarantee robustness to the huge diversity of the applications in the sought signal and measurement process.

This is a good remark, and we are glad to have had this discussion, which helped to improve our manuscript and our future research.

Regarding benchmarking against other blind deconvolution approaches, I appreciate the authors' response and suggest that a brief summary of this discussion be incorporated into the manuscript, specifically, the approaches considered and the fundamental differences in assumptions and problem formulation that make direct comparisons difficult.

As requested, we have added a brief summary in the manuscript based on the previous discussions with the Reviewer.

Reply to Reviewer #2

The manuscript bij Vašinka et al describes a new deep-learning methods for generalized single-emitter imaging. The method is described to be capable of resolving a wide variety of conditions (e.g. scales from microscope to astronomy) with better-than-state-of-the-art accuracy. The method is well introduced, researched, described, and training data and computational methods are well described and probably reproducible. I'd like to note that I have a background in single-molecule microscopy and thus focused my review on this field.

However, I unfortunately do not, as of yet, agree with the researchers claims they made – i.e. they are either overpromising or under-delivering.

We thank Reviewer #2 for their evaluation and for many helpful comments, which are addressed in detail below and have helped improve our work.

These are my key notes:
- The authors claim to have a method that works 'without the need for calibration measurements' (e.g. lines 144-146). However, the only training data they provide is in the form of PSFs that are shaped like 2d, symmetrical Gaussians, or 2d, symmetrical Airy discs, with varying widths and intensities. This leaves out the majority of the work performed in single-molecule microscopy reconstruction over the past decade, which has been on non-ideal PSFs. There is no consideration of non-ideal PSFs, i.e. optical aberrations (Zernike coefficients), defocus, blurred emitters, refractive index mismatches, etc etc. Without these considerations, I do not believe that this method can be stated to be 'universally applicable' – it can only be applied in very specific, (near-)ideal conditions that are almost never realized in single-molecule microscopy.

Indeed, the "calibration-free" property of our method is fundamentally linked to the device-agnostic approach to the model training, and we emphasize that this is the key novelty of our methodology. The DAMN model is trained on vast synthetic data covering a wide range of sample parameters (emitter concentration, brightness, etc.) and imaging parameters (point-spread-function shapes, widths, and also asymmetries in the revised manuscript, signal-to-noise ratios, etc.). This new, unique strategy yields unprecedented generalization ability and direct applicability to diverse samples and imaging devices. In contrast, the existing methods, both classical and deep-learning based, require recalibration or retraining not only when switching between experimental conditions, but often even for different samples within the same imaging session. Moreover, existing methods generally cannot sufficiently account for parameter variations across the field of view, which are common in real systems, and their accuracy is therefore limited even though they were carefully fine-tuned for individual situations.

Our results, shown in the original submission, demonstrate that explicitly including parametrization by Zernike polynomials in the training data is not necessary for the model to perform robustly on experimental datasets. As shown in the original manuscript, our model performs well on real-world astronomy and microscopy data, where common optical aberrations are inevitably present. This provides direct evidence that the model, in its current form, can handle aberrations that were not explicitly used in the training.

However, we agree that explicit demonstration of the robustness of our model to optical aberrations is missing. So, in the revised version of the manuscript, we further substantiate the generalization ability of the model by tests of its performance for various optical aberrations (Subsection Aberration analysis and Extended Data Fig. 2 in the new version) such as astigmatism, spherical aberration,

defocus, or coma, described by the Zernike polynomials, demonstrating that the model maintains its high reconstruction quality even under conditions it was never explicitly trained for. Furthermore, we included the analysis of the model performance as a function of numerical aperture. It shows an excellent generalization ability, as the NA analysis is based on full vectorial PSF calculation, while the model is trained only on Gaussian and Airy-shaped PSFs of various widths and asymmetries. The real PSFs (from the full vectorial calculation) were not used in the training.

Moreover, in the revised manuscript, we tested our method on another biological dataset, specifically nuclear pore complexes. These results are presented in Figure 6-(II) in the revised version of the manuscript. Together with the tubulin sample (Figure 6-(I)), we validated that DAMN exhibits high reconstruction quality on real complex biological samples with a high density of emitters and a challenging background, even though it did not have access to any kind of information about the data origin. The same model was used for both the biological samples and for the astronomy dataset (binary stars) without any alterations or retraining, despite the fact that all three datasets were acquired by different imaging devices with vastly different aberrations and other parameters. This is a clear proof of our method being device-agnostic and widely applicable as a ready-to-use tool, providing rapid, high-quality, and faithful reconstructions of challenging samples.

Finally, we would like to stress that the main novelty of our work is the new concept of device-agnostic super-resolution, not a particular deep-learning model whose performance, already very good, is given and limited by the extent of the training. With more computational resources and, consequently, a wider range of training data variability (possibly including aberrated PSFs), we expect even better performance of device-agnostic models. Interestingly enough, the current model (not trained on aberrated PSFs) can cope extremely well with aberrations and real-world data, as explicitly demonstrated in the revised manuscript.

I appreciate that the authors validated that their NN works well on aberrated PSFs. Ext. data fig 2 remedies most of my issues. The only point I'd like to make is that it is important to (also) test astigmatism in out-of-focus plane rather than just in-focus. This is a common artefact in SMLM (and cannot be easily corrected for from a sample point of view), and breaks the radial symmetry assumption. As such, it's interesting to see the behavior there. I also appreciate fig 6-II.

I am not sure about the authors claim about "main novelty of our work is the new concept of device-agnostic super-resolution". Device-agnostic SMLM has been bedrock until very recently (i.e. simple Gaussian fits are device-agnostic). The main novelty is, in my opinion, a *deep-learning model* which is device-agnostic. As such, the particular model and performance cannot simply be excluded from consideration. Nonetheless, I don't believe the authors have phrased it as such in the manuscript itself, so I don't see a real issue.

- The focus on difference between Gaussian and Airy PSFs is redundant in my opinion, since they are nearly identical, especially given that the outer rings are lost in noise.

We understand the Reviewer's point. Nevertheless based on our experience we must kindly disagree with the Reviewer's opinion on the importance of including two extreme cases of PSF shape (low-NA Airy and high-NA Gauss). When we originally trained only with one of the shapes, the generalization of the resulting model was inferior compared to the model trained on both shapes. The latter shows a strong generalization ability to various PSF shapes "in-between" for various NA values. Furthermore, the "outer rings" are not "lost in noise". Depending on the noise level, they may be masked by the noise during visual inspection, but still play a role in the performance of super-resolution methods.

In the revised manuscript, we further improved our model and its training pipeline (ResNet instead of ConvNet, training on minibatches, better parallelization, and more powerful hardware), which allowed us to expand the range of parameters of training data. Relevant to the Reviewer's comment, we included PSF asymmetries and further slightly improved the model performance.

I'm interested to hear that the authors have tested this and indeed saw a difference. I would love to see a sentence about this in the discussion.

- I strongly disagree with their statement that image reconstruction is 'the more universal approach' (line 86). In SMLM, mostly images are indeed reconstructed, but if one obtains a list of underlying emitter positions, creating an image is trivial. I.e. there is no information lost by obtaining emitter positions from a model, but there is information lost by only looking at image reconstruction. Emitter position (authors call this parameter estimation) is also the harder problem to solve.

We fully acknowledge the power of localization methods for achieving super-resolution, specifically in SMLM microscopy. However, the localization methods require the active emitters to be sparsely placed. Sparsity is achieved through temporal separation of emitter activity (blinking or photo-activated emitters). Furthermore, improving localization precision is typically done at the expense of increasing the probability of missing many emitters in a frame, which is not an issue for SMLM working with thousands of frames in a single stack. The sparsity requirement might be difficult to meet in dense samples, and missing localizations represent a significant obstacle for observing non-blinking emitters in single-frame scenarios (i.e., basically in any other application, such as standard fluorescence microscopy, semiconductor quantum dot characterization, astronomy, etc).

By opting for image reconstruction, we aim to relax these restrictions and allow for denser labeling and even work with photostable non-blinking labels (not mentioning stars and other "stable" point-like sources) and fast single-shot acquisitions, expanding the application to a significantly wider range of imaging techniques – this is the meaning of the universality statement. Moreover, the localization workflow very often ends with a visualization step, which ultimately turns localization into reconstruction as well.

Although a localization image can, in principle, be rendered at an arbitrary resolution, the achievable accuracy of emitter localization, and consequently the true optical resolution, is fundamentally limited by the signal-to-noise ratio of the acquired signal by the Cramér-Rao lower bound (CRLB) [Thompson et al, Biophysical Journal 82(5), 2775 (2002), Mortensen et al., Nature Methods 7, 377 (2010)]. For emitters that emit simultaneously, the CRLB derived for the localization of temporally separated emitters cannot be directly applied. It is known that the Fisher information for emitter separation vanishes as the emitters approach each other, further limiting the achievable resolution [Tsang et al., Phys. Rev. X 6, 031033 (2016)]. From the point of view of these limitations, it is quite tricky to claim that "there is no information lost by obtaining emitter positions from a model."

Moreover, to reach the demonstrated resolution (see the reply to the next comment), the DAMN model contains upsampling layers (overall 8x upsampling), which makes it powerful enough to compete and potentially outperform localization techniques, as we demonstrated in our work.

Finally, we should stress again that the main novelty of our work is the new concept of device-agnostic super-resolution, not a particular deep-learning model. The same agnosticity concept (and corresponding way to training a deep learning model) can be applied to localization models similarly as to reconstruction models. But this is completely out of the scope of our current work.

I fully follow the author's reasoning in this reply, but I feel like they didn't answer my concern: Image reconstruction is not the more universal (nor the more generalizable, as mentioned in the new manuscript) approach for SMLM. The fact that the DAMN network only results in images, not in localization data, is irrevant to that statement. I fully agree that, when just trying to do image reconstruction, it is easier to create an algorithm/network that is capable of doing this, but this only underpins my comment that image generation for SMLM is not the more universal or generalizable approach. It is simply the approach that is easier to computationally implement. This should in my opinion still be changed in the manuscript.

- Along these same lines, the focus on image reconstruction limits their method to a certain pixel size – interpreting figure b, it's around 60-70 nm accuracy in SMLM. This is an order of magnitude worse than state-of-the-art localization methods.

This must be a misunderstanding of some sort; we have not specified 60-70 nm resolution anywhere in our manuscript. In the original manuscript, we show two deep-learning models: one without upsampling, used for thorough numerical analysis and for optical experiment with full control of ground truth, and the second, with 8x upsampling for all real-world applications. Specifically, the upsampled model is applied to an astronomy dataset and to tubulin data, where it is compared to the reference SMLM method Deep-STORM.

For the optical experiment (with ground truth) and the astronomy dataset (binary stars with ground truth from a space telescope in the revised manuscript), the super-resolution results are analyzed with respect to the Rayleigh resolution limit of the corresponding optical device used. In both cases, the reconstructed super-resolved images show the resolution well beyond the Rayleigh limit. As explicitly stated in the revised manuscript, the Rayleigh limit is surpassed *at least* by a factor of 3.9 for the optical experiment (with full control over ground truth) and 2.5 for the astronomy dataset of binary stars (with the ground truth from a space telescope), respectively. Chiefly, the error of the binary star distance reconstruction is approximately 0.08 arcsec, *which is 17.5 times below the Rayleigh resolution limit.* In both cases, there is no point in discussing nanometers as a relevant metric.

It is also important to stress that both the optical experiment and astronomy dataset represent single-shot acquisition data of stable emitters, i.e., the reconstruction is made from a single camera frame. This is in stark contrast with SMLM techniques, where thousands or tens of thousands of camera frames of blinking or photo-activated molecules are used. The achievable resolution cannot be compared between such different hardware platforms.

For the tubulin SMLM dataset, the corresponding ground truth is not available. Here, we resort to a comparison of the width of a microtubule in the reconstructed image. Namely, the DAMN upsampling model reaches the reconstructed width of 55 nm, as explicitly discussed in the revised manuscript. For comparison, the reconstruction-based Deep-STORM provides a width of 73 nm, and the localization-based SOS Plugin provides a width of 56 nm. Considering that the fluorescently stained microtubule is at least 50 nm thick [Chowdhury et al. Communications Biology 8, 499 (2025)], the actual resolution reached is probably a few nanometers. But again, no ground truth is available here, so we can hardly claim anything quantitatively. For this reason, we added another sample in the new version of the manuscript, specifically nuclear pore complexes (NPC), where the partial ground truth is available in the form of relative structural distances. Here again, DAMN performed very well, super-resolving the pores with excellent structural similarity. We evaluated a reconstruction error of pore structures to be 7 nm and 4 nm for the NPC ring diameter and the inter-emitter spacing, respectively, which is similar or smaller than what is achieved using Deep-STORM trained specifically to this dataset. We hope that these results sufficiently demonstrate the applicability of DAMN to SMLM datasets and its excellent performance.

Finally, let us emphasize that *the same model* (with upsampling) was used for *all* real-world applications presented in our work (binary stars, tubulin, nuclear pore complexes) without any alterations, fine-tuning, or retraining. This is a clear proof of our method being device-agnostic and widely applicable as a ready-to-use tool, providing high-quality and faithful reconstructions of challenging samples.

This indeed is my bad, coming from my confusion. I was assuming that the non-upsampled model was used throughout. I apologize for the confusion, and the ~8x upsampled model indeed pretty much reaches CRLB using normal pixel sizes and normal emitter intensities. I thank you for the clarification. I also feel that the NPC pores help in this discussion.

- Figure 4 shows the key SMLM showcase of accuracy, but this is a very simplified method, with uniformily identical PSFs, uniformily identical intensities, and (in my opinion most importantly), structured information about the emitter position – since the 'emitter position' is determined by the

DMD pixel, there can never be 2 emitters closer spaced than ~65 nm, which strongly hinders all interpretation of the method.

We strongly disagree with the Reviewer's claim that Figure 4 shows an SMLM showcase. The data depicted in Figure 4, as it was thoroughly discussed already in the original manuscript, comes from a single-frame (single-shot) optical experiment with the full control of ground truth using DMD.
Figure 4b shows a sample with 200 emitters randomly placed in a 50x50 pixel image. Insets focus on a dense area with emitters lying in the nearby pixels (distance 1 px) well beyond the Rayleigh resolution limit of approximately 3.9 px. Experimentally, the emitters lie in the centers of the pixels with a distance of 340 nm and are imaged by a low-resolution microscope (NA = 0.25, lambda = 532 nm). The 340 nm emitter distance is 3.82× below the resolution of the used microscope. The key message here is that DAMN can reconstruct a super-resolved image for a single camera frame with an unprecedented structural fidelity, and beat the Rayleigh resolution limit at least by a factor of 3.9.

Furthermore, in this and previous comments, Reviewer #2 completely ignores the second half of our manuscript and the fact that we presented two models in our original work. The non-upsampling model is used for numerical analyses and experimental data from the optical experiment with full control of ground truth (which is quite unique in the field of super-resolution methods). Then the second, more computationally demanding model with upsampling is introduced (using the same concept of device-agnostic training) and tested on real-world applications without ground truth or with partial ground truth available.

In the revised manuscript, we further expanded the scope of real-world applications and explicitly showed the excellent structural reconstruction fidelity and resolution of the upsampling model. Specifically, we reached the structure reconstruction error (compared to the available ground truth) by more than 17 times below the Rayleigh resolution limit for binary star dataset (see Lines 391 - 396). We also improved the upsampling model with the ResNet architecture.

Finally, demonstrating two device-agnostic models (ConvNet and ResNet) in our revised manuscript, we further strengthened the applicability and power of device-agnostic training. It can be applied not only to different application domains but also to different deep learning architectures and different upsampling factors.

OK, sure, I guess figure 4 shows a showcase of a single SMLM frame. My point remains. This is an accuracy showcase of a set of (GT-known) emitters in a SMLM-capable microscope, where the GT can be compared to the inferred position. This is important for this manuscript, and I appreciate that the authors have included this. However, my point remains that (to add values here), the 200 emitters placed in a 50x50 px image are spaced at least 1 pixel apart on the DMD mask.

Looking at figure 4b, top-right image (i.e. the DMD groundtruth reconstructed to the camera image), assuming a Rayleigh limit of ~200 nm (visible light, high NA), which is, according to the author's scalebar of R, set to to ~4px, means that every pixel is ~50nm in size. This still means that in the DMD-imposed GT, 2 emitters are always spaced at least 50 nm apart, which is not a proper GT dataset. (in real emitters, the random position of emitters is uniform from 0 to infinity, not from 50 to infinity). In the lowNA case that the authors describe in the rebuttal, this is then making the case worse, as no 2 emitters are ever placed closer than 340 nm, which is, again, not a proper realistic dataset.

Thus, the fact that the authors claim "The non-upsampling model is used for numerical analyses and experimental data from the optical experiment **with full control of ground truth**" is not true. There is not full control of ground truth, there is control of the ground truth up to a difference of 340 nm (or whatever) between emitters. If (hypothetically) 2 emitters placed apart ~100 nm cannot be discriminated from 1 emitter, the network doesn't work as it should work in theory. Figure 4, as it stands, does not show this metric. Thus, it is in my opinion not a good validation for optical microscopy. As such, this performance metric cannot be interpreted.

- In the methods somewhere (I believe), the authors discuss upsampling to resolve this issues to a certain extent, but do not show this at all.

Already in the original submission, we stated explicitly that “We have also explored the benefits of incorporating upsampling layers into the DAMN convolutional architecture, further extending its capabilities….”, lines 209 – 301, in the main text of the original manuscript. The whole section “Astronomical and localization microscopy” then shows and discusses in detail the application of this upsampling model to astronomy and biology datasets. So, we have to disagree with the Reviewer’s comment.

In the revised manuscript, we improved the DAMN (with upsampling) demonstration on the astronomy dataset and included a new biology dataset of nuclear pore complexes. We have also improved the quantitative comparison of the DAMN results with respect to (a partial) ground truth available in these datasets and with respect to the other well-established methods.

I thank the authors for the clarification here.

- The SOSplugin reconstruction of the figure 6 dataset (b) is flawed in multiple ways. First of all, the SMLM challenge provides GT localizations, which can be used as reference; secondly, visualization of single molecules as scatter plots is a very flawed method – should be considering the loc. Uncertainty and visualization via upsampled gaussians, etc.

We do not mean to be blunt, but the SMLM challenge certainly does not (and cannot) provide ground truth localization for *experimental* data. It might provide ground truths for simulated data; however, the dataset used in our work is experimentally acquired, i.e., without ground truths.

Furthermore, although the visualization of the SOS Plugin result presented in our original submission might be flawed, it was presented in the exact same way in the SMLM challenge repository. However, to accommodate Reviewer #2, we redone the visualization of the SOS Plugin results based on their recommendation in the revised manuscript. Nevertheless, this changes virtually nothing in comparison of the methods.

Right. The authors are correct that they used an experimental dataset of the SMLM challenge, and I apologize that I missed this point.
I change my question to: why did the authors use an experimental dataset of the SMLM challenge (now properly mentioned in the text, not before), while the SMLM challenge was conceived to create “**synthetic data** that represent three-dimensional structures modeled after biological components, taking excitation parameters, noise sources, point-spread functions and pixelation into account” (taken literally from the cited paper).

The SMLM challenge was specifically created to have realistic simulated data with GT available to test SMLM algorithms on. It is specifically created so that authors (like these) that have new algorithms (like this manuscript) have realistic data to test their algorithm on.

So, let me rephrase my point:
First of all, the SMLM challenge provides GT localizations, which can be used as reference. The authors should use GT-available data from this challenge.

I appreciate that the authors have refrained from using scatter plots from the SOS plugin. I disagree that it changes nothing, as the data is much more interpretable from a human visual perspective.

- Finally, combining methods from astronomy and SMLM is not novel - in fact, one of the first SMLM reconstruction methods has made this connection ~15 years ago - and this paper is not mentioned here (Holden, Uphoff, Kapanidis, Nat Meth, 2011).

We agree with the Reviewer that our work is not the first super-resolution analysis using datasets from both astronomy and biology (and SMLM particularly). However, we do not claim this in our manuscript. The novel core idea of our work is that we can approach super-resolution in these (and even more) fields with a single model without (re)calibration, fine-tuning, or retraining.

Specifically, the work of Holden et al. Nature Methods 8, 279 (2011) presents the observation that an astronomy software package (DAOPHOT II) for model-based PSF fitting and star localization can be used for STORM data, i.e., sparse blinking molecule datasets, after specific PSF fitting and careful parameter tuning. However, they do not show any application of the software to both astronomy and biology data with the same setting (same calibration, same set of tuning parameters). Also, they do not demonstrate and compare the performance of the software for single-frame imaging methods. Because of these reasons, the work by Holden et al. does not differ at all from other multi-emitter fitting approaches, such as ThunderSTORM [Ovesny et al. Bioinformatics, 30, 2389 (2014)] or WTM [Takeshima, et al. Journal of Microscopy 271, 266 (2018)]. The latter actually shows that the performance of DAOSTORM (based on DAOPHOT II) is not optimum for higher density samples. Altogether, multi-emitter fitting approaches are not device-agnostic in any way, regardless of whether they are based on astronomy or biology software packages. We included the relevant references in the revised manuscript.

I thank that the authors included this reference.

Other, more minor points:
- I feel the authors overstate the problems which have already been addressed in earlier work, especially given the issue of obtaining in situ PSFs from your set-up. Many methods exist to directly extract this from a single in situ dataset.

We must kindly disagree with the Reviewer. The vast literature on PSF characterization and simulation in super-resolution imaging states the opposite. It is true that for sufficiently sparse samples, the PSF can be obtained directly from a single in situ dataset. However, it does not mean that this approach provides accurate results and is applicable in general. The main problems are:
- dense samples where emitters are overlapping (basically anything other than SMLM),
- typical low SNR impacting the PSF reconstruction accuracy (everywhere),
- aberrations making the PSF shape complex and beyond exact modeling/fitting (everywhere),
- PSF variations over the field-of-view (everywhere) and in time, i.e., within a temporal stack (in SMLM).

There are hundreds of high-impact and highly-cited works on these issues, such as [Deschout, et al. Nature Methods 11, 253 (2014), Booth, et al. Microscopy 64, 251 (2015), von Diezmannet al. Chemical Reviews 117, 7244 (2017), Xu, et al. Nature Methods 17, 531 (2020), Fu, et al., Nature Methods 20, 459-568 (2023), Liu, et al. Nature Methods 21, 1082-1093 (2024), etc.].

Besides the mentioned fundamental issues, the PSF calibration of super-resolution methods means that the reconstruction or localization procedure has to be fine-tuned or, in the case of prevailing deep learning methods, retrained for each sample or part of the sample or stack. Also, any change or (mis)alignment of an imaging setup calls for a new PSF calibration and training to keep the optimum performance.

And this is exactly the benefit of our novel device-agnostic approach. With this approach, practitioners in many fields of optical imaging (astronomy, microscopy, etc.) are not required to perform calibration procedures and train specific models. Instead, they are provided with a single trained model that works out of the box for a wide variety of samples and imaging systems.

One can naively expect that a super-resolution approach calibrated and trained for a specific sample and optical device would easily outperform our device-agnostic approach. Surprisingly, this is not the case. The main reasons DAMN performs so well are the strong generalization ability of the model (a direct result of device-agnostic training) and the fact that the sample and imaging parameters vary across the field of view (FOV) and may also change over the course of data acquisition. Specifically: (1) the emitter concentration is not constant across the FOV; (2) the point spread function varies due to inherent optical-system properties, typically resulting in more aberrations and broader point spread functions in the outer regions of the FOV; (3) the background is not homogeneous due to setup performance or sample properties (e.g., nonuniform background fluorescence); and (4) the signal-to-

noise ratio varies for similar reasons. State-of-the-art methods, particularly deep-learning approaches trained on specific parameter values (or calibration data), cannot generalize sufficiently to this variability. In contrast, our device-agnostic model is trained across an extremely large parameter range and can generalize even beyond the training data. Consequently, our method handles these inhomogeneities effectively and produces superior results.

I feel the authors misinterpret me. I don't want to state that in my opinion, by definition, specifically-trained models should outperform general ones. I stated that obtaining in situ PSFs is not specifically challenging anymore.

The problems that the authors claim here:
- dense samples where emitters are overlapping (basically anything other than SMLM),
- typical low SNR impacting the PSF reconstruction accuracy (everywhere),
- aberrations making the PSF shape complex and beyond exact modeling/fitting (everywhere),
- PSF variations over the field-of-view (everywhere) and in time, i.e., within a temporal stack (in SMLM).

Are not problems if you place fluorescent beads on your microscope (fixes point 1, 2), and fit with some proper algorithm (for instance the cited Liu, et al. Nature Methods 21, 1082-1093 (2024) , fixes point 3 and 4).
PSFs do not vary over time unless optical components are moving over time.

The rest of the rebuttal text on this point is moot. I stated "I feel the authors overstate the problems which have already been addressed in earlier work, especially given the issue of obtaining in situ PSFs from your set-up.". The response starting at "Besides the mentioned fundamental issues" is only about why a general model is good (which I agree with, and never stated to have a problem with in my point).

- Figure 2b and 2c, I believe, look at the same core problem. With increased PSF width, there is a problem of overlapping emitters if the emitter density is kept stable, which results in the sharp increase in error of the DAMN model.

Yes, indeed, Figures 2b and 2c show a similar scaling in the performance of super-resolution reconstruction methods with increasing overlap of emitters. In Figure 2b, the number of emitters is constant in the image (N = 50). and the PSF width is increasing. In Figure 2c, the PSF width is fixed (sigma = 2 px) while the number of emitters in the image is increasing. These two scalings are complementary but not identical. Some methods can cope better with a high number of "sharp" emitters than a few very "blurry" emitters, and vice versa. This is the reason why we keep both panels in Figure 2.

In the new version of the manuscript, we added two new reference methods in Figure 2, namely TV Richardson-Lucy and Deep-STORM. Together with the notorious Richardson-Lucy, these three methods demonstrate various sensitivity to the number of emitters, PSF width and shape, and SNR. For all combinations of parameters, DAMN outperforms these three methods. For extreme cases, such as excessively large PSF or number of emitters larger than 700 in the 50x50 image, all methods saturate at mean absolute error ≈ 1. We deliberately chose to plot these graphs up to those extreme cases to show the ultimate limits of our particular model. To get some perspective on what the case with N = 200 emitters looks like, see Figure 4b.

I appreciate the authors effort on this, and it improves the manuscript.

- I'm unsure why the Richardson-Lucy deconvolution isn't applied on the fig6 data as well, seeing as it's the author's go-to comparison method up to that point.

First, let us note that in the revised manuscript, the iterative total-variation regularized Richardson-

Lucy and the deep learning Deep-STORM are also used as reference methods in Figure 2 and Figure 4. It means that Deep-STORM is now also a go-to comparison method.

The Richardson-Lucy does not inherently incorporate upsampling, so the output reconstructed image would be of the same size as the input image. In contrast, Deep-STORM as well as DAMN both contain upsampling, so their comparison is fair.

Perfect, this satisfies my comment.

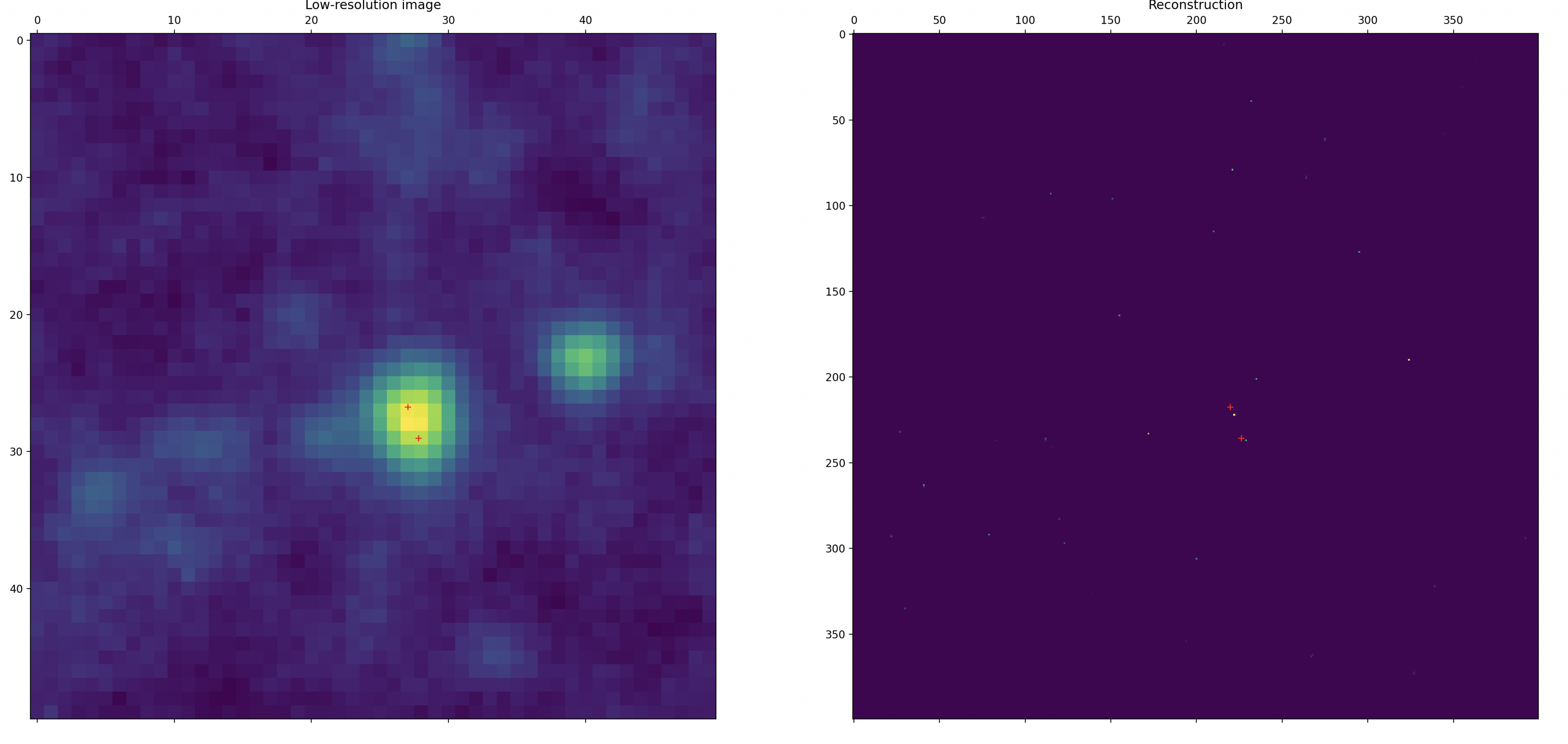
Low-resolution image
0
10
20
30
40
Reconstruction
0
50
100
150
200
250
300
350
